# Small molecule inhibitor of orphan GPCR dimerization improves host defense and blood pressure control in mice

**DOI:** 10.1172/JCI203162

**Published:** 2026-08-03

**Authors:** Jeonghyeon Kwon, Margherita Persechino, Jingchen Shao, Jamal Shamsara, Birgit Spitznagel, Isabelle Salwig, Miloslav Sanda, Stefan Offermanns, Peter Kolb, Nina Wettschureck

**Affiliations:** 1 Department of Pharmacology, Max Planck Institute for Heart and Lung Research, Bad Nauheim, Germany.; 2 Department of Pharmaceutical Chemistry, Philipps-Universität Marburg, Marburg, Germany.; 3 Department of Cardiac Development and Remodeling and; 4 Biomolecular Mass Spectrometry, Max Planck Institute for Heart and Lung Research, Bad Nauheim, Germany.; 5 German Center for Cardiovascular Research (DZHK), Partner Site Rhine-Main, Frankfurt am Main, Germany.; 6 Cardiopulmonary Institute (CPI), Goethe University, Frankfurt am Main, Germany.; 7 Centre for Molecular Medicine, Medical Faculty, Goethe-University Frankfurt, Germany.

**Keywords:** Immunology, Vascular biology, G protein-coupled receptors, Pharmacology, Signal transduction

## Abstract

Orphan GPCRs of the GPRC5 family regulate macrophage activity and vascular contractility by dimerizing with other GPCRs, but pharmacological modulation of this process has not been explored. We previously identified the dimerization interface of receptor GPRC5B and show here that both its mutation and inhibition by a decoy peptide disturbed the interaction with the prostaglandin E2 receptor EP2 in macrophages, resulting in reduced EP2 signaling, enhanced migration and phagocytosis, and protection from bacterial peritonitis in mice. Furthermore, we show that a similar interface exists in related receptor GPRC5C, and, the same as in GPRC5B, mutation or inhibition by decoy peptide improved host defense. Through a virtual docking screen, we identified a small molecule inhibitor of both GPRC5B and GPRC5C dimerization, K303MP20, and showed that it reduced EP2 signaling, enhanced macrophage activity, and improved host defense in bacterial peritonitis and influenza A infection. Interestingly, K303MP20 not only blocked dimerization between GPRC5B/C and EP2, but also with prostacyclin receptor IP and angiotensin II receptor AT1, resulting in reduced AT1-dependent contraction and enhanced IP-dependent relaxation in human and murine smooth muscle cells. In vivo, K303MP20 did not affect basal blood pressure, but protected mice from angiotensin II–induced hypertension. Taken together, inhibition of orphan GPCR dimerization by small molecules is feasible and improves infection control and arterial hypertension.

## Abstract 2

Pharmacological blockade of GPRC5B/GPRC5C dimerization improves infection control and prevents arterial hypertension.

## Introduction

G-protein–coupled receptors (GPCRs) are with approximately 800 members the largest family of transmembrane receptors in eukaryotes; they transduce signals elicited by diverse stimuli such as hormones, neurotransmitters, local mediators, olfactory and metabolic cues, and light (1, 2). During the last decades, GPCRs have become a major class of drug targets, but even though 36% of approved drugs act as GPCR agonists or antagonists, only a third of the approximately 360 nonsensory GPCRs are currently therapeutically targeted (3, 4). One reason for this incomplete pharmacological coverage is the fact that around 100 GPCRs are still considered “orphan”, i.e., their physiological signals have not been identified and their functions are not well defined (4, 5). In order to exploit the pharmacological potential of orphan GPCRs, large efforts have been directed at deorphanization or identification of nonendogenous ligands, in some cases successfully; for orphan receptors such as GPR6, GPR52, or GPR84, ligands have been identified and their therapeutic merit is being determined in clinical trials (3). For other orphan GPCRs, ligands are still unknown, and reasons for this elusiveness might be their coupling to noncanonical signaling pathways, the lack of appropriate cofactors in the screening system, low receptor expression or stability, or the absence of a matching ligand in experimental screening libraries. In addition, some GPCRs were shown to function in a ligand-independent manner by dimerizing with other GPCRs (6, 7). The class C GPCR GABA_B2_, for example, is not activated by neurotransmitter GABA; its function is to facilitate membrane trafficking of the ligand-binding GABA_B1_ receptor and to mediate G-protein coupling of the GABA_B1_/GABA_B2_ heterodimer (6, 7). Other class C receptors may also function primarily as dimerization partners, for example the class C orphan receptors GPRC5B and GPRC5C.

Initially discovered as retinoic acid–induced genes (8), both GPRC5B and GPRC5C are broadly expressed in central nervous system and mesenchymal cells, and studies in global knockout mice revealed mild neurological defects as well as subtle changes in the metabolic and renal systems (9–12). Even though GPRC5 family members lack typical dimerization features such as a Venus flytrap motif, they can form functionally relevant dimers; we previously showed that GPRC5B dimerizes in smooth muscle cells (SMCs) with the prostacyclin receptor IP, thereby inhibiting IP-mediated effects such as relaxation and differentiation (13). GPRC5B also dimerizes with other prostanoid receptors, for example the PGE_2_ receptor EP2. In macrophages, inactivation of GPRC5B diminishes EP2-dependent antiinflammatory signals, resulting in enhanced macrophage activation and consecutively improved protection again bacterial infection (14). The related receptor GPRC5C can also dimerize with prostanoid receptors IP and EP2, and, in addition, it interacts with the Angiotensin II (AngII) receptor AT1, resulting in a facilitation of ligand binding and AT1-dependent signaling (15). Using in silico modelling and protein:protein docking, we identified 7 amino acids in GPRC5B transmembrane domain 2 as determinants of the GPRC5B/EP2 dimerization: F97, L101, L104, F111, Q116, D118, and L128. Studies in HEK293T (HEK) cells showed that alanine mutation of all 7 residues (G5b-mut7) did not change plasma membrane localization of GPRC5B, but abrogated GPRC5B/EP2 dimerization and GPRC5B-mediated facilitation of EP2 signaling (14). Furthermore, we found that a subset of 3 residues, F97, L101 and L104, termed “set 1,” was sufficient to fully reproduce the effects of G5b-mut7 (14). We had thus pinpointed the dimerization site to a relatively limited area close to the extracellular end of transmembrane helix (TM) 2. Interestingly, this site had also emerged as a commonly available extra-helical pocket in our previous analysis of the “pocketome” of GPCRs (16). While several of these extra-helical sites have been found to be able to host small-molecule ligands (17), it was less clear whether the sites could also function as dimerization interfaces — at least in this case, they do. Despite the fact that there are scarcely any reports on the identification of ligands against extra-helical sites through structure-based methods without relying on prior knowledge of other ligands, we wished to exploit the dimerization site for the structure-based in silico identification of small-molecule dimerization inhibitors, and we have done so in the present manuscript.

In summary, here, we explore whether mutation of the GPRC5B interface is affecting macrophage function in vivo and whether similar mechanisms apply to the related receptor GPRC5C. In search of small molecule inhibitors of the process, we performed in silico docking studies and identified several small molecules as inhibitors of GPRC5B/C-dimerization and GPRC5B/C-mediated modulation of EP2, IP, and AT1 signaling in vitro and in vivo, with the compound K303MP20 as the most potent among them.

## Results

### Enhanced macrophage activity in mice expressing a dimerization-deficient GPRC5B mutant.

**Figure 1:**
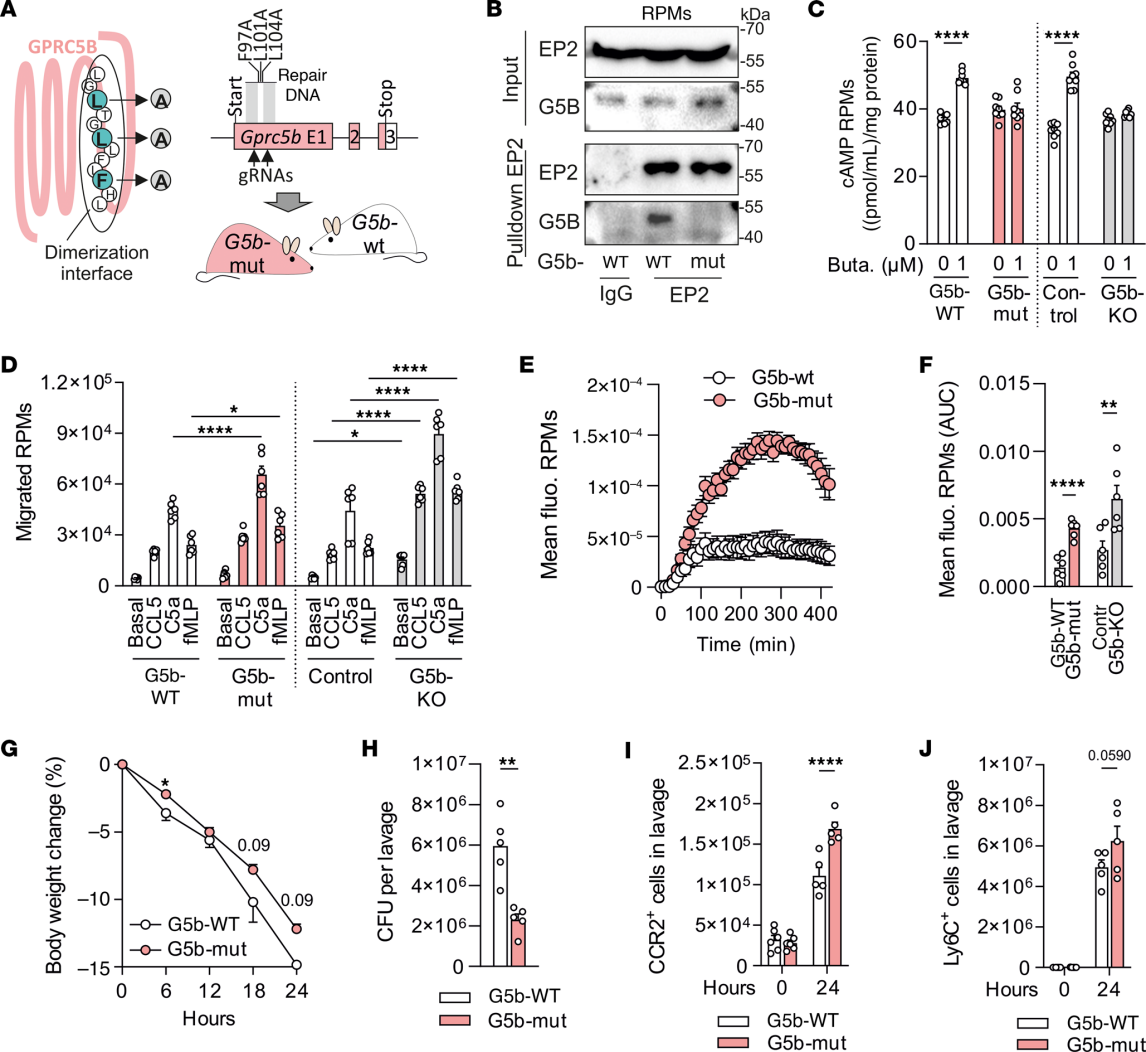
Enhanced macrophage activity in mice expressing a dimerization-deficient GPRC5B mutant (G5b-mut). (**A**) Generation of G5b-mut mice: (left) GPRC5B amino acids F97, L101, and L104 were mutated to alanine, indicated by the letter A; (right) localization of guide RNAs (gRNA) and repair DNA within the *Gprc5b* allele; resulting founder mice were used to produce Gprc5b^mut/mut^ mice (G5b-mut) and Gprc5b^wt/wt^ mice (G5b-wt). (**B**) Coimmunoprecipitation of endogenous GPRC5B (G5B) with EP2 in RPMs from G5b-wt or G5b-mut mice; IgG-mediated precipitation in G5b-wt RPMs as negative control. (**C**) Butaprost-induced cAMP production was determined by ELISA in RPMs from G5b-wt and G5b-mut mice (cells from control mice and M-G5b-KOs as reference) (*n* = 8). (**D**) Basal and chemokine-induced transwell migration of G5b-wt and G5b-mut RPMs; cells from control mice and M-G5b-KOs as reference (*n* = 6). (**E** and **F**) Phagocytosis of *E*. *coli* bioparticles in G5b-wt and G5b-mut RPMs. Exemplary traces (**E**) and statistical evaluation of area under the curve (AUC, **F**); cells from control and M-G5b-KOs as reference (*n* = 6). (**G**–**J**) Fecal peritonitis in G5b-wt and G5b-mut mice: body weight change (**G**), bacterial colony-forming units (CFU) in peritoneal lavage fluid 24 hours after injection of fecal bacteria (**H**), numbers of CD11b^+^, F4/80^lo^, MHCII^+^, CCR2^+^ macrophages (**I**), and CD11b^+^, Ly6G^–^, Ly6C^+^ monocytes (**J**) before and 24 hours after injection of fecal bacteria (*n* = 5–6). For gating strategy, see section 4 of Supplemental Materials. Data are means ± SEM; comparisons between treatment groups were performed using 2-way ANOVA with Šídák’s (**C**, **G**, **I**, and **J**) or Tukey’s (**D**) multiple comparisons test or unpaired, 2-tailed *t* test (**F** and **H**). *n*, number of independent experiments or mice; *P < 0.05; **P < 0.01; ****P < 0.0001.

Our previous studies in HEK cells showed that alanine mutation of GPRC5B residues F97, L101 and L104 (“set 1”) strongly reduced GPRC5B/EP2 dimerization and GPRC5B-mediated facilitation of EP2 signaling (14), while plasma membrane localization of the mutant was not altered (Supplemental Figure 1; supplemental material available online with this article; https://doi.org/10.1172/JCI203162DS1). The in vivo consequences of this targeted mutation, however, were unknown. To address this issue, we used CRISPR/Cas9 genome editing to generate a mouse line in which these 3 amino acids were mutated to alanine (henceforth “G5b-mut”) (Figure 1A and Supplemental Figure 2, A and B). The mutation did not alter GPRC5B expression or intracellular localization in resident peritoneal macrophages (RPMs) (Supplemental Figure 2, C–F), but the interaction with the EP2 receptor, assessed by coimmunoprecipitation, was strongly reduced in G5b-mut RPMs (Figure 1B). Loss of GPRC5B/EP2 dimerization is expected to mimic the GPRC5B knockout phenotype of reduced EP2 signaling (14), and we indeed found that G5b-mut RPMs showed the same reduction of cAMP production in response to EP2 agonist butaprost as RPMs from mice with myeloid-specific GPRC5B deficiency (M-G5b-KOs) (Figure 1C). In line with this, G5b-mut RPMs partly recapitulated the increased chemokine-induced migration and phagocytosis observed in GPRC5B-deficient RPMs (Figure 1, D–F). Also, bone marrow–derived macrophages (BMDMs) from G5b-mut mice showed increased migration and phagocytosis, and the latter was associated with mildly increased cytokine expression in G5b-mut BMDMs (Supplemental Figure 3, A–D). As previously shown for M-G5b-KOs (14), these changes resulted in G5b-mut mice in reduced susceptibility in a model of fecal peritonitis, with less body weight loss and lower numbers of colony-forming bacteria in the peritoneal lavage after 24 hours (Figure 1, G and H). Furthermore, flow cytometric analysis of peritoneal fluid showed increased numbers of CCR2-positive macrophage and Ly6c-positive monocytes (Figure 1, I and J), whereas numbers of TIM4-positive RPMs were not clearly changed between genotypes (Supplemental Figure 3E). Since increased macrophage activation might enhance the risk of autoimmune disease, we determined susceptibility of G5b-mut mice in experimental autoimmune encephalomyelitis (EAE), a mouse model of multiple sclerosis. G5b-mut mice did not show clear changes with respect to body weight loss or neurological scores after immunization with myelin oligodendrocyte glycoprotein MOG_35-55_ (Supplemental Figure 3, F and G), and also histological analysis did not reveal significant differences in demyelination (Supplemental Figure 3H). Taken together, inhibition of GPRC5B dimerization by mutation of the GPRC5B interface enhances macrophage activity, resulting in improved host defense without clear effects on autoimmune neuroinflammation.

### GPRC5B decoy peptide blocks GPRC5B/EP2 dimerization and enhances macrophage activity in vitro and in vivo.

**Figure 2:**
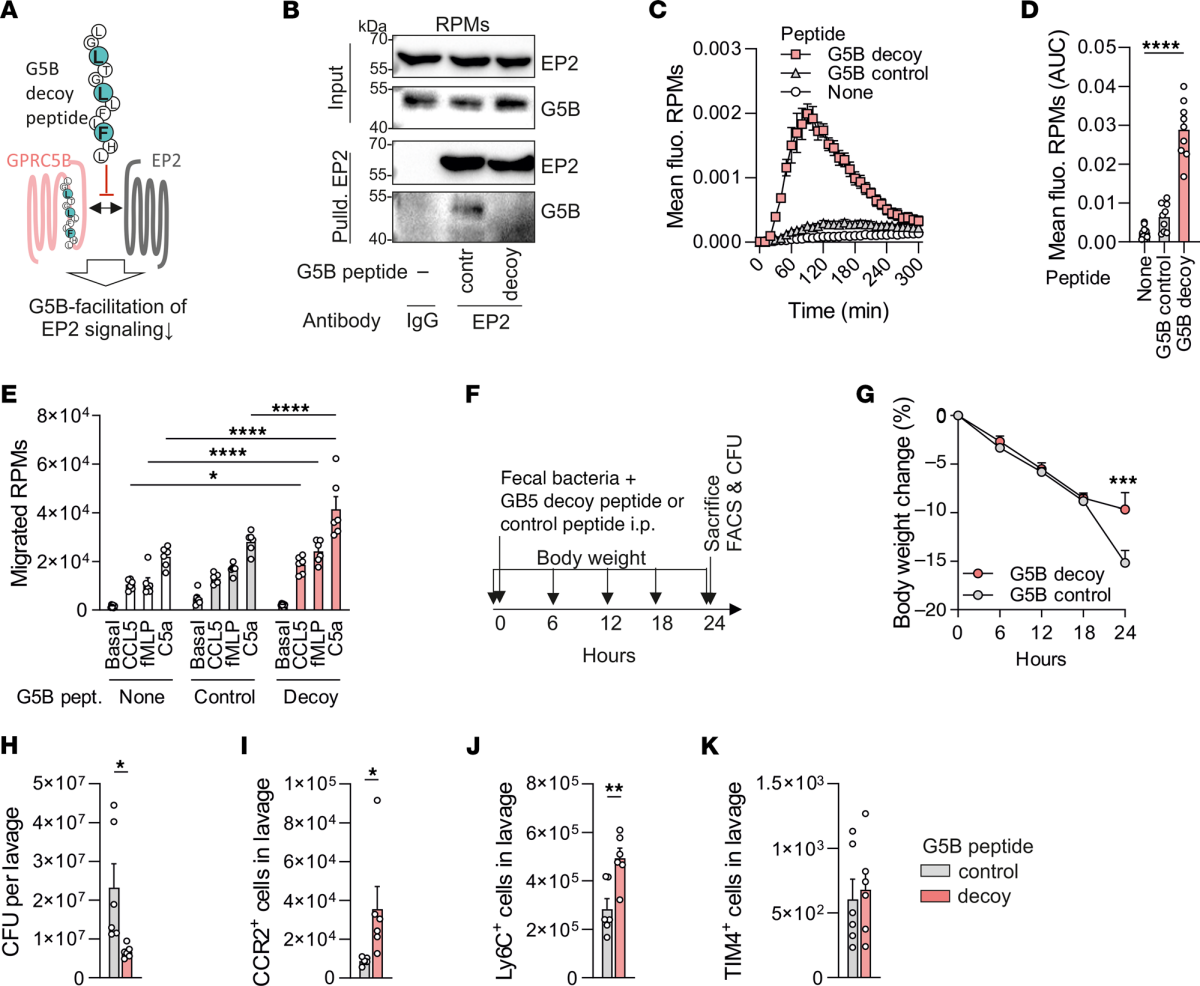
GPRC5B decoy peptide blocks GPRC5B/EP2 dimerization and enhances macrophage activity in vitro and in vivo. (**A**) Principle of decoy peptide-mediated inhibition of GPRC5B dimerization. (**B**) Coimmunoprecipitation of endogenous GPRC5B with EP2 in RPMs that were incubated for 1 hour with decoy peptide or scrambled control peptide (1 μM in all experiments); IgG-mediated precipitation as negative control. (**C** and **D**) Effect of GPRC5B decoy peptide and control peptide on phagocytosis of pHrodo *E*. *coli* bioparticles in RPMs; exemplary traces (**C**) and statistical evaluation of area under the curve (AUC, **D**) (*n* = 9). (**E**) Effect of peptides on chemokine-induced RPM migration was determined in transwell assays (*n* = 6). (**F**–**J**) Fecal peritonitis in peptide-treated mice; effect of peptides (100 μL of a 100 μM solution) on body weight change (**G**), bacterial CFU (**H**), numbers of CD11b^+^, F4/80^lo^, MHCII^+^, CCR2^+^ macrophages (**I**), CD11b^+^, Ly6G^–^, Ly6C^+^ monocytes (**J**), or CD11b^+^, F4/80^+^, MHCII^–^, TIM4^+^ RPMs (**K**) in peritoneal lavage fluid harvested 24 hours after injection of fecal bacteria (*n* = 6). Data are means ± SEM; comparisons between treatment groups were performed using 1-way ANOVA with Dunnett’s multiple comparisons test (**D**), 2-way ANOVA with Tukey′s (**E**) or Šídák’s multiple comparisons test (**G**), or unpaired, 2-tailed *t* test (**H**–**K**). *n*, number of independent experiments or mice; *P < 0.05; ***P < 0.001; ****P < 0.0001.

We previously showed that a decoy peptide mimicking the GPRC5B dimerization interface was able to interfere with the GPRC5B/EP2 interaction in GPCR-overexpressing HEK cells (Figure 2A) (14). In this peptide, a C-terminally amidated peptide consisting of amino acids 95–106 of GPRC5B (LHFLFLLGTLGL, containing “set 1” amino acids F97, L101, L104) was N-terminally fused to the HIV-TAT sequence to allow cell penetration (“G5B decoy”). As a control peptide, a scrambled version of the target sequence (FLTLLLLLFGHG) was fused to HIV-TAT (“G5B control”). Our previous HEK cell studies showed that G5B decoy, but not G5B control, inhibited coimmunoprecipitation and bioluminescence resonance energy transfer (BRET) between GPRC5B and EP2 and reduced GPRC5B-dependent facilitation of EP2 signaling (14). To test whether G5B decoy was able to disturb the interaction between endogenously expressed GPRC5B and EP2, we performed coimmunoprecipitation studies in RPMs and found that decoy peptide, but not scrambled control peptide, markedly reduced the interaction between the receptors (Figure 2B). As shown in G5b-mut mice, loss of the GPRC5B/EP2 interaction is expected to reduce EP2 signaling and thereby induce macrophage hyperactivation. In line with this, we found that decoy peptide, but not control peptide, strongly increased phagocytosis of *E*. *coli* particles in RPMs (Figure 2, C and D), and this effect was not present in GPRC5B-deficient macrophages (Supplemental Figure 4A). Furthermore, C5a- and fMLP-induced migration was facilitated by decoy peptide (Figure 2E). To determine the in vivo consequences of this hyperactivation, we investigated how decoy or control peptide affected the outcome of fecal peritonitis (Figure 2F). Decoy peptide–treated mice showed lower body weight loss than control mice 24 hours after bacteria inoculation (Figure 2G), and the number of colony-forming units in the lavage fluid was reduced (Figure 2H). Recruitment of CCR2-positive macrophages and Ly6c-positive monocytes was increased (Figure 2, I and J), while the number of TIM4-positive RPMs was not clearly changed (Figure 2K). In line with a GPRC5B-dependent mode of action, decoy peptide did not further reduce disease severity in M-G5b–KO mice (Supplemental Figure 4, B and C). These data show that decoy peptide can inhibit GPRC5B/EP2 dimerization in primary macrophages and reproduce the G5b-mut and GPRC5B-KO phenotypes of macrophage hyperactivity and improved host defense.

### GPRC5C resembles GPRC5B with respect to dimerization pattern, effect on cellular cAMP, and knockout phenotype.

**Figure 3:**
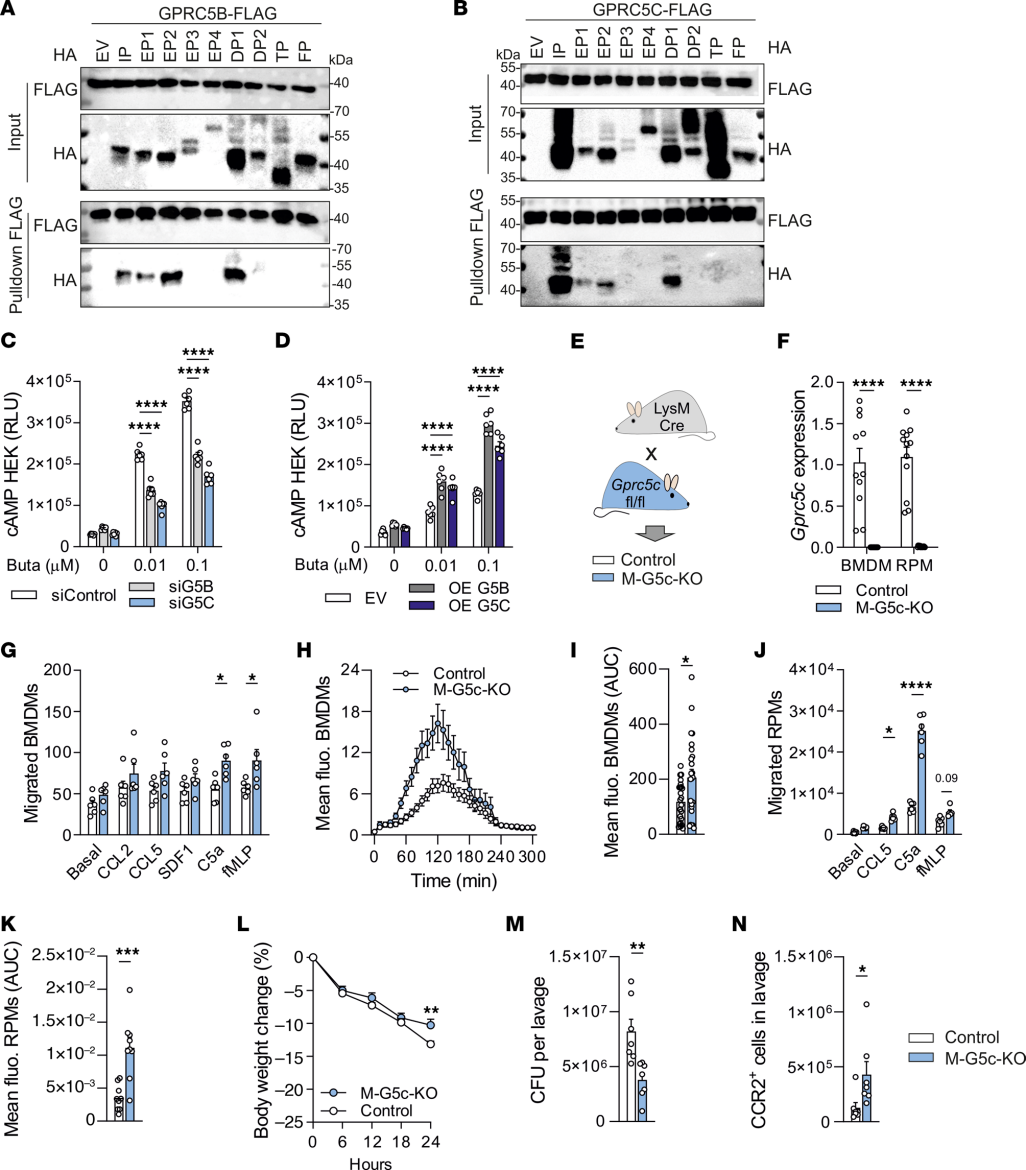
GPRC5C resembles GPRC5B with respect to dimerization pattern, effect on cellular cAMP, and knockout phenotypes. (**A** and **B**) Western blot detection of HA and FLAG signals in lysates of HEK cells expressing FLAG-tagged GPRC5B (left) or GPRC5C (right) in combination with different HA-tagged prostanoid receptors before (“input”) and after immunoprecipitation of GPRC5B-FLAG (“Pulldown FLAG”). (**C** and **D**) Butaprost-induced cAMP production was determined in HEK cells transfected with EP2, a cAMP GloSensor plasmid and either control siRNA (siControl) or siRNA directed against GPRC5B or GPRC5C (siG5B or siG5C) (**C**) or expression vectors encoding GPRC5B or GPRC5C (OE G5B, OE G5C; EV is empty vector) (**D**) (*n* = 6). (**E**) Generation of myeloid-specific GPRC5C-KOs (M-G5c-KO). (**F**) Knockout efficiency was determined by qRT-PCR (data normalized to *Gapdh* and controls set to 1) (*n* = 8, 10). (**G** and **J**) Transwell migration of M1 BMDMs (**G**) and RPMs (**J**) in response to different chemotactic factors (*n* = 6). (**H**, **I**, and **K**) Uptake of pHrodo *E.coli* bioparticles by M0 BMDMs (**H** and **I**; *n* = 24) and RPMs (**K**; *n* = 9). (**L**–**N**) Fecal peritonitis in control mice and M-G5c-KOs: Body weight change (**L**), number of bacterial CFU (**M**) and of CD11b^+^, F4/80^lo^, MHCII^+^, CCR2^+^ macrophages (**N**) in peritoneal lavage fluid harvested 24 hours after injection of fecal bacteria (*n* = 7). Data are means ± SEM; comparisons between genotypes were performed using 2-way ANOVA with Dunnett′s (**C**) or Šídák’s (**D**, **G**, **J**, and **L**) multiple comparisons test, or unpaired, 2-sided *t* test (**F**, **I**, **K**, **M**, and **N**). *n*, number of independent experiments or mice; RLU, relative luminescence units; *P < 0.05; **P < 0.01; ***P < 0.001; ****P < 0.0001.

GPRC5B is 1 of 4 members of the GPRC5 family, the other members are GPRC5A, GPRC5C, and GPRC5D. GPRC5B and GPRC5C are 50% identical, whereas GPRC5A and GPRC5D exhibit lower sequence homology to GPRC5B (18). In our previous work, we showed that GPRC5B not only interacts with IP and EP2, but also with other prostanoid receptors such as EP1 and DP1 (14), and, here, we systematically investigated whether other GPRC5 family members share GPRC5B’s ability to dimerize with these prostanoid receptors. Coimmunoprecipitation studies in HEK cells showed that GPRC5C displayed the same interaction pattern as GPRC5B (Figure 3, A and B), whereas GPRC5A and D differed strongly (Supplemental Figure 5, A and B). Further studies in HEK cells showed that GPRC5C modulated EP2 signaling in the same way as GPRC5B: cAMP production induced by the EP2 agonist butaprost was reduced after knockdown of GPRC5B or GPRC5C (Figure 3C and knockdown efficiency in Supplemental Figure 5, C and D) and enhanced after overexpression (Figure 3D). This led us to investigate whether GPRC5C-deficient macrophages were also hyperactive. We generated myeloid-specific GPRC5C-deficient mice by crossing the *LysM*Cre line (19) to mice carrying floxed *Gprc5c* alleles (15), resulting in myeloid-specific GPRC5C-KOs (M-G5c-KOs) (Figure 3, E and F). As previously observed in M-G5b-KOs (14), GPRC5C-deficient BMDMs also showed increased migration and phagocytosis (Figure 3, G–I), and the same was true in RPMs (Figure 3, J and K). In line with this, M-G5c–KOs showed reduced body weight loss and improved bacterial clearance in the fecal peritonitis model, and the recruitment of CCR2-positive macrophages was increased (Figure 3, L–N). In summary, GPRC5C displays the same dimerization pattern as GPRC5B in HEK cells, and its inactivation in macrophages reproduces the hyperactivity phenotype observed in GPRC5B-deficient macrophages.

### GPRC5C-mediated effects on cAMP production and macrophage activity are blocked by interface mutation and decoy peptide.

**Figure 4:**
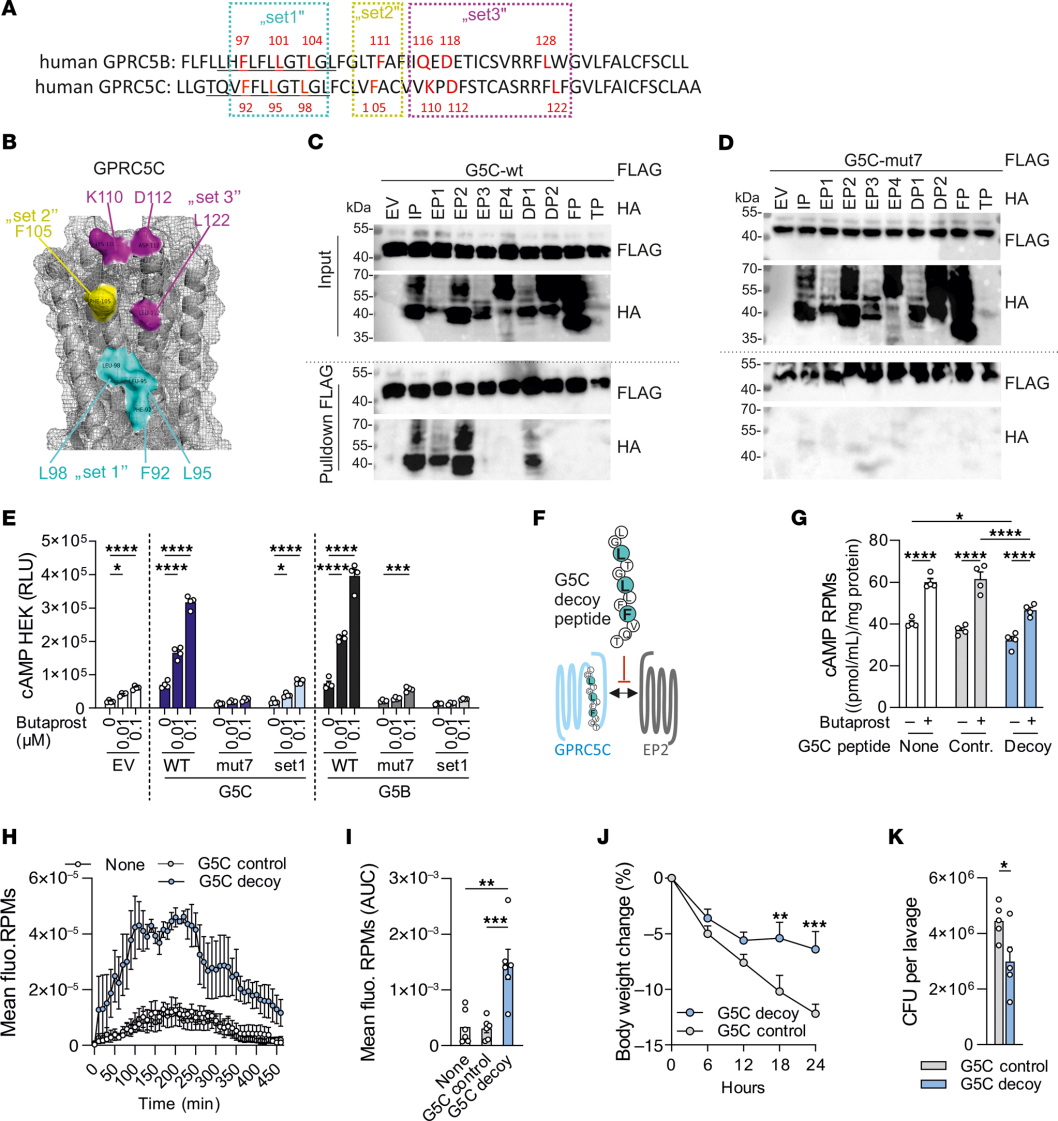
GPRC5C-mediated effects on cAMP production and macrophage activity are blocked by interface mutation and decoy peptide. (**A**) Amino acid sequences in the putative interaction interfaces. (**B**) Localization of 3 sets of residues predicted to mediate the interaction. (**C** and **D**) Western blot detection of HA and FLAG signals in lysates of HEK cells expressing FLAG-tagged WT (G5C-wt, **C**) or mutated (**D**, G5C-mut7) GPRC5C in combination with different HA-tagged prostanoid receptors before (“input”) and after immunoprecipitation of GPRC5B-FLAG (“Pulldown FLAG”). (**E**) Butaprost-induced cAMP production in HEK cells transfected with cAMP GloSensor plasmid, HA-EP2, and WT or mutant GPRC5C (G5C, middle section) or GPRC5B (G5B, right section). Mutants contain either alanine mutations of all 7 residues (“mut7”) or only of set1 residues (“set1”) as indicated in **A** and **B** (*n* = 4). (**F**) Principle of decoy peptide–mediated inhibition of GPRC5C dimerization. (**G**) Effect of GPRC5C decoy peptide and control peptides (1 μM each) on butaprost-induced cAMP production in RPMs (*n* = 4). (**H** and **I**) Peptide effect on phagocytosis of pHrodo *E*. *coli* bioparticles in RPMs; exemplary traces (**H**) and statistical evaluation of AUC (**I**) (*n* = 6). (**J** and **K**) Fecal peritonitis model: effect of peptides (100 μl of a 100 μM solution) on body weight change (**J**) and bacterial CFU in peritoneal lavage fluid harvested 24 hours after injection of fecal bacteria (**K**) (*n* = 5). Data are means ± SEM; comparisons between treatments were performed using 2-way ANOVA with Dunnett’s (**E**), Tukey’s (**G**), or Šídák’s (**J**) multiple comparisons test, 1-way ANOVA with Tukey’s multiple comparisons test (**I**), or unpaired, 2-sided *t* test (**K**). *n*, number of independent experiments or mice; RLU, relative luminescence units; *P < 0.05; **P < 0.01; ***P < 0.001; ****P < 0.0001.

The amino acids making up the interaction interface of GPRC5B are largely conserved in GPRC5C (Figure 4A), and can — the same as in GPRC5B (14) — be subdivided in 3 subsets of residues, sets 1–3 (Figure 4, A and B). To investigate whether these residues control GPRC5C dimerization, we first generated a GPRC5C mutant in which all 7 amino acids were mutated to alanine (G5C-mut7). Same as WT GPRC5C, G5C-mut7 was mainly localized at the plasma membrane in HEK cells (Supplemental Figure 6, A and B), but the mutant had lost its ability to coprecipitate prostanoid receptors (Figure 4, C and D). In line with a disturbed GPRC5C/EP2 interaction, G5C-mut7 no longer facilitated EP2 signaling in HEK cells, thereby phenocopying the effect of G5B-mut7 (G5B-mut7) (14) (Figure 4E). As previously shown for GPRC5B, mutation of set 1 amino acids F92, L95, and L98 (G5C-set1) was sufficient to abrogate the facilitating effect of GPRC5C on EP2 signaling and return them to levels observed in empty vector-transfected cells (Figure 4E).

We next explored whether a decoy peptide mimicking the interacting region of GPRC5C was able to block GPRC5C-mediated facilitation of EP2 signaling in macrophages (Figure 4F). We designed a C-terminally amidated peptide consisting of the HIV-TAT cell–penetrating amino acid sequence (YGRKKRRQRRR) fused to amino acids 89–100 of GPRC5C (TQVFFLLGTLG) (“G5C decoy peptide”). As a control peptide, we fused a scrambled version of the target sequence (QLLGFLTFGTLV) to HIV-TAT. The GPRC5C decoy peptide reduced butaprost-induced cAMP production in RPMs and BMDMs, whereas a control peptide was without clear effect (Figure 4G and Supplemental Figure 6C). Furthermore, decoy peptide increased phagocytosis (Figure 4, H and I), and the facilitating effect on phagocytosis was absent in GPRC5C-deficient macrophages (Supplemental Figure 6D). We also tested the efficiency of GPRC5C decoy peptide in the peritonitis model and found reduced body weight loss, improved bacterial clearance, and enhanced recruitment of CCR2-positive macrophages in decoy peptide–treated mice (Figure 4, J and K, and Supplemental Figure 6, E and F). Taken together, GPRC5C and GPRC5B share a partially conserved dimerization interface that can be blocked by decoy peptides to induce macrophage hyperactivity and improve host defense in the fecal peritonitis model.

### In silico docking to identify small molecule inhibitor of GPRC5B dimerization.

**Figure 5:**
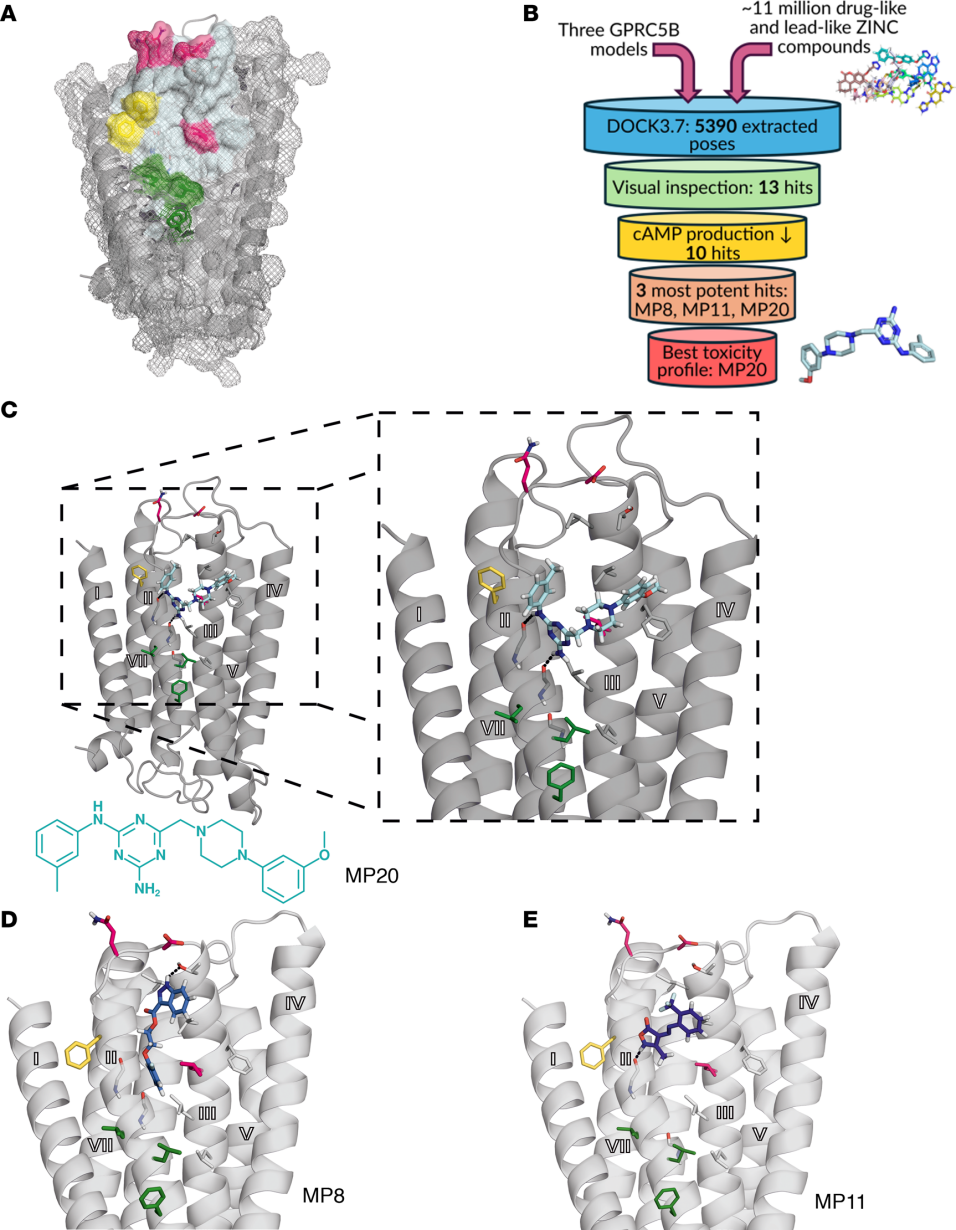
In silico screening for small molecule inhibitors of GPRC5B dimerization. (**A**) Depiction of GPRC5B, indicating the mutated residues as in Figure 4B and showing the area defined as docking zone as light grey solid surface. (**B**) Graphical overview of the docking process and consecutive analyses. (**C**) Predicted binding mode of MP20 in its GPRC5B model (grey). (**D** and **E**) Predicted binding modes of MP8 and MP11, respectively, in their GPRC5B model (light grey). TM helices are labelled with Roman numerals. Interacting residues are depicted as thick lines, while mutated residues are shown in green (set 1), yellow (set 2), and red (set 3).

In the next step, we used in silico docking calculations to identify small molecules that can interact with the interface and thereby interfere with dimerization. The dimerization site targeted in the docking calculations corresponds with the site that we termed KS2 in our previous work on the “pocketome” of GPCRs (16) and is located around amino acids F97A, L101A, and L104A. Docking calculations using the DOCK3.7 software were executed with 3 different models of GPRC5B generated as reported in the Methods section: (a) the GPRC5B model generated with MODELLER based on mGluR1 and mGluR5; (b) the AlphaFold model of GPRC5B from the AlphaFold protein structure database; and (c) the GPRC5B model generated with SWISS-MODEL using mGluR2 as a template (see Methods and Supplemental Figure 15). Each of the models was used in a docking screen with the “lead-like” and “drug-like” subsets of the ZINC20 molecule database, together containing 11 million compounds (Figure 5, A and B). The top-ranked clustered poses per docking were visually inspected (5,390 poses in total), and 21 compounds were selected for further testing, of which 13 could be acquired from their respective vendors (Supplemental Table 1). Exemplary docking-predicted poses for 3 compounds (K303MP8, 11, and 20) are shown in Figure 5, C–E. Retrospective enrichment calculations are presented in Supplemental Data File 4 “Performance metrics of docking calculations.”

### Functional characterization of small molecule inhibitors of GPRC5B/C dimerization.

**Figure 6:**
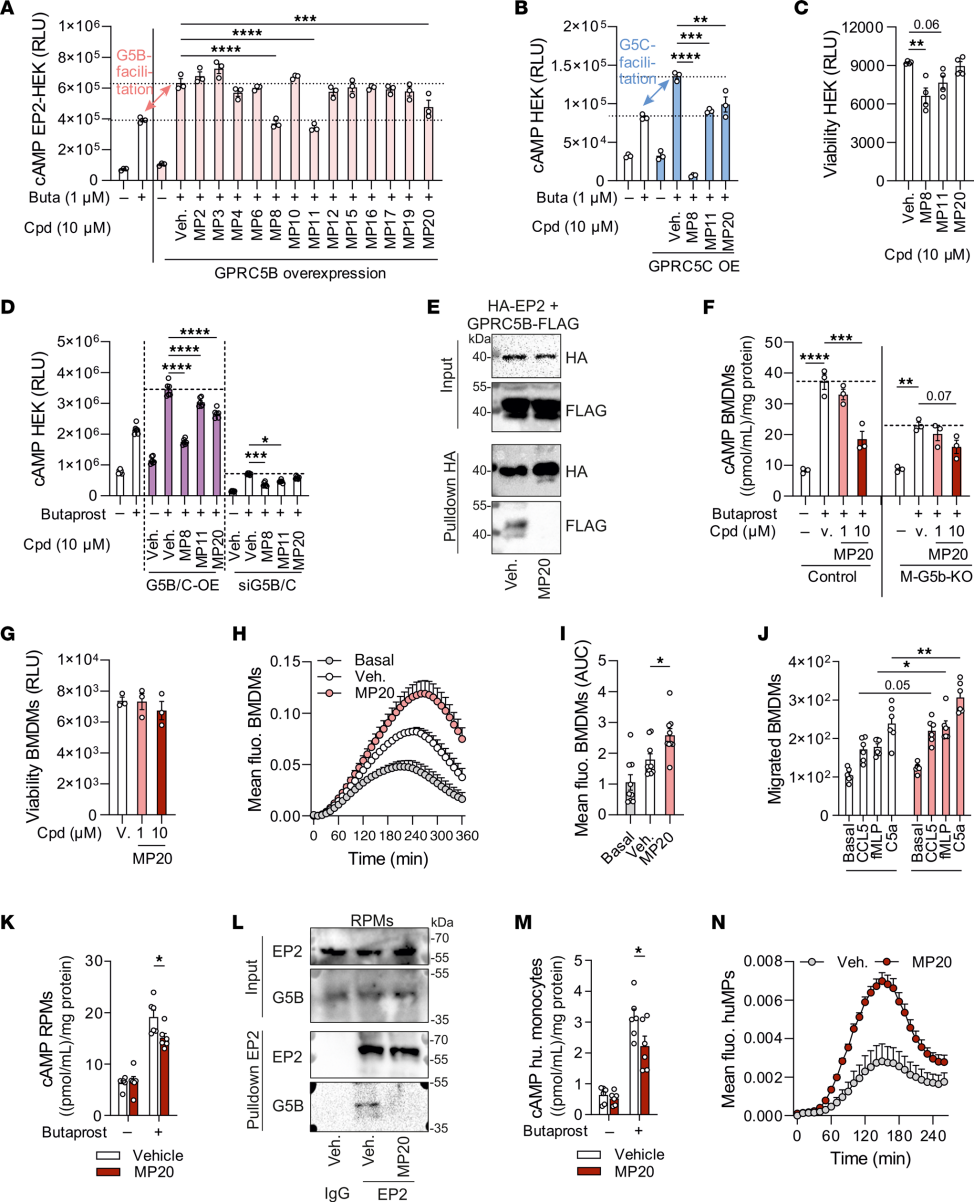
Functional characterization of small molecule inhibitors of GPRC5B/C dimerization. (**A** and **B**) Effect of compounds (10 μM each) on butaprost-induced cAMP production in HEK cells transfected with cAMP GloSensor plasmid, HA-EP2, and GPRC5B-FLAG (**A**) or GPRC5C-FLAG (**B**) as indicated (*n* = 3). (**C**) Viability of HEK cells (transfected as in **B**) after treatment with selected compounds for 1 hour (n=4). (**D**) Effect of compounds (10 μM each) on butaprost-induced cAMP production in HEK cells transfected with cAMP GloSensor plasmid, HA-EP2, GPRC5B/GPRC5C-FLAG (G5B/C-OE), or siRNAs directed against GPRC5B and GPRC5C (siG5B/C) (*n* = 3). (**E**) Coimmunoprecipitation of GPRC5B-FLAG with HA-EP2 from HEK cells treated with vehicle or MP20 (10 μM) for 1 hour. (**F** and **G**) Effect of MP20 (1 or 10 μM, 1 hour) on butaprost-induced cAMP production (**F**) and viability (**G**) in BMDMs (*n* = 3). (**H** and **I**) MP20 effect (1 μM each) on phagocytosis of pHrodo *E*. *coli* bioparticles in M0 BMDMs; exemplary traces (**H**) and statistical evaluation of AUC (**I**) (*n* = 9). (**J**) MP20 effect (1 μM each) on chemokine-induced BMDM migration (*n* = 6). (**K**) Effect of MP20 (10 μM) on butaprost-induced cAMP production in RPMs (*n* = 6). (**L**) Coimmunoprecipitation of endogenous GPRC5B with EP2 in RPMs that were incubated with vehicle or MP20 (10 μM) for 1 hour. (**M**) Effect of MP20 (10 μM) on butaprost-induced cAMP production in human blood monocytes (*n* = 6). (**N**) Effect of MP20 (1 μM) on phagocytosis of pHrodo *E*. *coli* bioparticles in human blood monocyte-derived macrophages (huMP) (*n* = 10; quantification in Supplemental Figure 7I). Data are means ± SEM; comparisons between treatments were performed using 1-way ANOVA with Dunnett′s (**A**–**E**, and **I**) or Tukey’s multiple comparisons test (**F** and **G**) or 2-way ANOVA with Šídák’s (**J**, **K**, and **M**) multiple comparisons test. RLU, relative luminescence units; V. or Veh., vehicle; *P < 0.05; **P < 0.01; ***P < 0.001; ****P < 0.0001.

We first tested whether candidate compounds were able to reduce GPRC5B-dependent facilitation of EP2 signaling in HEK cells. While no clear effects were observed at 1 μM compound concentration (Supplemental Figure 7A), preincubation with 10 μM compound significantly reduced the facilitating effect of GPRC5B overexpression on butaprost-induced cAMP production for 3 substances, K303MP8, K303MP11 and K303MP20 (Figure 6A; abbreviated to MP8, MP11, and MP20 in the following). These 3 compounds also significantly reduced GPRC5C-induced facilitation of EP2 signaling (Figure 6B). However, MP11 and especially MP8 diminished HEK cell viability, whereas MP20 was well tolerated (Figure 6C). Furthermore, MP8 and MP11, but not MP20, reduced butaprost-induced cAMP production even in GPRC5B/GPRC5C-knockdown cells, which, by themselves, have reduced butaprost responses (Figure 6D). Because of safety concerns and potential off-target effects, MP8 and MP11 were therefore set aside and further studies focused on MP20. We next investigated whether MP20 truly interferes with the interaction between EP2 and GPRC5B or GPRC5C and found that coimmunoprecipitation between GPRC5B/C and EP2 was strongly reduced by MP20 (Figure 6E and Supplemental Figure 7B). Furthermore, we studied the effect of MP20 on BRET. In line with a disturbed interaction, energy transfer from GPRC5B-wt-NLuc to EP2-mVenus was reduced by increasing concentrations of MP20, whereas BRET from the G5B-set1 mutant, which shows reduced interaction already in the basal state, was not affected by MP20 (Supplemental Figure 7C). Similar findings were observed for energy transfer betweenGPRC5C-wt and EP2-mVenus or GRPC5C-set1 mutant and EP2-mVenus (Supplemental Figure 7C). 

Having established MP20 as an inhibitor of GPRC5B/C-EP2 dimerization in receptor-overexpressing HEK cells, we next tested whether MP20 had comparable effects in mouse BMDMs. We found that MP20 dose-dependently reduced butaprost-induced cAMP production in control cells (Figure 6F), but less so in macrophages from M-G5b–KO mice, which, by nature, have reduced butaprost responses (Figure 6F). Further analyses showed that the minimal MP20 concentration required for significant inhibition of butaprost-induced cAMP production was 300 nM (Supplemental Figure 7D). MP20 did not affect BMDMs viability (Figure 6G), but increased phagocytosis in BMDMs from WT mice (Figure 6, H and I), but not in BMDMs from M-G5b-KOs (Supplemental Figure 7E). Furthermore, MP20 facilitated chemokine-induced migration (Figure 6J), and enhanced M1 polarization in M1-differentiated BMDMs while inhibiting M2 polarization in M2-differentiated BMDMs (Supplemental Figure 7, F and G). Also in RPMs, MP20 reduced butaprost-induced cAMP production (Figure 6K) and facilitated migration (Supplemental Figure 7H). Furthermore, we were able to show that MP20 disrupted coimmunoprecipitation of endogenous GPRC5B with endogenous EP2 in RPMs (Figure 6L). Importantly, MP20 inhibited butaprost-induced cAMP production also in human blood monocytes (Figure 6M), and it increased phagocytosis and inflammatory gene expression in human blood–derived macrophages (Figure 6N and Supplemental Figure 7, I–L, and Supplemental Figure 14). Taken together, MP20 inhibited GPRC5B/GPRC5C-mediated facilitation of EP2 signaling not only in HEK cells, but also in primary mouse and human macrophages.

### MP20 improves host defense both in bacterial peritonitis and influenza A virus infection.

**Figure 7:**
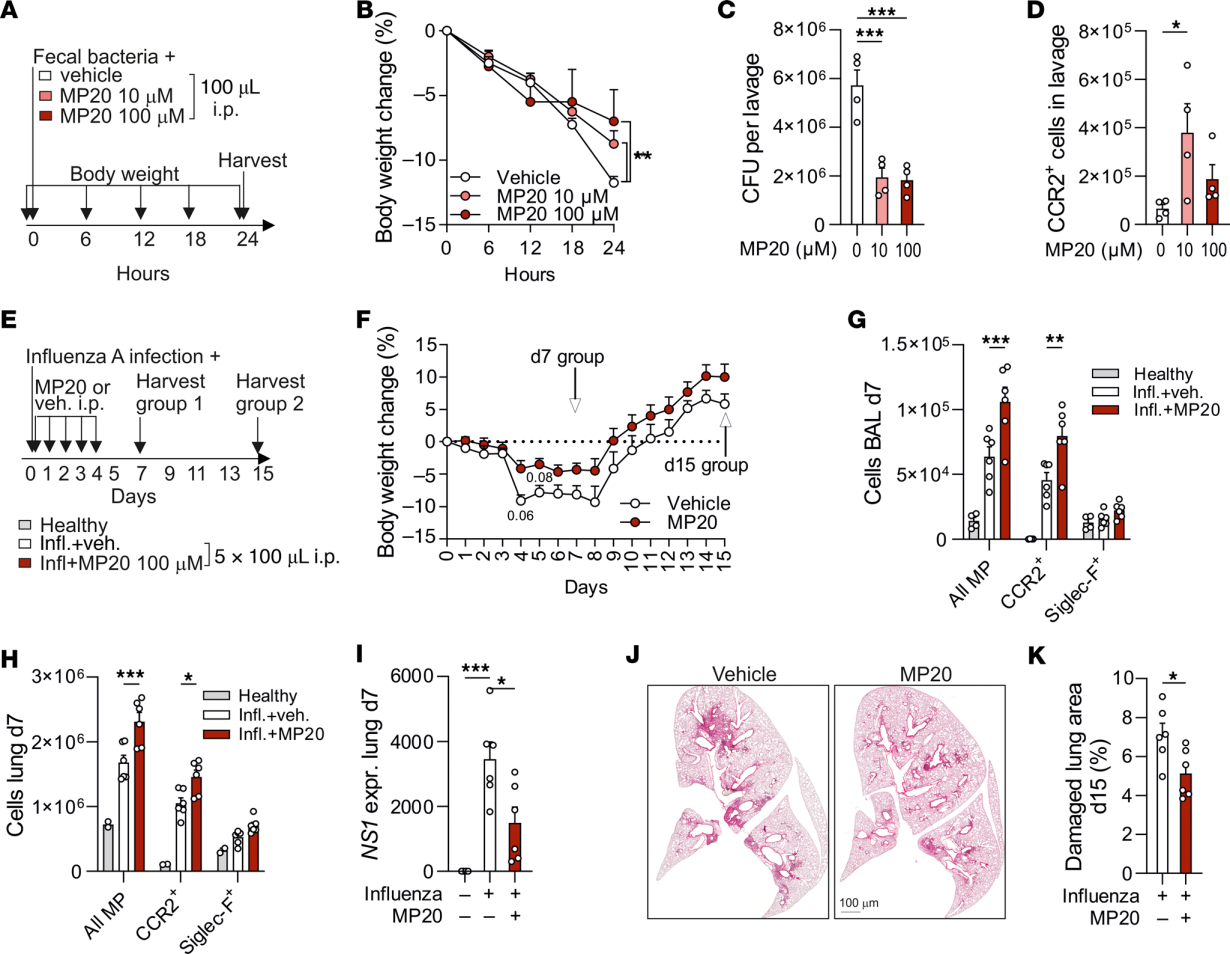
MP20 improves host defense both in bacterial peritonitis and influenza A virus infection. (**A**–**D**) Fecal peritonitis: (**A**) Experimental design. Fecal bacteria were coinjected with 100 μl vehicle or 100 μl of a 10 μM or 100 μM solution. (**B**–**D**) Effect of MP20 treatment on body weight change (**B**), number of bacterial CFU (**C**), and number of CD11b^+^, F4/80^lo^, MHCII^+^, CCR2^+^ macrophages (**D**) in peritoneal lavage fluid harvested 24 hour after injection of fecal bacteria (*n* = 4). (**E**–**K**) Influenza A virus infection: (**E**) experimental design. MP20 (100 μM) or vehicle were applied in a volume of 100 μl i.p. on days 0–4. (**F**) Effect of MP20 on body weight change after influenza infection. (**G**–**I**) Analysis d7: Flow cytometric analysis of macrophage populations in bronchioalveolar lavage (BAL, **G**) or digested lung tissue (**H**) (for gating strategy, please see Supplemental Materials); virus load was judged by qRT-PCR detection of viral NS1 (**I**) (*n* = 7). (**J** and **K**) Histological analysis of lung damage using H&E staining: exemplary images (**J**) and statistical evaluation of damaged area (**K**, *n* = 6). Data are means ± SEM; comparisons between treatments were performed using 2-way ANOVA with Dunnett’s (**B**) or Šídák’s (**F**–**H**) multiple comparisons test, 2-way ANOVA with Dunnett’s (**C** and **D**) or Tukey’s (**I**) multiple comparisons test, or unpaired, 2-sided *t* test (**K**). *n*, number of independent experiments or mice; *P < 0.05; **P < 0.01; ***P < 0.001.

To test whether MP20 is able to mimic the effect of GPRC5B/C knockout and GPRC5B/C decoy peptide in vivo, we applied MP20 intraperitoneally in the fecal peritonitis model (Figure 7A). Assuming that intraperitoneally injected MP20 is diluted approximately 10-fold in peritoneal fluid and inflammatory ascites, we used 10 × higher concentrations than in vitro, namely stock solutions of 10 μM and 100 μM, respectively. We found that both MP20 concentrations reduced body weight loss and improved bacterial clearance, and macrophage recruitment was increased (Figure 7, B–D). Interestingly, a beneficial effect of MP20 also existed in the presence of antibacterial treatment (Supplemental Figure 8, A–C), while it was abrogated in G5b-mut mice (Supplemental Figure 8, D–F). In healthy mice, in contrast, MP20 did not have clear effects on body weight or composition of peritoneal leukocytes (Supplemental Figure 8, G and H).

To investigate whether enhanced macrophage activity would also be beneficial in viral infection, we employed a mouse model of influenza A infection (20). After intratracheal instillation of influenza A virus, mice received i.p. injections of vehicle or MP20 from days 0–4 and virus load and leukocyte infiltration were determined on days 7 and 15 (Figure 7E). Mass spectrometric analysis showed that MP20 plasma levels peaked at 1 hour after i.p. application, reaching approximately 300 nM (Supplemental Figure 8I). On day 7, the peak of disease development, MP20-treated mice showed lower body weight loss compared with vehicle-treated mice (Figure 7F) and flow cytometric analysis of myeloid populations in bronchoalveolar lavage (BAL) and lung tissue showed increased macrophage numbers (Figure 7, G and H). This increase was mainly due to elevated numbers of CCR2-positive macrophages, whereas Siglec-F–positive alveolar macrophages, Ly6G-positive neutrophils, or B and T cells were not significantly changed (Figure 7, G and H, and Supplemental Figure 8, J and K). Increased macrophage recruitment in MP20-treated mice was accompanied by reduced virus load in infected lungs (Figure 7I). Increased monocyte and macrophage numbers were also observed on day 4 after infection, and a viral plaque forming assays confirmed reduced viral load (Supplemental Figure 9). In line with improved virus clearance, lungs of MP20-treated mice showed in the resolution phase, day 15, reduced histological sequalae of infection (Figure 7, J and K). Consistent with these findings, we also observed in G5b-mut mice reduced body weight loss, improved macrophage recruitment, and accelerated virus clearance after influenza infection (Supplemental Figure 10). Together, these data show that inhibition of GPRC5B/C dimerization by MP20 enhances macrophage activity and results in improved host defense both in bacterial and viral infection models.

### MP20 reduces smooth muscle contractility and protects from arterial hypertension.

**Figure 8:**
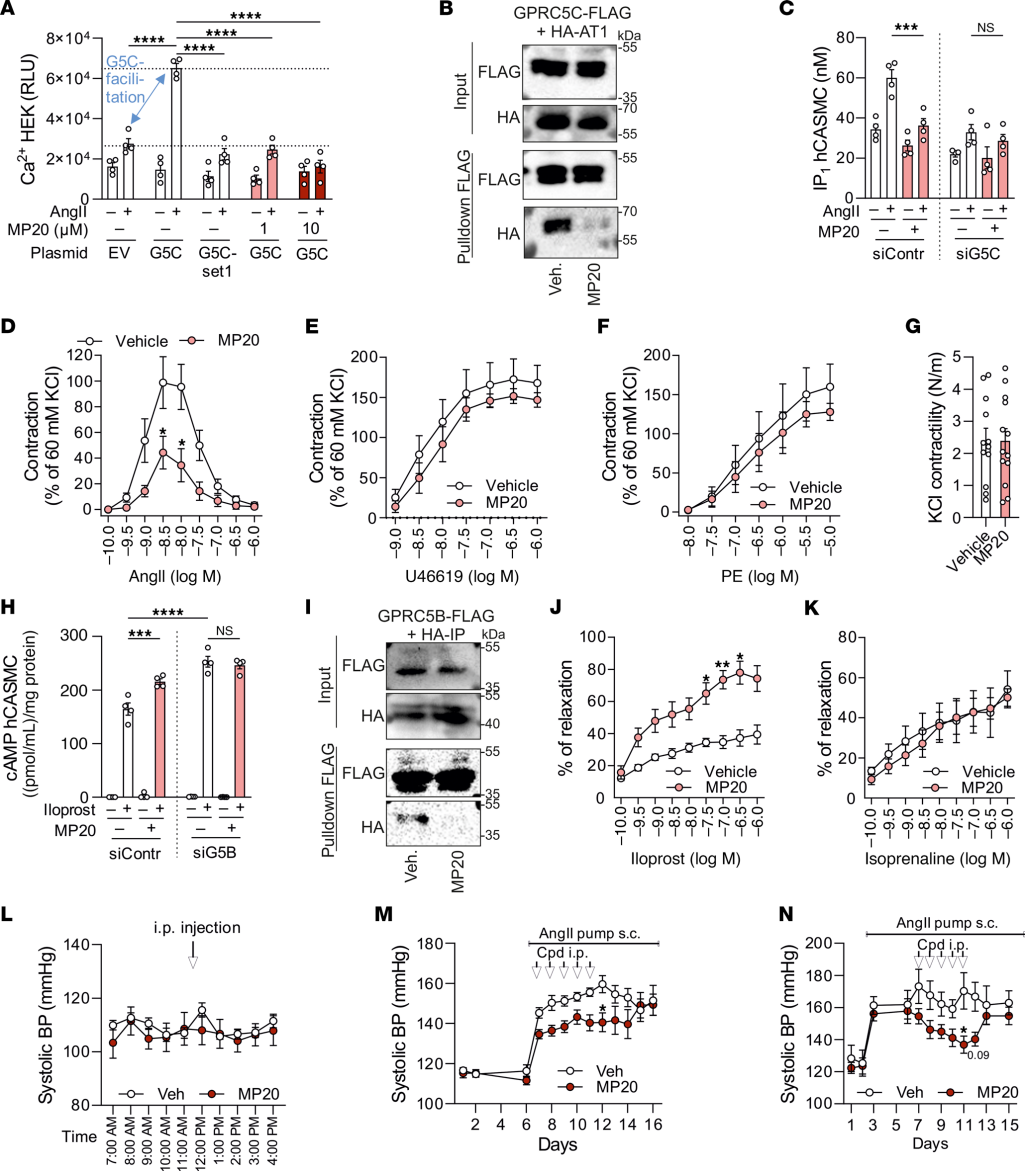
MP20 reduces SMC contractility and protects from arterial hypertension. (**A**) Effect of MP20 (1 hour) on AngII-induced calcium mobilization in HEK cells expressing calcium sensor aequorin and indicated plasmids (*n* = 4). (**B**) Coimmunoprecipitation of HA-AT1 with GPRC5C-FLAG in HEK cells treated with vehicle or MP20 (10 μM, 1 hour). (**C**) Effect of MP20 (1 μM) on AngII-induced IP_1_ production in hCASMCs transfected with control siRNA (siContr) or GPRC5C siRNA (siG5C) (*n* = 4). (**D**–**F**) Vasoconstrictor responses were determined by wire myography in mesenteric arteries preincubated with vehicle or MP20 (1 μM, 1 hour) (*n* = 9–10, data normalized to contraction elicited by 60 mM KCl). The bell-shaped AngII response reflects rapid receptor desensitization. (**G**) KCl-induced contraction in mesenteric arteries preincubated with vehicle or MP20 (1 μM, 1h) (*n* = 9–12). (**H**) Effect of MP20 (1 μM) on Iloprost-induced cAMP production in hCASMCs transfected with control siRNA (siContr) or GPRC5B siRNA (siG5B) (*n* = 4). (**I**) Coimmunoprecipitation of HA-IP with GPRC5B-FLAG in HEK cells treated with vehicle or MP20 (10 μM, 1 hour). (**J** and **K**) Iloprost- or isoprenaline-induced relaxation was determined in phenylephrine-precontracted mesenteric arteries that had been preincubated with vehicle or MP20 (1 μM, 1 hour) (*n* = 6). (**L**–**N**) Effect of vehicle or MP20 (100 μl i.p.,100 μM) on blood pressure: (**L**) Effect on basal blood pressure (*n* = 7–8); (**M**) Effect of 5 × application on hypertension development after implantation of AngII-releasing miniosmotic pumps (*n* = 13–14); (**N**), Effect of 5 × application in mice with established AngII hypertension (*n* = 5–6). Data are means ± SEM; comparisons between treatments were performed using 1-way ANOVA with Dunnett′s (**A**) or Tukey’s (**C** and **H**) post hoc test, 2-way ANOVA with Šídák’s post hoc test (**D**–**F** and **J**–**N**), or unpaired, 2-sided *t* test (**G**). *n*, number of independent experiments or mice; ns, not significant; *P < 0.05; **P < 0.01; ***P < 0.001; ****P < 0.0001. All experiments were performed in WT mice.

We finally investigated whether the inhibitory effects of MP20 were restricted to GPRC5B/C-EP2 dimerization or also affected other GPRC5B/C-containing oligomers. In SMCs, GPRC5C was shown to facilitate AT1 signaling, resulting in reduced AT1-induced calcium mobilization, IP_3_ production, and vascular contractility in the absence of GPRC5C (15). Also in HEK cells, overexpression of GPRC5C facilitated AngII-induced calcium mobilization, and this effect was not only abrogated when the G5C-set1 mutant was used, but also in the presence of MP20 (Figure 8A). Furthermore, HA-tagged AT1 receptor coimmunoprecipitated with GPRC5C-FLAG in HEK cells, and this effect was strongly reduced by MP20 (Figure 8B). In human coronary SMCs (hCASMCs), MP20 treatment did not affect viability (Supplemental Figure 11A), but it clearly reduced AngII-induced production of IP_1_, a stable metabolite of second messenger IP_3_ (Figure 8C). To test whether this effect was GPRC5C dependent, we performed the experiment in parallel in GPRC5C-knockdown hCASMCs (knockdown efficiency in Supplemental Figure 11B). As shown previously (15), AngII-induced IP_1_ production was reduced in GPRC5C knockdown SMCs, and MP20 did not exert further suppression (Figure 8C).

We next determined the functional consequences of reduced AT1 signaling in the presence of MP20. To do so, we studied agonist-induced contractility in isolated mouse mesenteric arteries using wire myography. Interestingly, vessels pretreated with MP20 for 1 hour showed clearly reduced AngII response (Figure 8D), whereas responses to other contractile stimuli such as thromboxane A2 agonist U46619, α1 adrenergic receptor agonist phenylephrine, or potassium chloride were not changed (Figure 8, E–G).

In addition to GPRC5C-dependent modulation of AT1 signaling, we previously showed that GPRC5B dimerizes in SMCs with the PGI_2_ receptor IP, resulting in GPRC5B-deficient SMCs in enhanced IP-dependent relaxation and protection from hypertension (13). We investigated whether MP20 was also reproducing this effect and found that cAMP production induced by IP receptor agonist Iloprost was significantly increased in the presence of MP20 (Figure 8H). To determine whether this effect was GPRC5B dependent, we performed parallel experiments in GPRC5B knockdown hCASMCs (knockdown efficiency in Supplemental Figure 11C). These cells showed the expected facilitation of IP signaling (13), but MP20 was unable to further enhance the Iloprost response (Figure 8H). In support of a disturbed GPRC5B/IP interaction, we observed reduced coimmunoprecipitation of HA-tagged IP with FLAG-tagged GPRC5B in MP20-treated HEK cells (Figure 8I). In wire myography, preincubation with MP20 clearly increased Iloprost relaxation in mesenteric arteries (Figure 8J), while effects of other relaxant factors such as β adrenergic agonist isoprenaline or sodium nitroprusside were not altered (Figure 8K and Supplemental Figure 11D).

To determine the in vivo consequences of reduced AT1-mediated contraction and increased IP-dependent relaxation, we implanted telemetric catheters and determined blood pressure levels in freely moving mice before and after i.p. MP20 application. In the basal state, MP20 did not induce obvious changes in systolic, mean, and diastolic blood pressure as well as heart rate (Figure 8L and Supplemental Figure 12, A–C). To induce arterial hypertension, mice were implanted with AngII-releasing miniosmotic pumps and the effect of vehicle or MP20 injection (1 × daily for 5 days) was determined. MP20-treated mice showed attenuated blood pressure increases compared with vehicle-treated mice, and this effect was maintained until 3 days after cessation of treatment (Figure 8M and Supplemental Figure 12, D–F). To also assess the potential therapeutic benefit in already established hypertension, we determined the effect of MP20 in mice with preexisting hypertension. Also here, MP20 induced a marked reduction of blood pressure (Figure 8N and Supplemental Figure 12, G–I). In line with a GPRC5B/GPRC5C-dependent mode of action, MP20 did not reduce arterial hypertension in mice with smooth muscle-specific GPRC5B/GPRC5C deficiency (Supplemental Figure 12J). Taken together, these data show that MP20 also inhibits the GPRC5B/IP and GPRC5C/AT1 interaction, resulting in SMCs in improved IP-dependent vasorelaxation and reduced AT1-dependent vasocontraction.

## Discussion

We show in this study that pharmacological interference with GPRC5B/C dimerization is possible and results in beneficial effects with respect to host defense and blood pressure control.

It has long been established that class C GPCRs such as metabotropic glutamate receptors, GABA_B_ receptors, or sweet and umami taste receptors are obligatory homo- or heterodimers (6, 7), but the role of class C orphan GPCRs as dimerization partners has only recently been recognized (13–15). Our previous in silico homology modelling and protein:protein docking studies identified 3 amino acids in GPRC5B transmembrane region 2 as potential interacting residues, and mutation of these residues abrogated coimmunoprecipitation and BRET between GPRC5B and EP2 in overexpressing HEK cells (14). We show that also in vivo, loss of the dimerization interface results in facilitation of innate immune responses, with consecutively enhanced host defense. This study therefore represents one of the rare cases of successful in silico GPCR heterodimer prediction, and, to the best of our knowledge, the first one involving a class C GPCR (21). It stands to reason that in this case, where no closely related GPCR with determined structure was available, our strategy of homology modelling and protein:protein docking — instead of directly predicting the dimer — was a factor for the successful identification of the interface.

Interestingly, coimmunoprecipitation experiments in HEK cells showed that GPRC5C has the same dimerization pattern as GPRC5B, whereas GPRC5A and GPRC5D differ strongly. In line with this, previous sequence alignments suggested that GPRC5 family members can be grouped in 2 subclusters: GPRC5B / GPRC5C are 50% identical and GPRC5A / GPRC5D are 52% identical; the sequence identities between the clusters vary between 37% and 41% (18). The amino acids making up the interaction interface in GPRC5B are largely conserved in GPRC5C, and our data show that their mutation prevents dimerization and generates hyperactivity in macrophages. In support of the notion that GPRC5B and GPRC5C play similar roles in the regulation of macrophage activity, GRPC5C-deficient macrophages phenocopy GPRC5B-KO macrophages. This functional redundancy does not necessarily apply to all cell types, since the expression patterns of GPRC5B and GPRC5C differ significantly (8). Furthermore, cellular effects independent of GPCR dimerization have been suggested; for example, the interaction of GPRC5B with sphingomyelin synthase 2 (22). However, in SMCs and macrophages, GPRC5B and GPRC5C seem to have largely comparable effects.

To explore the therapeutic potential of GPRC5B/C-mediated regulation of EP2 signaling, we investigated pharmacological strategies to interfere with the dimerization. Protein-protein interactions are notoriously difficult to target, and therapeutic approaches targeting GPCR heteromers are in their infancy (23). In vitro, peptide inhibitors mimicking the interface of 2 interacting GPCRs have been successfully used to interfere with homo- and heterodimerization (24–26). For example, both the β2-adrenergic receptor and chemokine receptor CXCR4 have been suggested to signal more efficiently as homodimers, and peptide inhibitors of interacting transmembrane regions were shown to impair both interaction and signaling (27, 28). Furthermore, cell-penetrating peptides were used to block the formation of GPCR heterodimers, for example between cannabinoid receptor CB1 and serotonin receptor 5-HT2A (29), or between AT1 angiotensin II receptor and the secretin receptor (30). However, most studies were performed in GPCR-overexpressing cell lines, and the functional relevance in primary cells or in vivo is unknown. An exception is the study of Borroto-Escuela et al. (31), which employed a synthetic peptide corresponding to the transmembrane 5 (TM5) region of the A_2_A receptor to disrupt the A_2_A–D_2_ heteroreceptor complex. When microinjected into the nucleus accumbens of rats, this TM5 peptide abolished the inhibitory effect of an A_2_AR agonist on cocaine self administration, effectively disrupting receptor-receptor interactions and restoring cocaine-seeking behavior (31). This provides direct evidence that interfering peptides targeting the dimer interface can modulate signaling and behavior. Our data show that cell-permeable peptides mimicking amino acids 95–106 of GPRC5B or 89–100 of GPRC5C are able to reduce co-IP of overexpressed receptors in HEK cells, thereby abrogating the facilitating effect on EP2 signaling. Furthermore, we show that this is also true in primary macrophages with endogenous receptor expression, where decoy peptides were able to mimic the hyperphagocytosis phenotype of GPRC5B-deficient and G5b-mut cells. These findings indicate that pharmacological interference with GPRC5B/EP2 dimerization is possible and may be exploited to modulate macrophage activation in vivo.

In contrast to peptide inhibitors of dimerization, few studies address the use of small molecules for this purpose. A 2021 study identified small-molecule allosteric modulators that act as enhancers or disruptors of rhodopsin oligomerization, including dimer interfaces (32). This provides proof of concept that small molecules can directly alter GPCR oligomeric state and downstream signaling in native cells. Furthermore, CXCR4 oligomerization is disrupted selectively by antagonists such as the cyclic peptide LY2510924 and the small molecule IT1t (33, 34). Both of these molecules bind to the intrahelical (orthosteric) binding site of CXCR4, however. In order to investigate whether a similar mechanism could be at play here, we docked MP20 to the equivalent intrahelical binding site of GPRC5B. As shown in Supplemental Figure 13, we found at least one pose that shows interactions of a similar quality as the pose in KS2 (Figure 5C). Hence, although we cannot completely rule out an alternative, CXCR4-like mechanism of dimer disruption, we still consider direct inhibition of the protein:protein interface as the more likely mode of action and consistent with the entirety of our experimental results. In any case, our study clearly shows that inhibition of orphan GPCR dimerization by small molecules is possible and leads to functionally relevant changes in the signaling of the affected protomers.

Regarding the therapeutic use of GPRC5B/C dimerization inhibitors, we showed that the enhanced activity of MP20-treated macrophages resulted in improved host defense in mouse models of bacterial and viral infection. Host-directed therapies that enhance macrophage function have been suggested and established for a number of infectious diseases, including tuberculosis (35), chronic granulomatous disease (36), and cryptococcal meningitis (37). In tuberculosis, agents such as statins, metformin, and imatinib are under investigation for their ability to enhance macrophage antimicrobial responses through autophagy and metabolic reprogramming (35, 38). Imiquimod, a TLR7 agonist, is used in HPV infection to stimulate innate immune cells (39), and trained-immunity strategies — like BCG revaccination or β-glucans — reprogram innate immune cells to enhance protection against pathogens such as influenza A virus (40). Together, these findings suggest that macrophage-enhancing therapies can augment standard antimicrobial and antiviral treatments, though broader clinical validation is needed. Importantly, we also found that in human monocytes and macrophages, MP20 is able to suppress butaprost-induced cAMP production and enhance phagocytosis. However, optimal timing of enhanced macrophage activity is likely to be crucial, since numerous studies suggested that hyperactive macrophages may also have detrimental effects. For example, excessive macrophage activation during lung infection can drive immunopathology by overproducing inflammatory cytokines, amplifying leukocyte recruitment, and causing acute lung injury/acute respiratory distress syndrome with impaired gas exchange (41, 42). Also, macrophage-derived mediators can induce epithelial apoptosis and promote fibrosis, leading to tissue damage and long-term loss of lung function (43). Furthermore, any therapies designed to bolster host defense may inadvertently disrupt immune balance and risk precipitating autoimmunity, as observed for immune checkpoint inhibitors or TLR7 agonists (39, 44). In line with this dual-edged nature of any immune-enhancing therapy, we did observe a trend to increased scores in EAE, though the differences were not significant. Further evaluation in other models will be required to determine the consequences of impaired GPRC5B dimerization for autoimmune processes.

As to the question of why macrophages rely on GPRC5B-dependent regulation of EP2 signaling, it is interesting to note that EP2 is — in contrast with other PGE_2_ receptors — not desensitized by high levels of PGE_2_ (45, 46). Since local PGE_2_ production is increased in acute inflammation (47), unchecked PGE_2_/EP2 signaling might hinder efficient macrophage activation. We previously showed that GPRC5B expression is downregulated in macrophages by inflammatory stimuli such as LPS (14), and we hypothesize that the resulting reduction of GPRC5B-mediated facilitation of antiinflammatory EP2 signaling contributes to efficient macrophage activation.

Interestingly, our data also suggest that GPRC5B/GPRC5C dimerization inhibitors may be beneficial in the prevention of arterial hypertension. Our data show that MP20 reduces AngII-induced hypertension in WT mice, thereby mimicking phenotypes previously observed in mice with smooth muscle–specific deficiency for GPRC5B (13) or GPRC5C (15). In further support of a GPRC5B/GPRC5C-dependent mode of action, MP20 failed to ameliorate AngII-induced hypertension in mice with smooth muscle–specific GPRC5B/C deficiency. However, whether dimerization inhibitors such as MP20 are suitable for therapy of arterial hypertension remains to be seen. Numerous highly efficient antihypertensive therapies targeting GPCRs are already in clinical use, such as antagonists at AT1 receptors or β-adrenergic receptors (48, 49). Also, IP agonists such as Iloprost are clinically used for the treatment of pulmonary hypertension (50). It seems unlikely that MP20 exceeds the therapeutic efficiency of these individual drugs, but its dual mode of action — combined inhibition of AT1 and facilitation of IP — is pharmacologically highly interesting. In addition, the relatively narrow spectrum of target cells might be beneficial; not many cell types coexpress GPRC5B/C and their interacting receptors, which reduces the risk of adverse effects in unrelated cell populations. Beyond macrophages and SMCs, GPRC5B and GPRC5C are highly expressed in pericytes and fibroblasts; in addition, GPRC5B is expressed in brain glia and endothelial cells. Since prostanoid and angiotensin receptors are known to modulate pericyte and fibroblast function (51, 52), it will be interesting to study consequences of MP20 in these cells. However, even though we showed that MP20 effects were largely absent in GPC5B/C-deficient cells, we currently cannot rule out that dimerization-independent effects also exist.

Taken together, our data show that inhibition of orphan GPCR dimerization by small molecules is feasible and improves infection control and arterial hypertension. However, our study has several limitations. First, a more comprehensive characterization of the pharmacokinetics of MP20 is needed to determine optimal dosing and administration regimen. Second, additional studies in human cell types are necessary to validate the translational relevance of our findings. Third, the effects of MP20 on other organ systems remain to be defined.

## Methods

### Sex as a biological variable

Both male and female mice and human participants were included in this study, with the exception of the EAE model, in which only female mice were used. Sex was not considered as a biological variable, and data from males and females were pooled for analysis.

### Reagents

The following reagents were used: Lipopolysaccharide (LPS, cat. L4391), human macrophage colony-stimulation factor (M-CSF, cat. SRP3110), N-Formyl-Met-Leu-Phe (fMLP, cat. F3506), isoprenaline (cat. I56270), phenylephrine (cat. P6126) from Sigma-Aldrich; murine M-CSF (cat. 315-02), murine IFN-γ (cat. 315-05), murine CCL5 (cat. 250-07), murine CCL2 (cat. 250-10), murine SDF-1β (cat. 250-20B), murine IL-4 (cat. 214-14) from PeproTech. 3-Isobutyl-1-methylxanthine (IBMX, cat. 2845) was from Bio-Techne GmbH. Luciferin D (cat. 122799) was purchased from PerkinElmer. Coelenterazine h (cat. S2001) was from Promega GmbH. Butaprost (cat. 13741), L-902,668 (cat. 10007712), angiotensin II (cat. 17150), and U46619 (cat. 16450) were from Cayman chemical company. Sodium nitroprusside (cat. 71780) was from Fluka. Complement component C5a (cat. 2150-C5-025) was from R&D systems. MP3 (cat. 029-893-349), MP6 (cat. 001-607-827), MP10 (cat. 000-248-785), MP11 (cat. 016-583-909), MP15 (cat. 042-577-353), MP19 (cat. 019-821-793), and K303MP20 (cat. 000-409-522) were from Molport. MP2 (cat. Z240993500), MP4 (cat. Z108818172), MP8 (cat. Z18544149), MP12 (cat. Z215312324), MP16 (cat. Z168984526), and MP17 (cat. Z148042584) were from Enamine.

Decoy peptides were synthesized at GenScript. Each peptide contained an N-terminal HIV-TAT sequence YGRKKRRQRRR (for cell permeability) to confer cell permeability, followed by target peptide LHFLFLLGTLGL (G5B decoy; its scramble control: FLTLLLLLFGHG) or TQVFFLLGTLGL (G5C decoy; its scramble control: QLLGFLTFGTLV). Both peptides were C-terminally amidated to increase stability. 

Information on expression vectors, antibodies, and primers can be found in the Supplemental Methods and Supplemental Tables 2–6.

### Experimental animals

#### Generation of G5b-mut mice.

mice carrying a mutated *Gprc5b* allele were generated by CRISPR/ Cas9 genome editing in murine zygotes as described (53). crRNAs targeting *Gprc5b* exon 1 upstream of the set1 sequence (upstream guide, 5′-AGTGAGACTACCCTTCATCA-3′) and downstream of the set 1 sequence (downstream guide, 5′-ATGGGCTCGTAGGCGCAGGC-3′) were designed using https://eu.idtdna.com/site/order/designtool/index/CRISPR_CUSTOM and VectorBuilder, respectively.

To provide the homology-directed repair DNA, an AAV1 vector carrying the 314 bp long mutated region between the two PAM sites was designed (“E1-mut”) and flanked with a 506 bp 5′ homology arm and a 460 bp 3′ homology arm. The “E1-mut” repair DNA sequence was: 5′-ACAAGGAAAGGAAGCGGCCTGTGTGCCTCCATGCCCTCTTCCTGGCCGGGACCGCCGGCCTCTTTGGCCTGACGTTTGCCTTCATCATCCAGATGGACGAGACAATCTGCTCCATCCGACGCTTCCTCTGGGGTGTCCTCTTCGCGCTCTGCTTTTCCTGCCTGCTGAGCCAGGCGTGGCGGGTGCGGAGGCTGGTGCGCCAGGGCACAAGCCCGGCCAGCTGGCAGCTGGTGAGCCTGGCACTGTGCCTGATGCTGGTGCAGGTCATCATTGCCACTGAGTGGCTGGTGCTGACTGTGCTGCGTGACACGAAG-3′ (F97->A, L101 ->A, L104->A). For genome editing, the Alt-R CRISPR-Cas9 system (Integrated DNA Technologies) was employed. Briefly, embryos were incubated with AAV1 (2 experimental groups with 1 × 10^8^ and 1 × 10^9^ viral genome copies (GC)/μl, respectively) for 6 hours, followed by electroporation with assembled Cas9/crRNA ribonucleoproteins (RNPs). RNPs were generated by incubating Cas9 nuclease (40 ng/μl; IDT, 1081058) with pre-annealed crRNA:tracrRNA duplexes (100 ng/μl; 1:1 ratio, IDT, 107232). This resulted in successful replacement of the set 1 sequence in 2 homozygous and 9 heterozygous mice (1 × 10^8^ GC/μl: 2 homozygous, 1 heterozygous, 10 random insertions/deletions; 1 × 10^9^ GC/μl: 8 heterozygous, 7 random insertions/deletions, 4 WT). Homozygous and selected heterozygous animals were subsequently backcrossed to C57BL/6J to establish the G5b-mut line. Genotyping of wild-type *Gprc5b* was performed using primers 5′- TTCCTCTTCCTGCTGGGG-3′ and 5′- CAGGCATCTCCCTGCTTAAT-3′, which resulted in a band size of 512 bp. The mutant allele was detected with primers 5′- TGCCCTCTTCCTGGCCG-3′ and 5′-CCAGGCATCTCCCTGCTTAAT -3′, which resulted in a band size of 514 bp. To confirm presence of the mutated allele, PCR amplification was performed with primers 5′- GTTCCTGGTGTTAGAGAGAAAGA-3′ and 5′-GAAGGTAGTGACGAGGATGAAG-3′ (amplicon size: 712 bp), followed by Sanger sequencing using primer 5′-ACTGGACCTTCTTCCTCAGTA-3′.

#### Generation of M-G5b-KOs.

*Gprc5b*^fl/fl^ mice (13) were intercrossed with LysM-Cre mice (B6.129P2-Lyz2tm1(cre)Ifo/J) (19) to generate myeloid cell-specific GPRC5B-deficient mice (M-G5b-KOs). Mice were maintained on a C57BL/6J background and if not otherwise indicated, genetically matched Cre-negative *Gprc5b*^fl/fl^ mice were used as controls. Genotyping of *Gprc5b* alleles was done with primers 5′-GCTGGAAGGTTTCTCCCTCT-3′ and 5′-AAGAGACAACCACCAGACAGG-3′, resulting in band sizes of 361 bp for the wild-type allele and 478 bp for the floxed allele. Genotyping for *LysM*-Cre was done with primers 5′-CTTGGGCTGCCAGAATTTCTC -3′ and 5′-CCCAGAAATGAATTACG -3′, resulting in a band size of 834 bp.

#### Generation of M-G5c-KO.

*Gprc5c^fl/fl^* mice (15) were intercrossed with *LysM*-Cre mice (B6.129P2-Lyz2tm1(cre)Ifo/J) (19) to generate myeloid cell-specific GPRC5C-deficient mice (M-G5c-KO). Mice were maintained on a C57BL/6J background and if not otherwise indicated, genetically matched Cre-negative *Gprc5c*^fl/fl^ mice were used as controls. Genotyping of *Gprc5c* alleles was done with primers 5′- GCTGAGAAACGAATCCTCAACTA-3′ and 5′-GCTGAACCACAGCCTGTAAG -3′, resulting in band sizes of 253 bp for the WT allele and 293 bp for the floxed allele.

#### Generation of iSM-G5b/c-KO.

Gprc5b(fl/fl) (13) mice, Gprc5c(fl/fl) (15) mice, and *Myh11*-CreERT2 (54) mice were intercrossed to generate tamoxifen-inducible, smooth muscle-specific GPRC5B/C knockout mice (iSM-G5b/c-KOs). Mice were maintained on a C57BL/6J background. Genotyping of Gprc5b alleles was done with primers 5′-GCTGGAAGGTTTCTCCCTCT-3′ and 5′-AAGAGACAACCACCAGACAGG-3′, resulting in band sizes of 361 bp for the wild-type allele and 478 bp for the floxed allele. Genotyping of Gprc5c alleles was done with primers 5′- GCTGAGAAACGAATCCTCAACTA-3′ and 5′-GCTGAACCACAGCCTGTAAG -3′, resulting in band sizes of 253 bp for the wild-type allele and 293 bp for the floxed allele.

Mice were kept in individually ventilated microisolator cages with 12-hour light-dark cycles in a specific pathogen-free facility with a temperatures range between 20–24°C and humidity between 45–65%. Standard chow (Altromin GmbH Lage, Altromin 1320), tap water, and nesting material were provided ad libitum. Mice were analyzed at an age of 6–16 weeks and both female and male mice were used. Mice were euthanized under CO_2_ anaesthesia or by intraperitoneal injection of ketamine/xylazine.

### Animal models

Bacterial peritonitis was induced by intraperitoneal injection of fecal suspensions as described previously (13). Briefly, fresh stool was collected and dissolved in Dulbecco’s Phosphate-Buffered Saline (PBS, Gibco, 14040141) at a concentration of 30 mg/ml. The stool suspension was filtered through a 70 μm cell strainer and 100 μl of the resulting suspension were injected intraperitoneally into mice. Thereafter, mice were observed and scored every 1.5 hours up to 24 hours. Body weight was measured every 6 hours up to 24 hours. 24 hours after of peritonitis induction, mice were sacrificed under CO2 and peritoneal lavage was performed using 7 ml of PBS containing 0.5% BSA (Serva, 11930) and 2 mM EDTA (Roth, 8043). In some cases, decoy/control peptides (100 μl, 100 μM) or MP20/vehicle (100 μl, 10 or 100 μM) were co-injected with fecal bacteria. Lavage fluid was used for FACS analysis of leukocyte populations or determination of bacterial clearance. For the latter, lavage fluid was serially diluted and plated on LB agar plates. After 16 hours of incubation, numbers of colony-forming units were determined. To evaluate the synergistic effect of MP20 and antibacterial treatment, MP20 (100 μM) was injected intraperitoneally together with bacterial suspension, and amoxicillin/clavulanic acid (1A Pharma) (2 mg/kg in saline) or control solution (saline) was administered subcutaneously 2 hours later.

Experimental autoimmune encephalomyelitis (EAE) was induced as described previously. Briefly, total 200 μg of MOG_35-55_ myelin oligodendrocyte glycoprotein peptide emulsified in complete Freund’s adjuvant (Hooke Laboratory, EK-2110) were administered subcutaneously at 2 sites of female mice. On day 0 and 1, mice were treated with 110 ng of pertussis toxin in 100 μl of PBS intraperitoneally. The clinical score was assessed to evaluate the progression of neuroinflammation and was conducted as follows: 0, no clinical disease; 0.5, reduced tail tone; 1, limp tail; 1.5, lack of reflex compensatory movements when walking; 2, gait ataxia; 2.5, slight paralysis of the hindlegs; 3, plegia of one leg or moderate paralysis of both legs; 3.5, paraplegia with complete paralysis of both hindlegs; 4, tetraparesis with paralysis of the front extremities; 4.5 moribund; 5, dead.

To measure blood pressure, a radiotelemetry system (Data Sciences International, PA-C10) was implanted into the left common carotid artery under anaesthesia with ketamine (120 mg/kg) and xylazine (16 mg/kg). Intraoperative and postoperative analgesia was provided with metamizole (0.8 ml/500 ml; via drinking water) and buprenorphine (0.05–0.1 mg/ml, subcutaneous). To induce AngII-mediated hypertension, osmotic pumps (Alzet, model 2004) releasing AngII (2000 ng/kg per min) for 28 days were implanted subcutaneously under isoflurane anaesthesia, with intraoperative and postoperative analgesia using metamizole (via drinking water). Blood pressure and heart rate were recorded and analyzed with Ponemah software (DSI, version 6.40.20119.1). Vehicle or MP20 (100 μl, 100 μM) were injected daily for 5 consecutive days, starting either on the day of AngII pump implantation or after AngII-induced hypertension had been established.

For infection with influenza A virus, mice were anesthetized with isoflurane and intra-tracheally inoculated with 2000 plaque-forming units of influenza A virus PR8 (A/PR/8/1934, H1N1) diluted in 70 μl sterile PBS. For compound treatment, mice received intraperitoneal injection of compound (100 μM in 100 μl PBS) or vehicle once daily from day 0 for 5 consecutive days. Mice were monitored daily. At 4 days post virus infection, mice were sacrificed using ketamine (180 mg/kg) and xylazine (16 mg/kg) in 0.9% NaCl. Bronchoalveolar lavage was performed three times with 1 ml PBS via an 18G catheter, and the lavage fluid was used for viral plaque assay. Subsequently, lungs were harvested after PBS perfusion and used for FACS analysis of leukocyte populations. At 7 days post viral infection, mice were euthanized as described above. Bronchoalveolar lavage was performed as described above, and the collected lavage fluid were used for FACS analysis of leukocyte populations. Lungs were harvested following PBS perfusion and used for FACS analysis of leukocyte populations or for qPCR to assess viral clearance. At 15 days post virus infection, mice were sacrificed using the same ketamine/xylazine protocol. Following PBS perfusion, a tight surgical knot was placed around the left main airway, and the left lung lobe was excised for FACS analysis of leukocyte populations. The right lung lobes were inflated with 4% PFA for 5 minutes and processed for histological analysis.

To measure the plasma levels of MP20, mice were intraperitoneally injected with MP20 (100 μM in 100 μl of PBS). At 0, 20 minutes, 60 minutes, 120 minutes, 12 hours, and 24 hours, mice were sacrificed under CO_2_ and blood was collected via cardiac puncture into EDTA coated tubes (SARSTEDT, 16.444) and kept on ice. To obtain plasma, blood samples were centrifuged at 2,000 × *g* at 4°C for 10 minutes, and the supernatant was collected and stored at –80°C until analysis.

### Compound screening by in silico docking

DOCK3.7 (55) was chosen as the docking software. Matching spheres were generated using dummy atoms to define the binding site. The subsets of ZINC20 (56) lead-like and drug-like were used as virtual libraries for the docking screening. Poses were clustered using Daylight fingerprints and Tanimoto similarity as implemented in the Butina Clustering method (57), and the top cluster representatives were extracted for each docking, for a total of 5390. The extracted molecules were then inspected visually, and those with unfavorable interactions were discarded. The compounds’ conformation library was generated in-house in the db2 format suitable for DOCK for the docking calculations of analogs of original ZINC compounds. The top-scored poses of each calculation were then clustered based on Tanimoto similarity, and cluster representatives were visually inspected. For details, see Supplemental Methods.

### Statistics

All data are presented as means ± SEM. Data were tested for normal distribution using Shapiro-Wilk test; statistical tests are indicated in the figure legends. Statistical analyses were performed using 2-sided unpaired *t* tests for comparisons between 2 groups, 1-way ANOVA followed by Dunnett’s or Tukey’s multiple-comparisons test for single-factor analyses, and 2-way ANOVA followed by Dunnett’s, Tukey’s, or Šidák’s multiple-comparisons test for multifactorial analyses. “*n*” refers to the number of independent experiments or mice per group. A *P* value less than 0.05 was considered significant. Exemplary images and blots were selected to represent the average of the respective cohort. All statistical analyses were performed using GraphPad Prism 10 (version 10.1.2).

All other methods are described in the Supplemental Methods (pages 28–39 of Supplemental Materials).

### Study approval

All animal experiments were approved by the Institutional Animal Care and Use Committee of the *Regierungspräsidium* Darmstadt and in accord with Directive 2010/63/EU of the European Parliament on the protection of animals used for scientific purposes. Analyses were performed by investigators blinded to genotype and treatment. Experiments with human blood samples were performed according to the regulations of the local ethics committee of the Hessian Regional Medical Board (*Ethikkommission des Fachbereiches Medizin der Goethe-Universität Frankfurt*; AZ 110/11), and informed consent was obtained from all participants.

### Data availability

All data are available in the article and its Supplemental files or from the corresponding author upon request. a Supporting Data Values file is provided with this paper.

## Author contributions

JK performed most experiments and wrote parts of the manuscript. She was supported by J Shao (telemetry), BS and IS (Influenza A model), and MS (Mass spectrometry). J Shamsara, MP, and PK generated homology models and performed virtual docking analyses. PK and SO interpreted data and reviewed the manuscript. NW designed and supervised the study, analyzed data, and wrote the manuscript.

## Conflict of interest

The authors have declared that no conflict of interest exists.

## Funding support

- The Federal State of Hessen through the LOEWE-GLUE initiative LOEWE/2/12/519/03/05.001(0014)/71 (to PK, NW).
- The Deutsche Forschungsgemeinschaft (DFG, German Research Foundation) under Germany´s Excellence Strategy – EXC 2026, Cardio-Pulmonary Institute, Project ID: 390649896 (to MS, SO, NW).

## Supplementary Material

Supplemental data

Supplemental data set 2

Supplemental data set 3

Supplemental data set 4

Unedited blot and gel images

Supporting data values

## Version 1 08/03/2026

Electronic publication

## Version 2 08/07/2026

Fixed resolution in Figure 5
