# Supplementary material for: Small molecule inhibitor of orphan GPCR dimerization improves host defense and blood pressure control in mice
Source: J Clin Invest. 2026 Aug 3;136(15):e203162. doi: 10.1172/JCI203162 (PMC13430022; doi:10.1172/JCI203162)
Supplement: Supplemental data [file jci-136-203162-s098.pdf]

Supplemental Materials for Kwon et al., 2026:

Small molecule inhibitor of orphan GPCR dimerization improves host defense and blood pressure control in mice

1. **Supplemental Figures 1-15 (p. 2-21)**
2. **Supplemental Table 1-6 (p. 22-27)**
3. **Supplemental Methods (p. 28-39)**
4. **FACS Gating Strategies (p. 40-41)**
5. **Supplemental References (p. 42-43)**

1. Supplemental Figures for Kwon et al., 2026

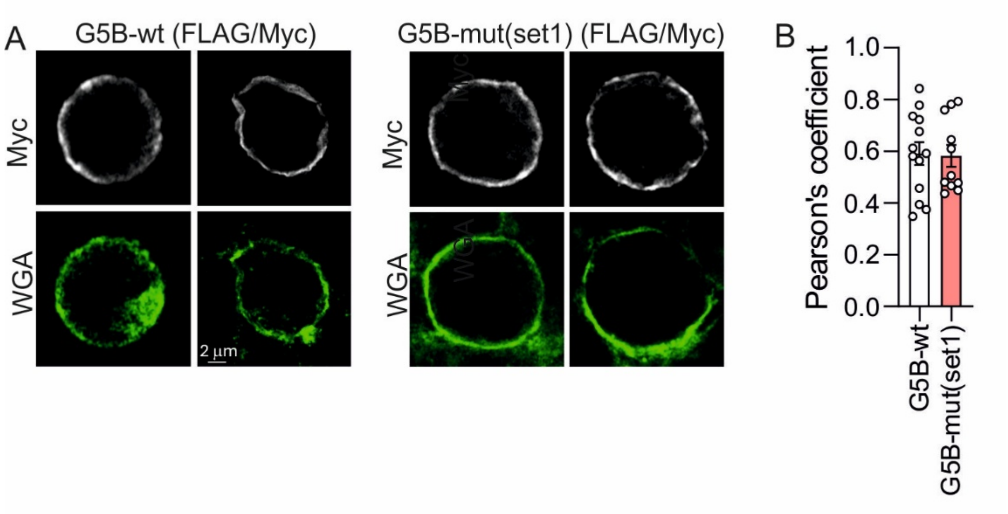

Suppl. Fig. 1: Intracellular distribution of FLAG/Myc-tagged wild-type GPRC5B (G5B-wt) and GPRC5B carrying alanine mutations F97A, L101A, L104A (G5B-mut(set1)) in HEK cells: immunofluorescence staining of Myc signals in permeabilized HEK cells with wheat germ agglutinin (WGA) as membrane staining. Exemplary photomicrographs (A) and statistical evaluation of Pearsons's coefficient for the colocalization of G5B variants and WGA (B, n=13 and 11 cells)**.** Data are means ± SEM; comparisons were done using unpaired, two-sided t test.

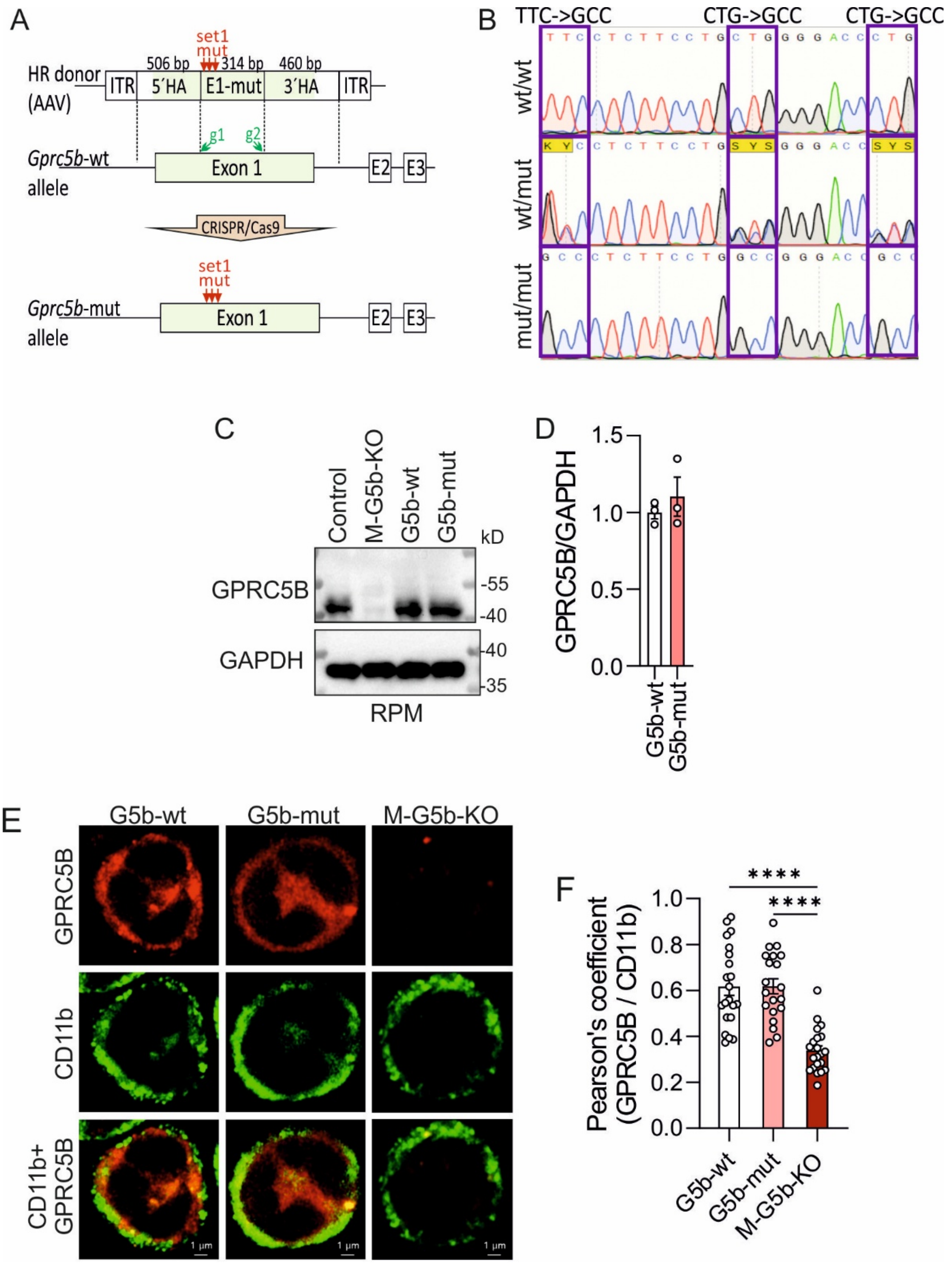

Suppl. Fig. 2: Generation and characterization of mice expressing a dimerization-deficient GPRC5B mutant (G5b-mut). (A,B) Generation of G5b-mut mice by CRISPR/Cas9 genome editing: **(A)** Schematic overview showing the adeno-associated virus (AAV) carrying the homology-directed repair DNA (HR donor (AAV)) as well as *Gprc5b* alleles before (*Gprc5b*-wt) and after (*Gprc5b*-mut) successful integration of the donor DNA. g1 and g2 indicate guide RNAs. **(B)** DNA sequencing in three exemplary mice generated by the targeting (in total 34 pups; among them 2 *Gprc5b*-mut/mut, 9 *Gprc5b*-wt/mut, 23 *Gprc5b*-wt/wt or random insertion/deletion). **(C)** Western

blot detection of GPRC5B signals in RPMs from G5b-wt and G5b-mut mice; RPMs from control mice and M-G5b-KOs as reference and specificity control; GAPDH as loading control. (**D)** Quantification of GPRC5B/GAPDH signal ratios in RPMs from G5b-wt and G5b-mut mice (n=3). **(E,F)** The localization of endogenous GPRC5B was determined by immunofluorescence staining in RPMs harvested from G5b-wt or G5b-mut mice (M-G5b-KOs as antibody specificity control; CD11b for membrane staining): E**,** representative photomicrographs; F, statistical evaluation of Pearsons's coefficient for the colocalization of GPRC5B and CD11b (n=20-21 cells)**.**

Data are means ± SEM; comparisons between groups were performed using unpaired, two-sided t test (D) or one-way ANOVA with Tukey's post hoc test (F). n, number of independent experiments or mice; ****, $P < 0.0001$.

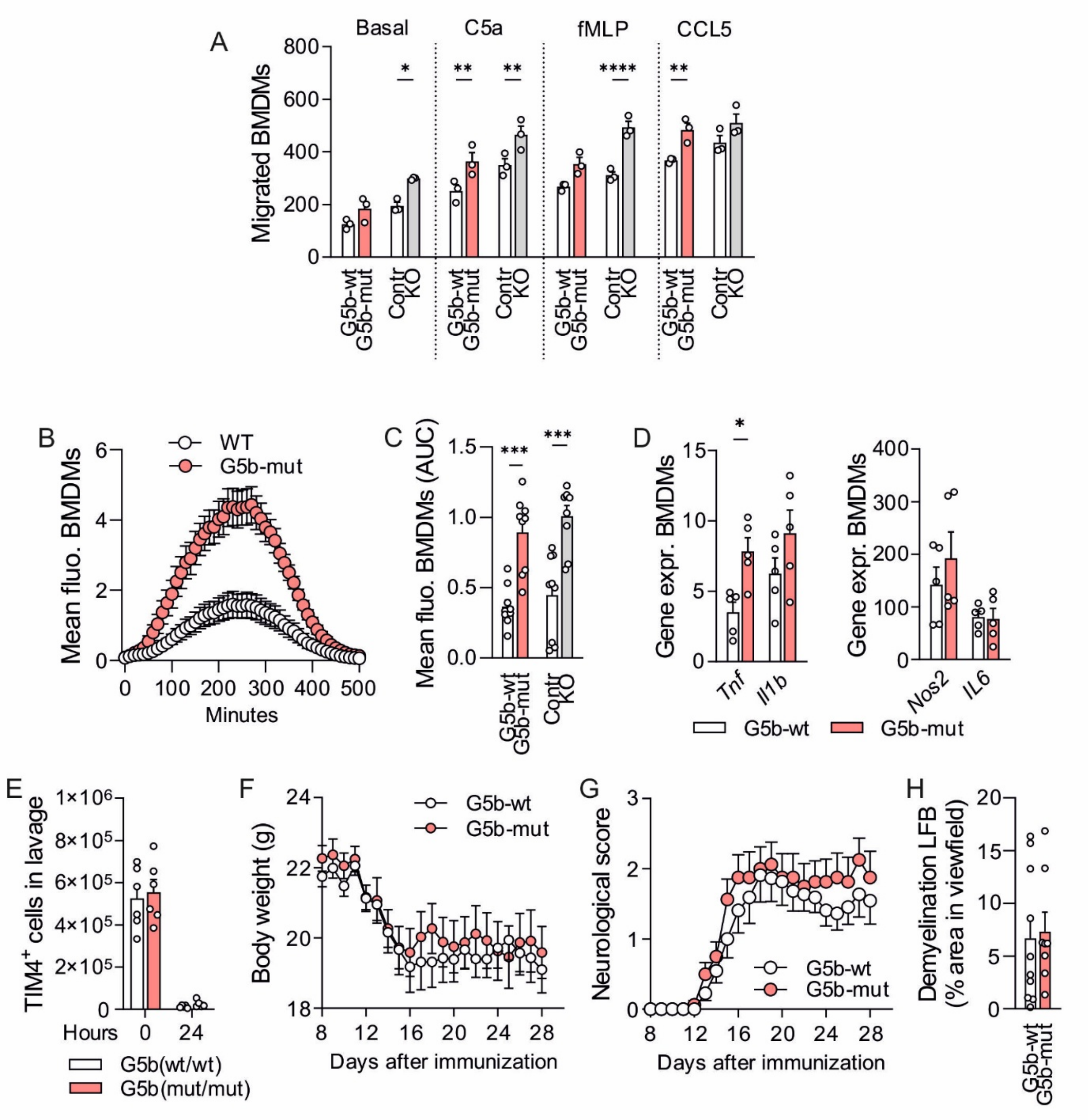

Suppl. Fig. 3: Enhanced macrophage activity in G5b-mut mice. (A) Transwell migration of G5b-wt and G5b-mut BMDMs in response to chemotactic factors; cells from control (Contr) and M-G5b-KOs (KO) as reference (n=3). **(B,C)** Phagocytosis of *E. coli* bioparticles in G5b-wt and G5b-mut BMDMs: Exemplary traces (B) and statistical evaluation of AUC (C); cells from control and G5b-KOs as reference (n=9). Please note that phagocytosis is generally higher in BMDMs than in naïve RPMs (compare to main text Fig. 1E), most likely due to their M1-skewed differentiation state.**(1, 2) (D)** The expression of pro-inflammatory genes was determined by qRT-PCR in BMDMs from G5b-wt and G5b-mut mice that were challenged with *E. coli* pHrodo bioparticles for 3 hours. Data were normalized to *Gapdh* and are shown as fold change compared to unchallenged BMDM (n=5). **(E)** Numbers of $CD11b^{+}$, $F4/80^{+}$, $MHCII^{-}$, $TIM4^{+}$ RPMs before and 24 h after injection of fecal

bacteria (n=5-6). **(F-H)** Experimental autoimmune encephalomyelitis in G5b-wt and G5b-mut mice: body weight loss (F) and neurological score (G) was determined on days 8-28 after immunization with MOG_{35-55} peptide; the degree of demyelination was analyzed by luxol fast blue staining on day 28 (H) (n=10 and 8).

Data are means ± SEM; comparisons between treatment groups were performed using two-way ANOVA with Tukey's (A) or Sidak's (D, E, F, G) multiple comparisons test, or unpaired, two-sided t tests (C,H). n, number of independent experiments or mice; *, $P < 0.05$; **, $P < 0.01$; ***, $P < 0.001$; ****, $P < 0.0001$.

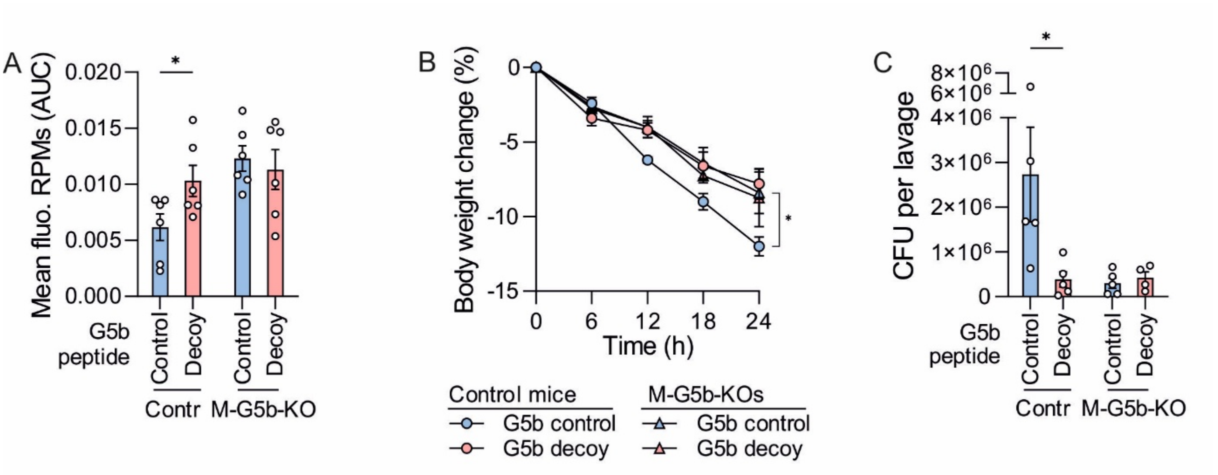

Suppl. Fig. 4: A, Peptide effect (1 µM each) on phagocytosis of pHrodo *E. coli* bioparticles in RPMs from control mice and M-G5b-KOs (n=5). **B,C,** MP20 effect (100 µl of a 100 µM stock) on fecal peritonitis in G5b-wt mice and G5b-mut mice: Effect of MP20 treatment on body weight change (B) and number of bacterial CFU in peritoneal lavage fluid harvested 24 h after injection of fecal bacteria (C) (n=5).

Data are means ± SEM; comparisons between treatments were performed using unpaired t tests (A) or two-way ANOVA with Dunnett's (B) or Sidak's (C) post hoc test. *, $P < 0.05$.

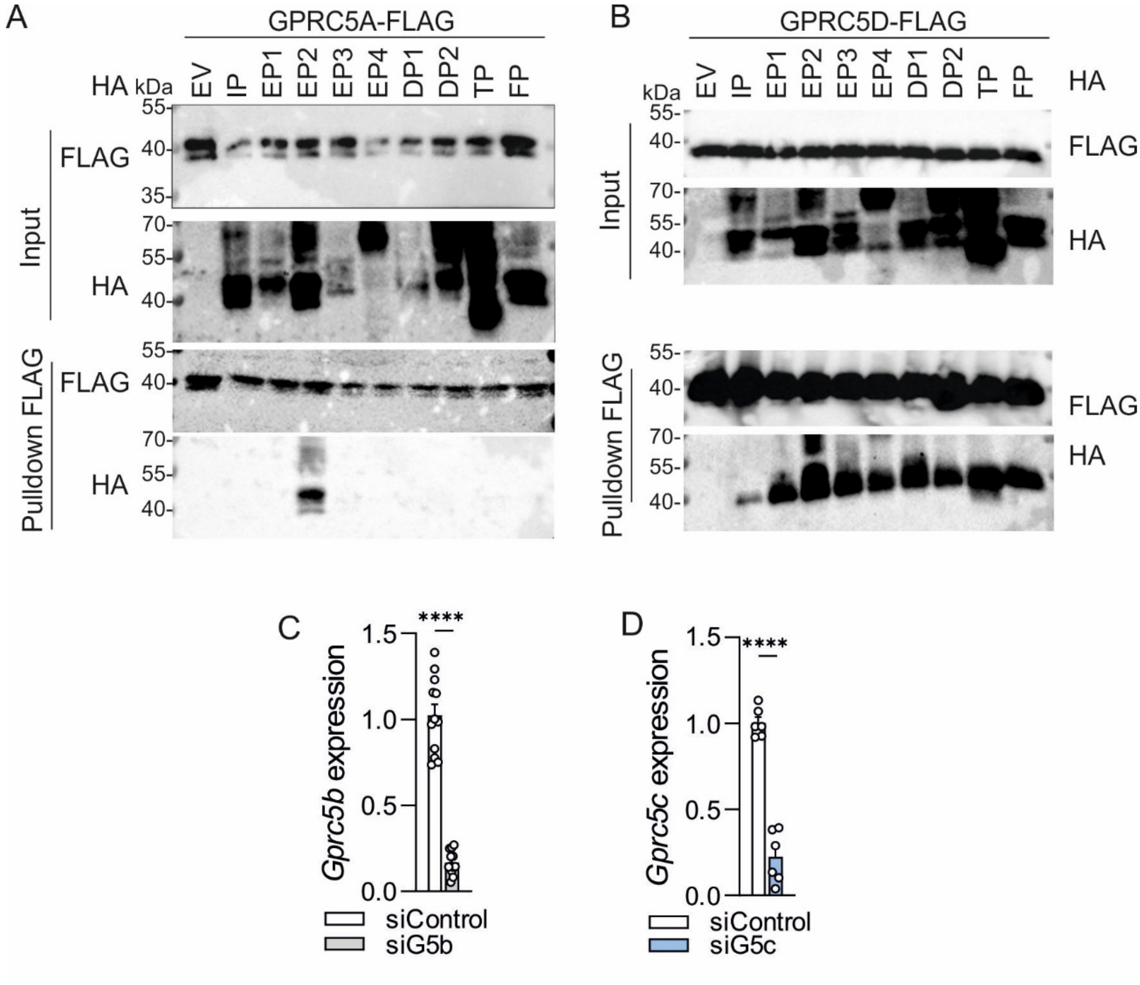

Suppl. Fig. 5: GPRC5C resembles GPRC5B with respect to dimerization pattern, effect on cellular cAMP, and macrophage activation. (A,B) Western blot detection of HA and FLAG signals in lysates of HEK cells expressing FLAG-tagged GPRC5A (A) or GPRC5D (B) in combination with different HA-tagged prostanoid receptors before ("input") and after immunoprecipitation of GPRC5B-FLAG ("Pulldown FLAG"). (**C,D)** Knockdown efficiency in HEK cells transfected with control siRNA (siControl) or siRNA directed against *Gprc5b* (siG5b, C, n=12) or *Gprc5c* (siG5c, D, n=6). Data are normalized to *Gapdh*, control set to 1.

Data are means ± SEM; comparisons between genotypes were performed using unpaired, two-sided t test (C,D). n, number of independent experiments; ****, $P < 0.0001$.

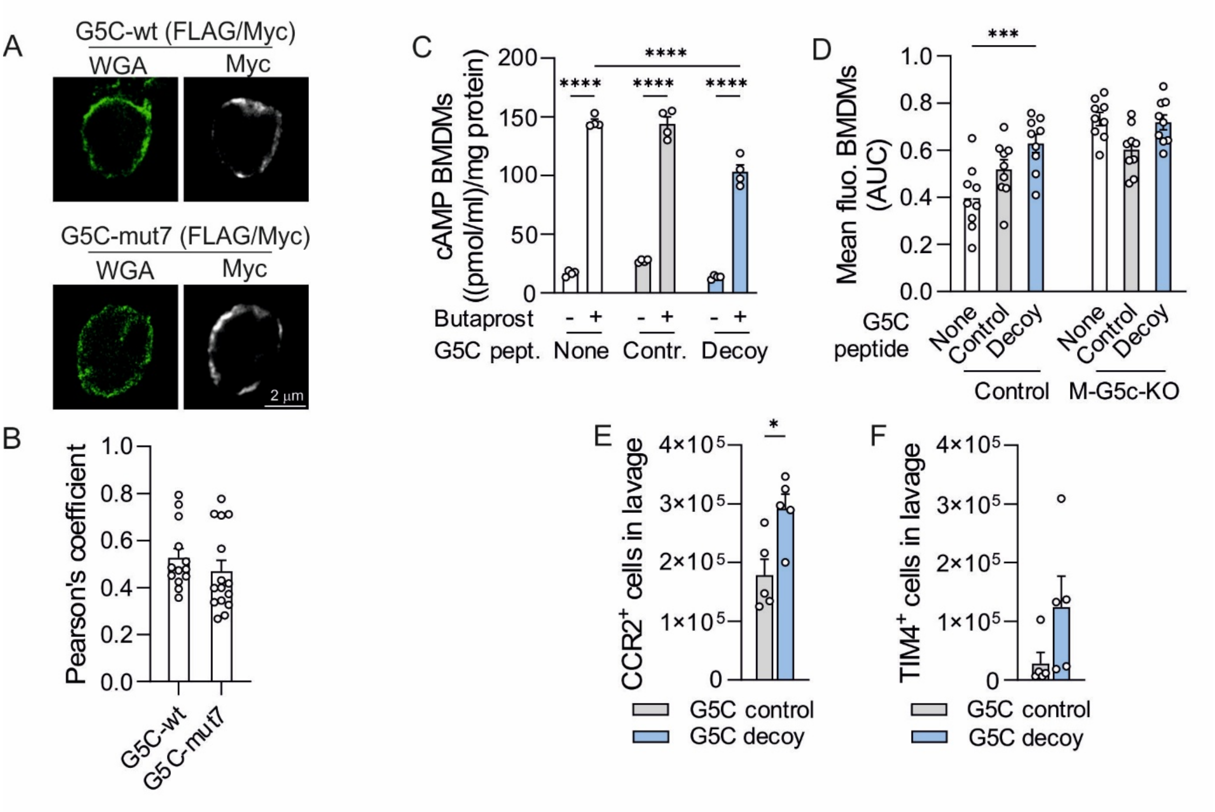

Suppl. Fig. 6: GPRC5C-mediated effects on cAMP production and macrophage activity are blocked by interface mutation and decoy peptide. (A,B) Intracellular distribution of FLAG/Myc-tagged wild-type GPRC5C (G5C-wt) and GPRC5C carrying 7 alanine mutations (G5C-mut7) in permeabilized HEK cells (wheat germ agglutinin (WGA) as membrane staining): Exemplary photomicrographs (A) and statistical evaluation of Pearsons's coefficient for the colocalization of G5C variants and WGA (B, n=13-15 cells). **(C)** Effect of GPRC5C decoy peptide and control peptide (1 µM each) on butaprost-induced cAMP production in M0 BMDMs (n=4). **(D)** Peptide effect on phagocytosis in M0 BMDMs from wild-type mice (left) and M-G5c-KO mice (right) (n=9). **(E,F)** Fecal peritonitis in G5b decoy/control peptide-treated mice: Effect of peptides on numbers of CD11b$^+$, F4/80^{lo}, MHCII$^+$, CCR2$^+$ macrophages (E) and CD11b$^+$, F4/80$^+$, MHCII$^-$, TIM4$^+$ RPMs (F) in peritoneal lavage fluid harvested 24h after injection of fecal bacteria (n=5).

Data are means ± SEM; comparisons between treatments were performed using one-way ANOVA with Tukey's multiple comparisons test (C,D) or unpaired, two-sided t test (E,F). n, number of independent experiments or mice; ns, not significant; *, $P < 0.05$; **, $P < 0.01$; ****, $P < 0.0001$.

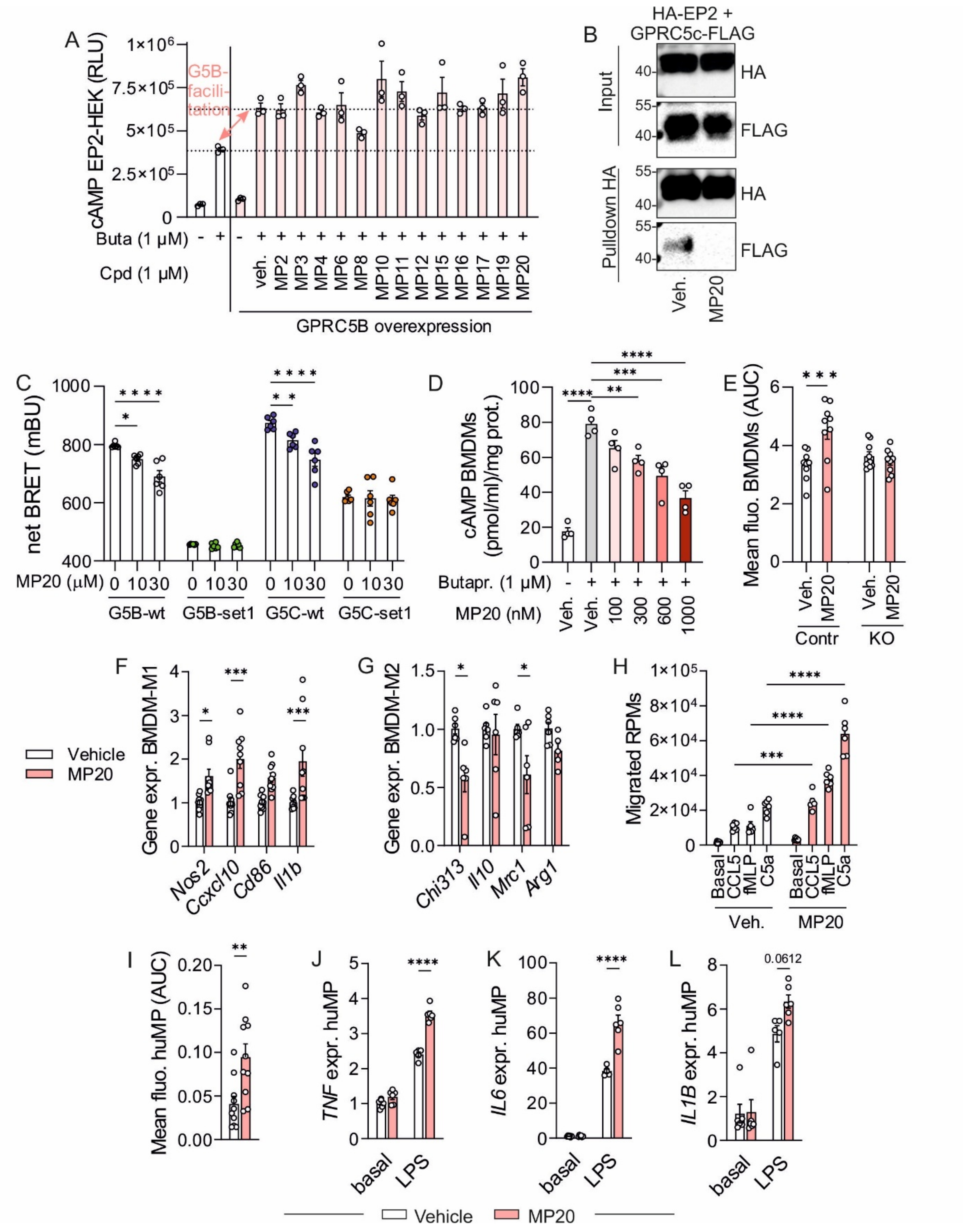

Suppl. Fig. 7: Functional characterization of small molecule inhibitors of GPRC5B/C dimerization. (A) Effect of compounds (10 µM each) on butaprost-induced cAMP production in HEK cells transfected with cAMP GloSensor plasmid, HA-EP2, and GPRC5B-FLAG as indicated (n=3). (**B)** Co-immunoprecipitation of GPRC5C-FLAG with HA-EP2 from HEK cells that were incubated

with vehicle or MP20 (10 µM) for 1 hour. (**C)** BRET between donors GPRC5B-wt-NLuc (G5B-wt), GPRC5B-set1 mutant-NLuc (G5B-set1), GPRC5C-wt-NLuc (G5C-wt), GPRC5C-set1 mutant-NLuc (G5C-set1) and acceptor EP2-mVenus in HEK cells transfected with a donor:acceptor ratio of 1:10; data are expressed as ratio of acceptor emission (535 nm) to donor emission (460 nm) (n=6). **(D)** The effect of different MP20 concentrations on butaprost-induced cAMP production was determined in BMDMs by ELISA (n=4). **(E)** MP20 effect (1 µM) on phagocytosis of pHrodo *E. coli* bioparticles in M0 BMDMs from control mice (Contr) and M-G5b-KOs (KO) (n=3, note that basal difference between control and KO is less pronounced in the presence of vehicle DMSO). **(F,G)** Effect of MP20 (1 µM) on expression of M1 markers in M1-differentiated BMDMs (F, n=9) or M2 markers in M2-differentiated BMDMs (G, n=6) was determined by qRT-PCR (MP20 or vehicle added at beginning of 24 h M1/M2 differentiation). **(H)** MP20 effect (1 µM each) on chemokine-induced migration in RPMs (n=6). **(I)** Effect of MP20 (1 µM) on phagocytosis of pHrodo *E. coli* bioparticles in human blood monocyte-derived macrophages (huMP) (n=10). (**J-L)** Effect of MP20 (1 µM) on basal and LPS-induced gene expression in huMP was analyzed by qRT-PCR (n=6, data normalized to *GAPDH* and basal control set to 1).

Data are means ± SEM; comparisons between treatments were performed using one-way ANOVA with Dunnett´s multiple comparisons test (A,D), two-way ANOVA with Tukey´s (C) or Sidak's (F-H,J-L) multiple comparisons test, or unpaired, two-sided t test (I). n, number of independent experiments or mice; ns, not significant; *, $P < 0.05$; **, $P < 0.01$; ***, $P < 0.001$; ****, $P < 0.0001$.

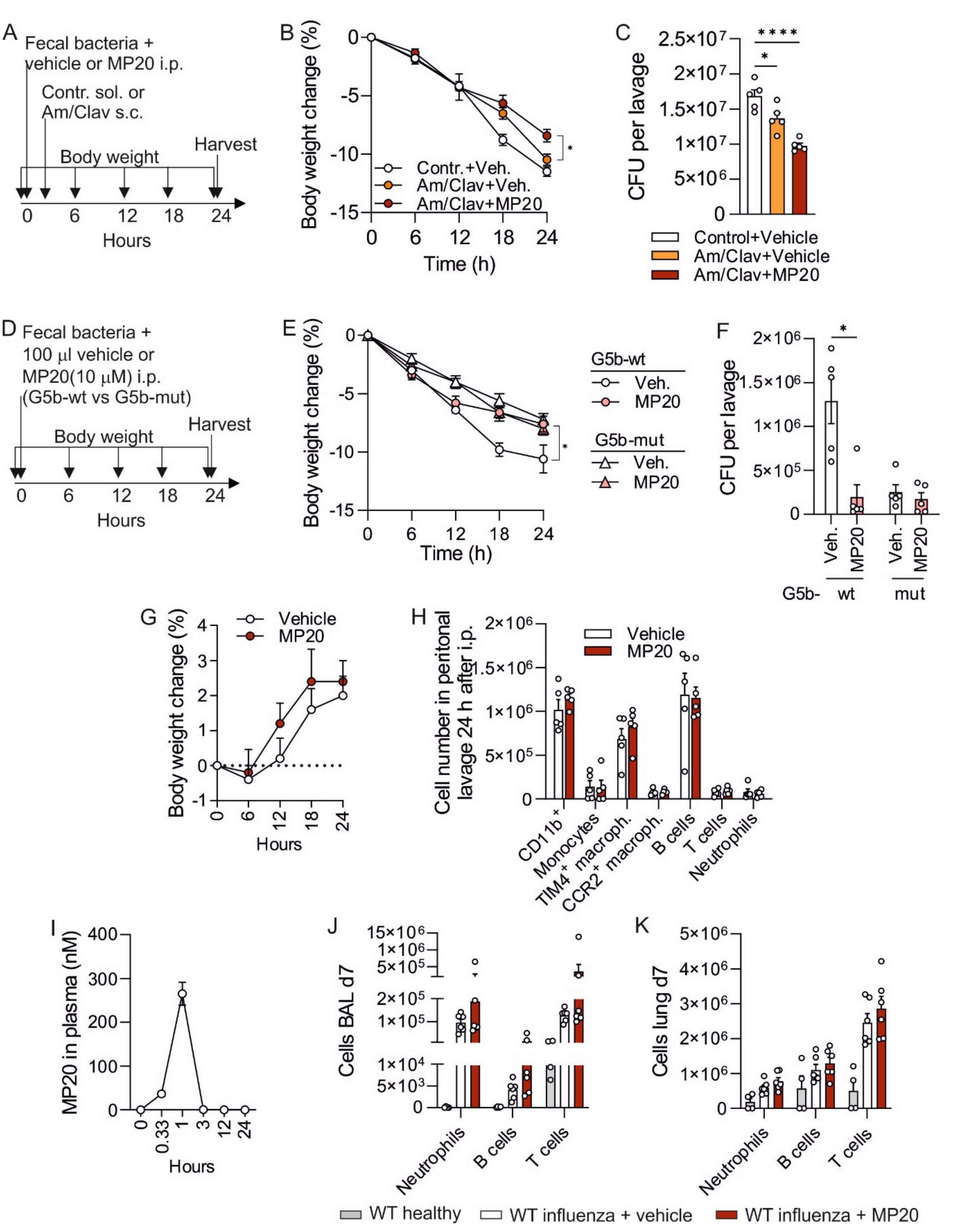

Suppl. Fig. 8: MP20 effect in the basal state and during bacterial peritonitis or influenza A virus infection. (A-C) Bacterial peritonitis with antibacterial treatment: A, Experimental design: Fecal bacteria were co-injected with 100 µl vehicle or 100 µl of a 100 µM MP20 solution; 2 hours later, mice received control solution or amoxicillin/clavulanic acid (Am/Clav) i.p.. (**B,C)** Effect of MP20 treatment on body weight change (B) and number of bacterial CFU in peritoneal

lavage fluid harvested 24 h after injection of fecal bacteria (C) (n=5). **(D-F) MP20 effect on fecal peritonitis in G5b-wt mice and G5b-mut mice:** Experimental design (D), effect of MP20 treatment on body weight change (E) and number of bacterial CFU in peritoneal lavage fluid harvested 24 h after injection of fecal bacteria (F) (n=5). **(G,H) MP20 effect in the healthy peritoneal cavity:** Effect of MP20 (100 µl of a 100 µM solution i.p.) or 100 µl vehicle on body weight (G) or the number of peritoneal myeloid cells ($CD45^+$, $CD11b^+$), monocytes ($CD45^+$, $CD11b^+$, $F4/80^-$, $Ly6C^+$), TIM4-positive resident macrophages ($CD45^+$, $CD11b^+$, $F4/80^+$, $TIM4^+$), CCR2-positive bone marrow-derived macrophages ($CD45^+$, $CD11b^+$, $MHCII^+$, $CCR2^+$), B cells ($CD45^+$, $CD19^+$), T cells ($CD45^+$, $CD11b^-$, $TCR\beta^+$), or neutrophils ($CD45^+$, $CD11b^+$; $Ly6G^+$) in wild-type mice 24 h after application (H) (n=5). **(I)** Plasma MP20 levels were determined by mass spectrometry after i.p. injection of 100 µl of a 100 µM MP20 solution (n=3-6). **(J,K**) **MP20 in IAV infection:** Flow cytometric analysis of neutrophils ($CD45^+$, $Ly6G^+$), B cells ($CD45^+$, $Ly6G^-$, $CD19^+$), or T cells ($CD45^+$, $Ly6G^-$, $TCR\beta^+$) in bronchioalveolar lavage (BAL, J) or digested lung tissue (K) from vehicle- or MP20-treated mice on day 7 after influenza infection (n=6).

Data are means ± SEM; comparisons between vehicle- and MP20-treated samples were performed using one-way ANOVA with Dunnett's post hoc test (C), multiple unpaired t tests (F), two-way ANOVA with Sidak's multiple comparisons test (G-J).

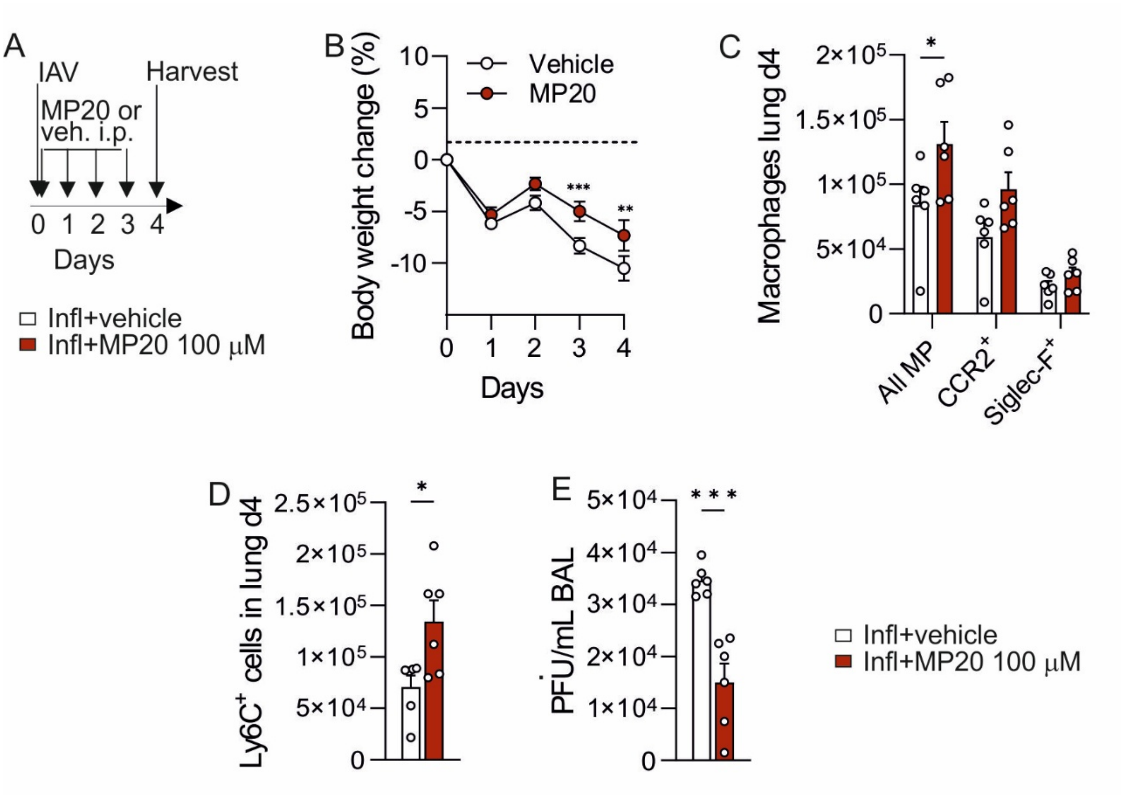

Suppl. Fig. 9: MP20 effect on influenza A virus infection (day 4): (A) Experimental design: 100 µl of a 100 µM MP20 solution or vehicle were injected i.p. on days 0-4 after intratracheal virus application. (**B)** Effect of MP20 on body weight change after influenza infection. (**C,D)** Flow cytometric analysis of macrophage (C) and monocyte (D) populations in digested lung tissue. Cell populations were defined by FACS as follows: All macrophages (MP) ($CD45^+$, $CD11b^+$, $F4/80^+$), $CCR2^+$ bone marrow-derived MP ($CD45^+$, $CD11b^+$, $F4/80^+$, $CCR2^+$), Siglec-F^+ resident MP ($CD45^+$, $CD11b^+$, $F4/80^+$, Siglec F^+), monocytes ($CD45^+$, $CD19^-$, $TCR\beta^-$, $CD11b^+$, $Ly6G^-$, $Ly6C^{hi}$) n=6). **(E)** Statistical evaluation of viral plaques at BAL dilution 1:200 (n=6).

Data are means ± SEM; comparisons between treatment groups were done using two-way ANOVA with Sidak's multiple comparison test (B,C) or unpaired, two-sided t test (D;E). *, $P < 0.05$; **, $P < 0.01$; ***, $P < 0.001$.

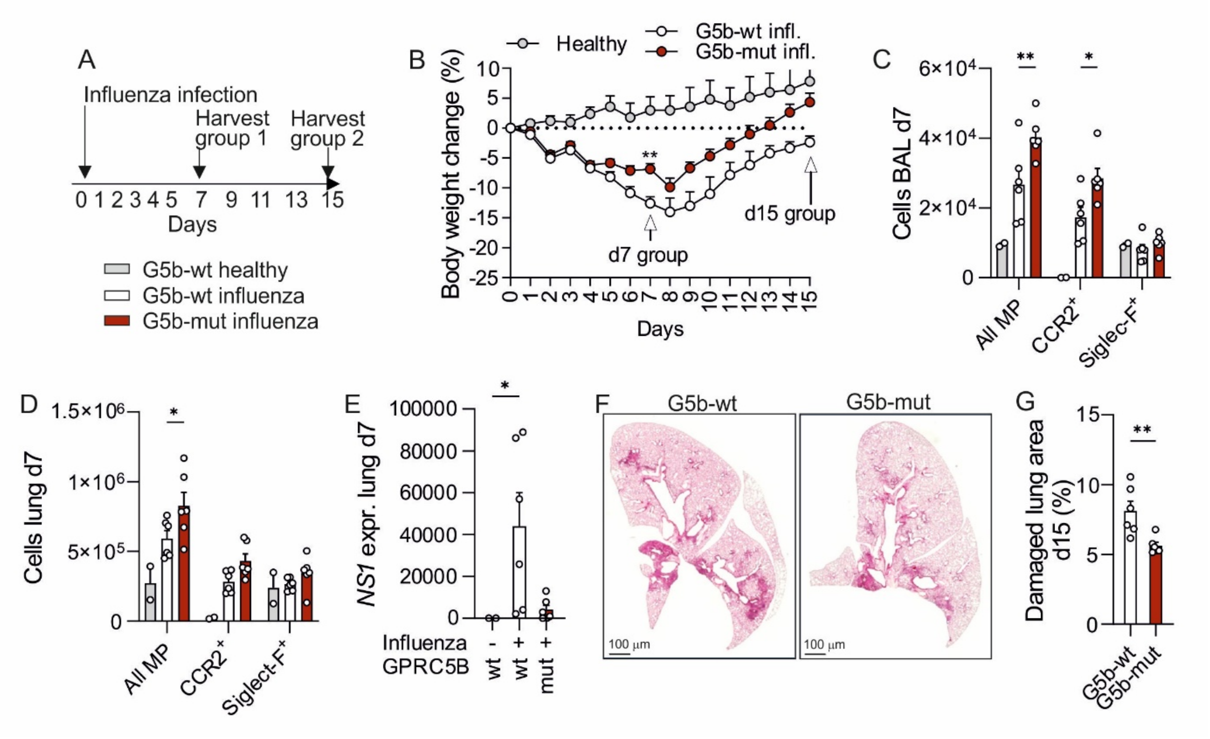

Suppl. Fig. 10: Influenza virus infection in G5b-mut mice. (A) Experimental design. **(B)** Body weight change after influenza infection. **(C-E) Analyses d7:** Flow cytometric analysis of macrophage populations in bronchioalveolar lavage (BAL, C) or digested lung tissue (D); virus load as judged by qRT-PCR detection of viral NS1 (E) (n=7; data normalized to *Gapdh* and non-infected control set to 1). **(F,G) Analyses d15:** Histological analysis of lung damage using H&E staining: exemplary images (F) and statistical evaluation of damaged area (G, n=6).

Data are means ± SEM; comparisons between vehicle- and MP20-treated samples were performed using two-way ANOVA with Sidak's multiple comparisons test (B-D), Kruskal-Wallis test with Dunn's multiple comparisons test (E) or unpaired, two-sided t test (G). n, number of mice; *, $P < 0.05$; **, $P < 0.01$.

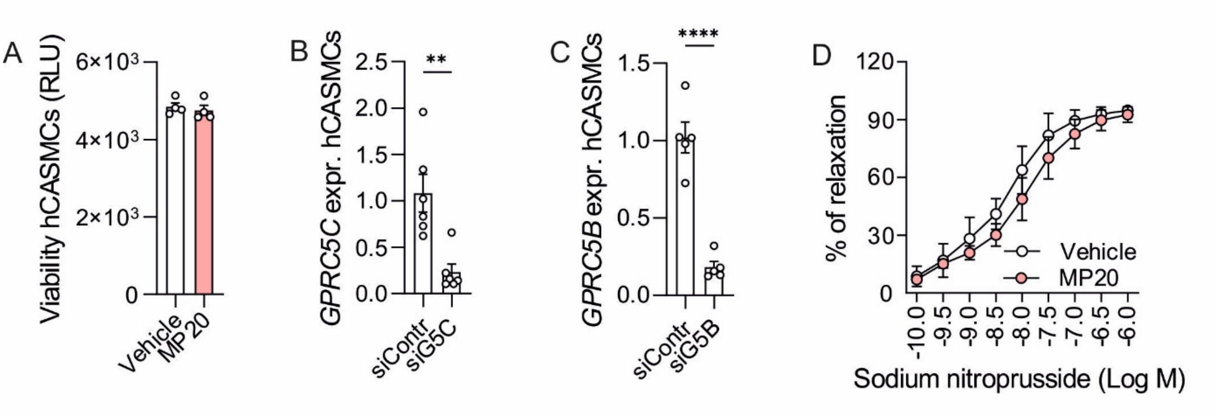

Suppl. Fig. 11: MP20 effect on SMC contractility. (A) Viability of hCASMCs cultured for 1 h in the presence of vehicle or 1 μM MP20 (n=4). **(B,C)** Knockdown efficiency was determined by qRT-PCR in hCASMCs transfected with control siRNA (siContr) or siRNA directed against GPRC5C (siG5C, B, n=6) or GPRC5B (siG5B, C, n=2). **(D)** Sodium nitroprusside-induced relaxation was determined after precontraction with phenylephrine (PE, 10 μM); data are expressed as percentages of maximal PE contraction (n=3).

Data are means ± SEM; comparisons between treatments were performed using unpaired, two-sided t test (A-C) or two-way ANOVA with Sidak's multiple comparisons test (D). n, number of independent experiments or mice; **, $P < 0.01$; ****, $P < 0.0001$.

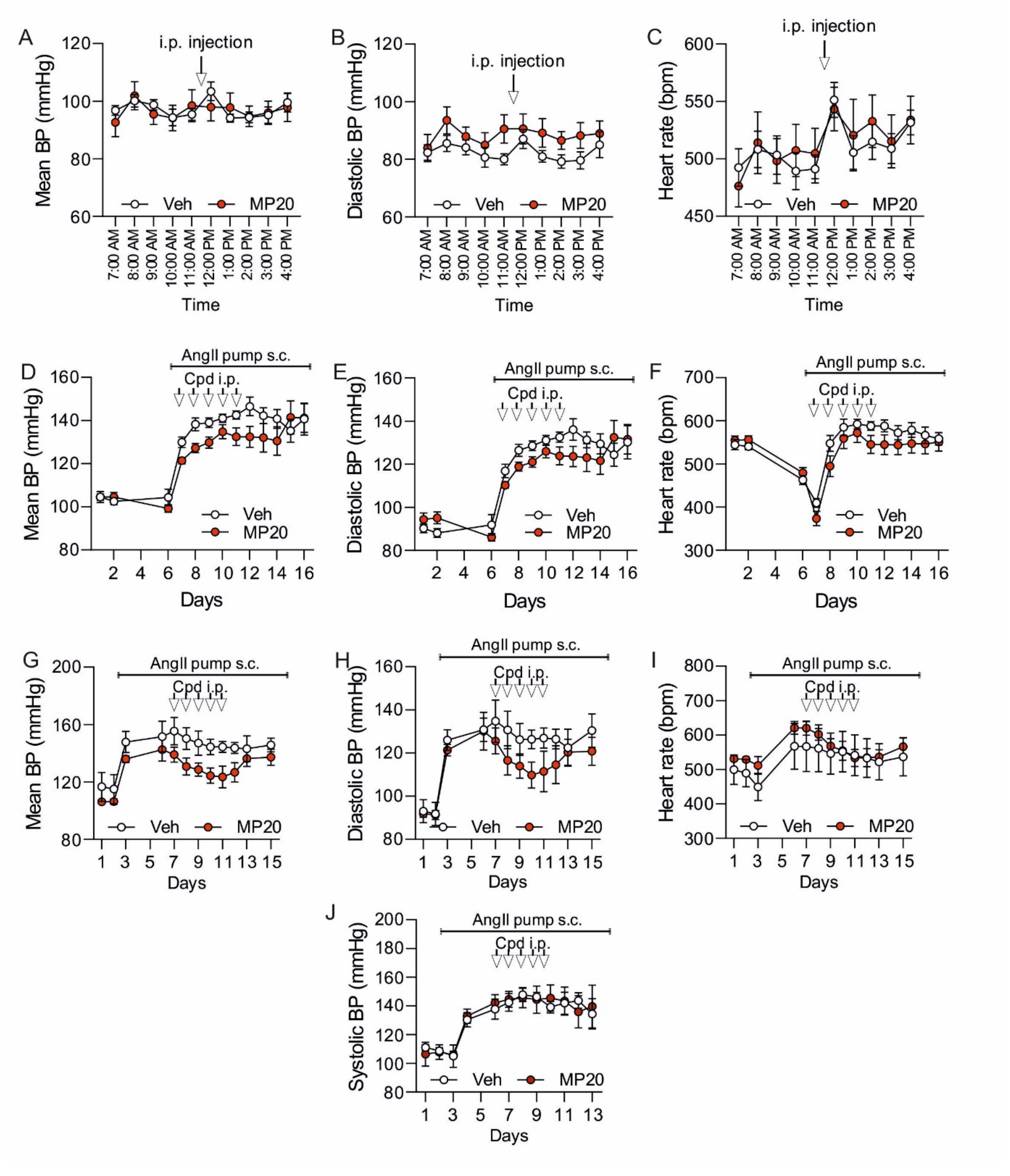

Suppl. Fig. 12: Telemetric recording of mean blood pressure (A,D,G), diastolic blood pressure (B,E,H) and heart rate (C,F,I) in vehicle and MP20-treated wild-type mice in the basal state (one i.p. injection; A-C, n=7 and 9) or after implantation of AngII-releasing mini-osmotic pumps: i.p. injection on five consecutive days in the onset phase of hypertension (D-F, n=13 and 14) or in already established hypertension (G-I, n=5-6). **J,** Effect of vehicle or MP20 injection (100 µl i.p., MP20 stock 100 µM) on five consecutive days in mice with tamoxifen-induced, smooth muscle-

specific double deficiency for GPRC5B and GPRC5C (iSM-G5b/c-KO) and established AngII hypertension.

Data are means ± SEM; comparisons between treatments were performed using two-way ANOVA with Sidak’s multiple comparisons test.

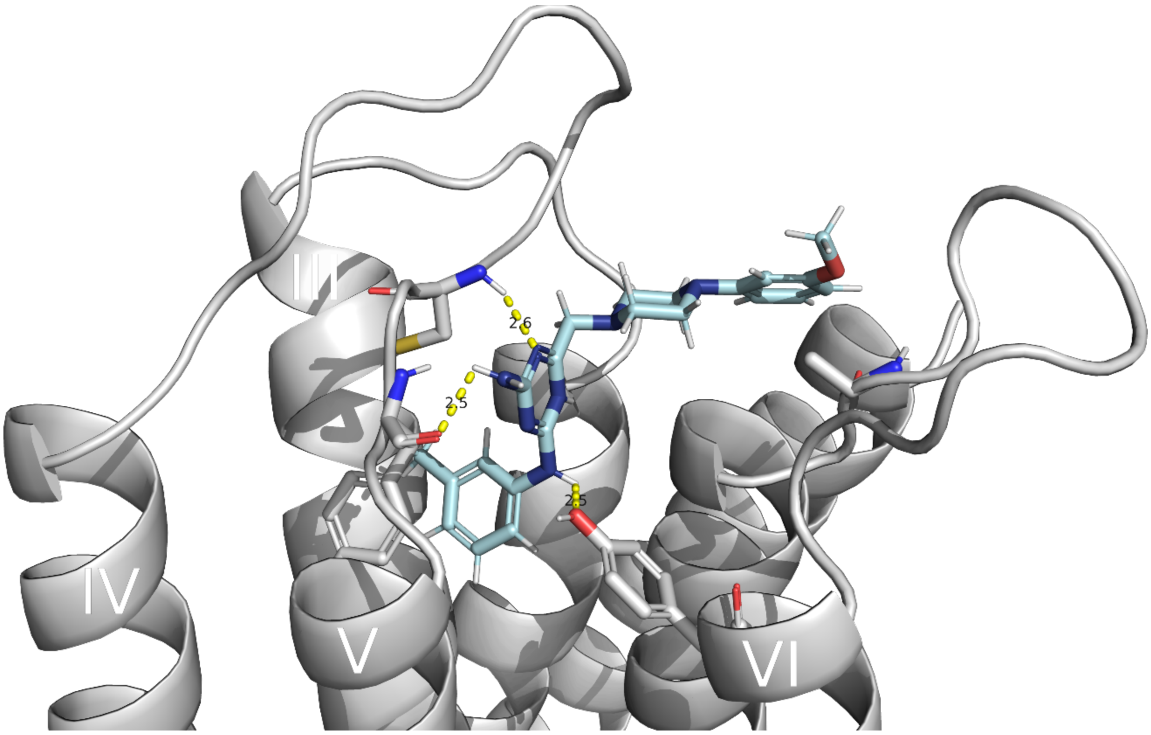

Suppl. Fig. 13: Predicted possible alternative intrahelical binding mode of MP20 in the GPRC5B model obtained with approach 1 (grey).

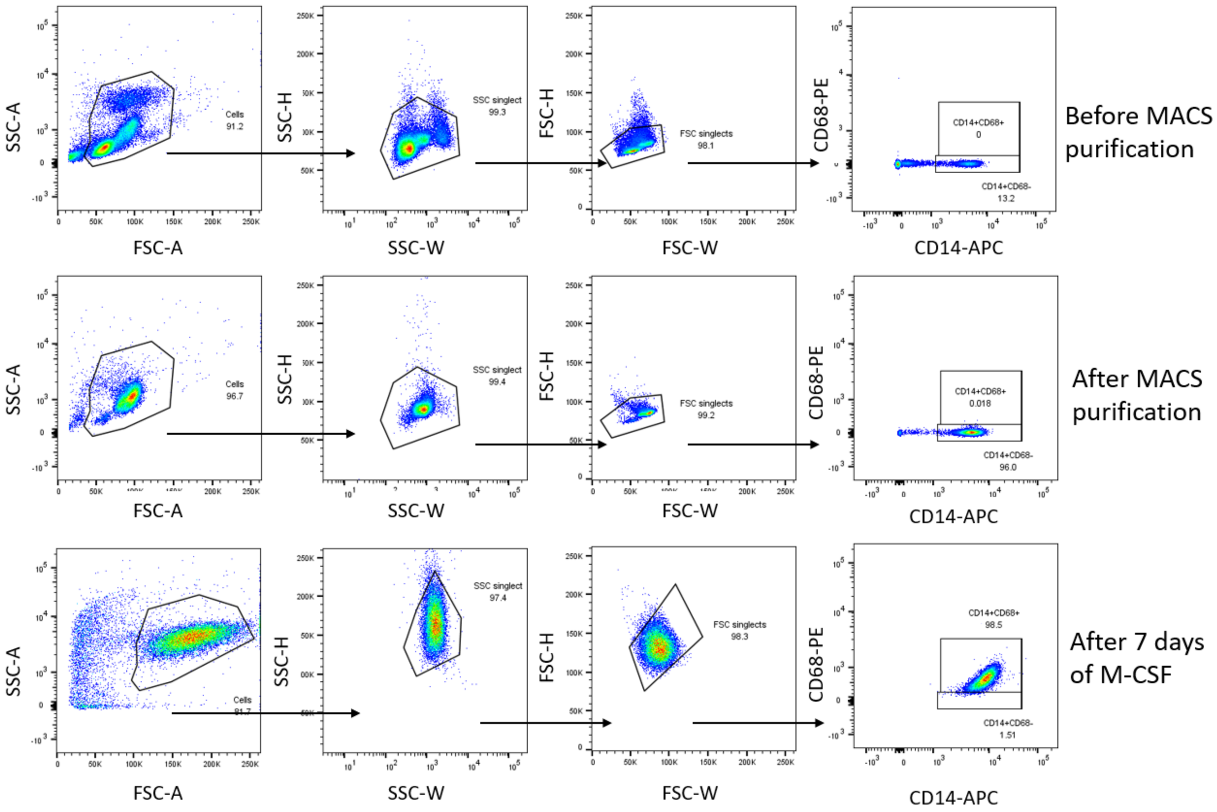

Suppl. Fig. 14: Exemplary FACS plots showing the enrichment of CD14-positive human blood monocytes after MACS (rows 1 versus 2) as well as enrichment of CD68-positive macrophages after 7 days of M-CSF differentiation (row 3).

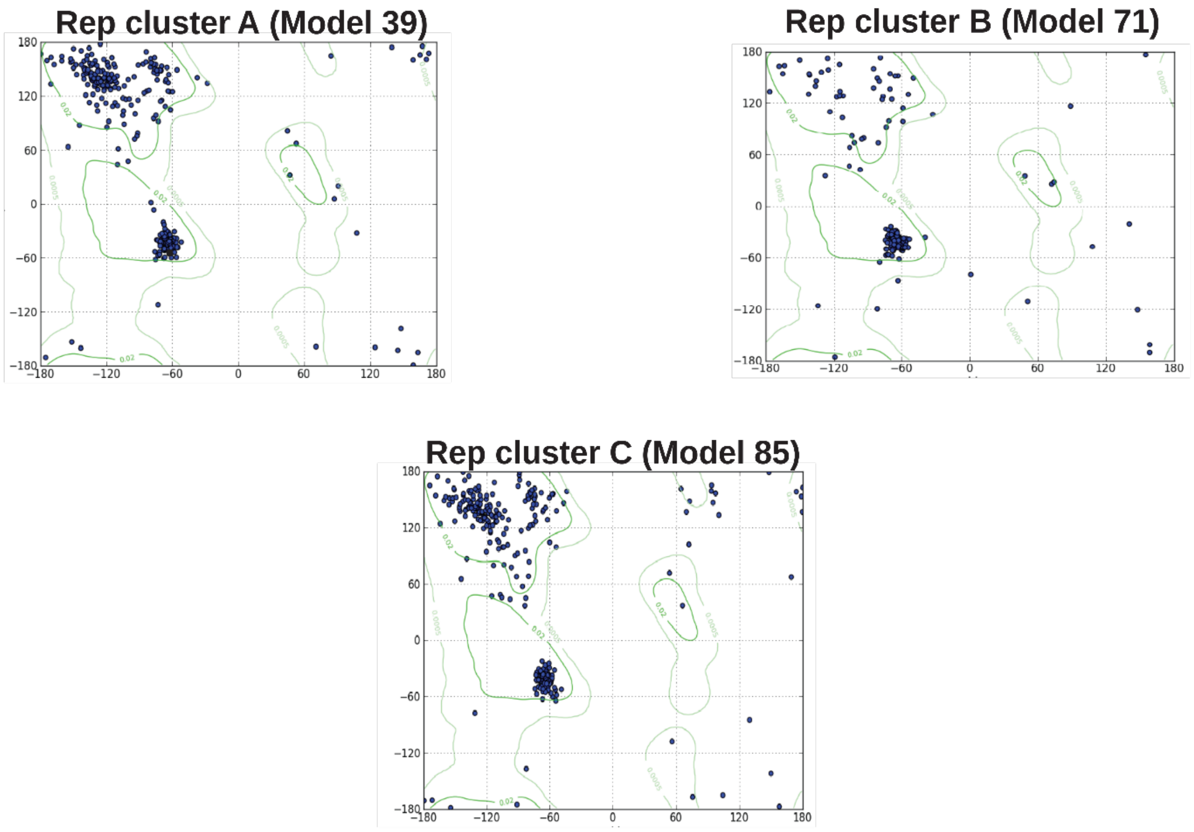

Suppl. Fig. 15: Ramachandran plots for the three homology models mentioned in the main text. Each plot shows the distribution of backbone ϕ (x-axis) and ψ (y-axis) angles. Green lines denote allowed regions.

2. Supplemental Tables

Supplemental Table 1: SMILES, ZINC IDs and sources for all compounds mentioned in this work.

Name	SMILES	ZINC ID	VENDOR	VENDOR ID
K303MP2	*O[C@H](CSC1=NN=C(C2CC2)N1C1CC1)C1=CC=C(F)C=C1*	ZINC000032900848	Enamine	Z240993500
K303MP3	COC1=CC=CC=C1C1=CC(C2=NC([C@H]3CCOC3)=NO2)=NN1	ZINC000096139590	MolPort	029-893-349
K303MP4	Cc1csc(NC(=O)/C=C/c2cnn(Cc3ccccc3)c2)n1	ZINC000008982739	Enamine	Z108818172
K303MP6	CC1=CC(OCC(=O)N2C=NN=C2N)=CC=C1Cl	ZINC000000523636	MolPort	001-607-827
K303MP8	O=C(OC-COC1=CC=CC=C1F)C1=NNC2=CC=CC=C21	ZINC000006370739	Enamine	Z18544149
K303MP10	COC1=CC=C(C=NN2C(=S)NN=C2C(F)(F)F)C=C1OC	ZINC000000565103	MolPort	000-248-785
K303MP11	CC1=C(N=NC2=CC=CC=C2C(F)(F)F)C(=O)ON1	ZINC000100300588	MolPort	016-583-909
K303MP12	O=C(OCc1nc2C(C)=CC=Cn2c1)CCN1C(=O)NC(=O)c2c1cccc2	ZINC000012785831	Enamine	Z215312324
K303MP15	O=C(CCc1cnccn1)N1CCC[C@@H](c2ccn(CCO)n2)C1	ZINC000426693844	MolPort	042-577-353
K303MP16	COC1=CC=C([N+](=O)[O-])C=C1OCC1=NC(N)=NC(N(C)C)=N1	ZINC000009313746	Enamine	Z168984526
K303MP17	O=C(OCC1=CC=CC(F)=C1)C1=NNC(=O)C2=CC=CC=C21	ZINC000746380149	Enamine	Z148042584
K303MP19	C[C@H](Cc1cn(Cc2csc(N)n2)nn1)c1ccccc1	ZINC000067934116	MolPort	019-821-793
K303MP20	COc1cccc(N2CCN(Cc3nc(N)nc(Nc4cccc(C)c4)n3)CC2)c1	ZINC000008973792	MolPort	000-409-522

Supplemental Table 2: Expression vectors ("N" for N-terminal tags; "C" for C-terminal tags)

**Human**				
**Gene**	**Tag**	**Catalog #**	**Source**	**GeneID**
AT1R	N-HA, C-YFP	101659	Addgene	NM_000685.4
PTGIR	N-3xHA	PTGIR0TN00	cDNA.org	AY242134
PTGER1	N-3xHA	n.a.	Self-made	AY275470
PGTER2	N-3xHA	PER020TN00	cDNA.org	AY275471
PTGER2	C-Venus	n.a.	VectorBuilder	NM_000956.4
PTGER3	N-3xHA	n.a.	VectorBuilder	NM_000957.2
PTGER4	N-3xHA	n.a.	Self-made	AY429109
PTGDR1	N-3xHA	n.a.	Self-made	NM_000953
PTGDR2	N-3xHA	n.a.	Self-made	AY507142
TBXA2R	N-3xHA	n.a.	Self-made	AY429110
PTGFR	N-3xHA	PTGFR0TN00	cDNA.org	AY337000
GPRC5A	C-Myc-FLAG	RC200118	Origene	NM_003979
GPRC5B	C-Myc-FLAG	RC205201	Origene	NM_016235
GPRC5B	C-NLuc	Custom-made	VectorBuilder	NM_016235
GPRC5B-mutset1	C-Myc-FLAG	Custom-made	VectorBuilder	NM_016235
GPRC5B-mutset1	C-NanoNluc	Custom-made	VectorBuilder	NM_016235
GPRC5C	C-Myc-FLAG	RC211081	Origene	NM_022036
GPRC5C	C-NLuc	Custom-made	VectorBuilder	NM_022036
GPRC5C-mut7	C-Myc-FLAG	Custom-made	VectorBuilder	NM_022036
GPRC5C-mutset1	C-Myc-FLAG	Custom-made	VectorBuilder	NM_022036
GPRC5C-mutset1	C-NanoNluc	Custom-made	VectorBuilder	NM_022036
GPRC5D	C-Myc-FLAG	RC210138	Origene	NM_018654

Supplemental table 3: Antibodies for immunofluorescence staining

	Catalog #	Clone	Isotype		Source	Dilution
c-Myc-tag	sc-40	9E10	Mouse IgG	Monoclonal	Santa cruz	1:100
CD11b	561688	M1/70	Rat IgG	Monoclonal	BD Biosci-ences	1:500
Rabbit IgG, Alexa Fluor 594	A-21207		Donkey IgG	Polyclonal	Invitrogen	1:500
Mouse IgG, Alexa Fluor 647	A-31571		Donkey IgG	Polyclonal	Invitrogen	1:500

Supplemental Table 4: Primer sequences qRT-PCR

Gene		Sequences	Species
GPRC5B	Forward	CGGGCCTACATGGAGAACAA	Mouse
	Reverse	GGACGCATTTCAGTCCCT	Mouse
GPRC5C	Forward	GAGATGGCCCTGATGCAC	Mouse
	Reverse	CAGGGTTGAGTTGGCACTG	Mouse
GAPDH	Forward	TCCTCAGTGTAGCCCAAGA	Mouse
	Reverse	GGAGAAACCTGCCAAGTATGA	Mouse
Peptidylprolyl iso-merase A (Ppia)	Forward	CGCTTGCTGCAGCCATGGTC	Mouse
	Reverse	CAGCTCGAAGGAGACGCGGC	Mouse
Nos2	Forward	AAGGGGACGAACTCAGTGG	Mouse
	Reverse	CCCGGAAGGTTTGTACAGC	Mouse
Cxcl10	Forward	CCTTGGTCTTCTGAAAGGTGA	Mouse
	Reverse	CATGGCTTGACCATCATCC	Mouse
CD86	Forward	TCTGCCGTGCCCATTTACAA	Mouse
	Reverse	TGTGCCCAAATAGTGCTCGT	Mouse
Il1b	Forward	AGACAGGTCGCTCAGGGTCA	Mouse
	Reverse	AAGTGGTTGCCCATCAGAGG	Mouse
Chi3l3	Forward	AAGAACACTGAGCTAAAAACTCTCCT	Mouse
	Reverse	GAGACCATGGCACTGAACG	Mouse
Il10	Forward	CAGAGCCACATGCTCCTAGA	Mouse
	Reverse	TGTCCAGCTGGTCCTTTGTT	Mouse
Mrc1	Forward	CCACAGCATTGAGGAGTTTG	Mouse
	Reverse	ACAGCTCATCATTTGGCTCA	Mouse
Arg1	Forward	GAATCCTGGTACATCTGGGAAC	Mouse
	Reverse	GAATCTGCATGGGCAACC	Mouse
GPRC5B	Forward	CCGCAGAGATGTGACTCG	Human
	Reverse	TCTCTGATGCCACGAACATT	Human
GPRC5C	Forward	CCCTGATGCACAAAGTTCC	Human
	Reverse	AGGGTCGAGTTGGCACTG	Human
GAPDH	Forward	GCATCCTGGGCTACACTGA	Human
	Reverse	CCAGCGTCAAAGGTGGAG	Human
PR8 NS1	Forward	TGTCAAGCTTTCAGGTAGATTG	
	Reverse	CTCTTAGGGATTTCTGATCTC	

Supplemental Table 5: Antibodies for western blotting

Primary antibody	Catalog #	Clone	Origen	Dilution
GAPDH	2118	14C10	Cell Signaling Technology	1:1000
GPRC5B			Orlandi lab (3)	1:500
FLAG-HRP	A8592	M2	Sigma	1:1000
HA-HRP	H6533	HA-7	Sigma	1:1000
HA	3724	C29F4	Cell Signaling Technology	1:1000
PTGER2	ab167171	EPR8030(B)	Abcam	1:1000

Supplemental Table 6: Antibodies for FACS analysis

Antibody (anti-)	Isotype	Clone	Species	Fluoro-chrome	Source	Catalog #
CCR2	Rat IgG2b, κ	SA203G11	mouse	APC	Biolegend	150627
CCR2	Rat IgG2b, κ	SA203G11	mouse	FITC	biolegend	150607
CD11b	Rat IgG2b, κ	M1/70	mouse, human	BV510	biolegend	101263
CD11c	Armenian Hamster IgG	N418	mouse, human	PE	eBioscience	12-0114
CD19	Rat IgG2a, κ	1D3	mouse	eFluor450	eBioscience	48-0193
CD19	rat IgG2a k	1D3	mouse, human	PE	eBioscience	12-0193
CD45	Rat IgG2b, κ	30-F11	mouse	FITC	BD biosci-ences	553079
CD45	Rat IgG2b, κ	30-F11	mouse, human	eFluor450	eBioscience	48-0451
F4/80	Rat IgG2a, κ	BM8	mouse	PE	Biolegend	123110
F4/80	Rat IgG2a, κ	BM8	mouse	APC-eFluor780	eBioscience	47-4801
Isotype control	Rat IgG2c, k	315		PE-Cy7	BioLegend	400721
Ly6C	Rat IgG2c, κ	HK1.4	mouse	PE-Cy7	Biolegend	128017
Ly6G	Rat IgG2a, κ	1A8	mouse	BV421	biolegend	127627
Ly6G	Rat IgG2a, κ	1A8	mouse	APC	Biolegend	127613
MHCII	Rat IgG2b, κ	M5/114.152	mouse	APC-Cy7	Biolegend	107627
SiglecF	Rat IgG2a, κ	S17007L	mouse	APC	biolegend	155507
TCRβ	Armenian Hamster IgG	H57-597	mouse	BV421	Biolegend	109229
TCRβ	Armenian Hamster IgG	H57-597	mouse	Percp-cy5,5	Biolegend	109227
TIM4	Rat IgG2a, κ	RMT4-54	mouse	Percp-cy5.5	Biolegend	130019

3. Supplemental Methods

Isolation and culture of primary murine macrophage

Resident peritoneal macrophages (RPMs) were isolated from the peritoneal cavities of mice without any stimulation. Peritoneal lavage was performed using 7 ml of ice-cold PBS with 0.5% BSA and 2 mM EDTA. Resident peritoneal macrophages were isolated from peritoneal lavage fluid using the mouse macrophage isolation Kit (peritoneum) (Miltenyi, 130-110-434) according to the manufacturer's protocol. Cells were cultured in RPMI 1640 (Gibco, 21875) supplemented with 10% (v/v) fetal bovine serum (FBS, Gibco, 10270106), 5 U/ml penicillin and streptomycin (Gibco, 15140122) and were used for endogenous CoIP, cAMP assay, migration, or phagocytosis.

Bone marrow derived macrophages (BMDMs) were isolated as follows: Bone marrow was isolated from hindlimbs of mice by flushing out bones with DMEM/F-12 medium (Gibco, 11320) using a 26G needle. $4x10^6$ bone marrow cells were cultured in DMEM/F-12 containing 10% (v/v) FBS, 2 mL L-glutamine (Thermo Fischer Scientific, 25030123), 5 units/ml penicillin and streptomycin, and 100 units M-CSF in 10 cm dish. On day 4, another 5 ml of M-SCF containing medium was added. On day 7, M0 macrophages were used for cAMP assays or phagocytosis. For transwell migration, M0 macrophages were differentiated into M1 macrophages. To do so, cells were treated with 150 units/ml of INF-γ for 6 h and then 20 ng/ml of LPS for 16 h. For M2 differentiation, cells were treated with 40 ng/ml IL-4 for 24 h. To test the effect of MP20 on macrophage differentiation, cells were co-treated with vehicle or MP20 (1 µM) together with INF-γ, LPS, or IL-4.

Experiments with human blood samples were performed according to the regulations of the local ethics committee of the Hessian Regional Medical Board (*Ethikkommission des Fachbereiches Medizin der Goethe-Universität Frankfurt*; AZ 110/11), and informed consent was obtained from all participants. CD14-positve cells were isolated from human peripheral blood of healthy human donors as previously described (4), with some modifications. Peripheral blood mononuclear cells were first enriched using density gradient centrifugation on a Ficoll-Paque premium gradient (Cytiva, 17-5446-02). Centrifugation was performed at 800 xg for 15 min at 20 °C. Next, cells were washed with PBS containing 2 mM EDTA, then resuspended in 500 µl PBS containing 20 mM EDTA and 0.5% BSA and incubated with 7 µl of CD14-APC antibody (Biolegend, 325607) per 10^7 total cells at 4 °C for 10 min. CD14-APC positive cells were isolated with anti-APC microbeads (Miltenyi, 130-090-855) according to the manufacture's protocol. Cells were cultured in RPMI 1640 supplemented with 10% (v/v) FBS, 5 U/ml penicillin and streptomycin and were used for cAMP assay. Monocytes were differentiated into macrophages as previously described (5), with some modifications. Cells were cultured in DMEM/F-12 supplemented with 10% (v/v) FBS, 2 mM L-glutamine, 5 U/ml penicillin and streptomycin, and 50 ng/ml human M-CSF in

10-cm culture dishes. On day 4, another 5 ml of human M-CSF containing medium was added. A FACS-based analysis of monocyte enrichment after MACS as well as of macrophage purity after M-CSF differentiation is shown in Suppl. Fig. 14. On day 7, macrophages were used for cAMP assay, phagocytosis assays.

Culture of cell lines

Human embryonic kidney cells (HEK-293T) were obtained from American Type Culture Collection. Cells were maintained in DMEM supplemented with 10 % (v/v) FBS, 2 mM L-glutamine, 5 units/ml penicillin and streptomycin. Cells were incubated at 37°C in a humidified atmosphere with 5% CO_2.

Human coronary artery smooth muscle cells (hCASMCs) were obtained from Lonza (CC-2583, batch NO. 20TL266549). Cells were maintained in smooth muscle cell growth medium (SmGM-2, Lonza, CC-3181) containing supplements and growth factor (Lonza, CC-4149). Cells were incubated at 37°C in a humidified atmosphere with 5% CO_2.

Madin-Darby Canine Kidney II (MDCKII) cells were obtained from American Type Culture Collection. Cells were maintained in DMEM supplemented with 10 % (v/v) FBS, 2 mM L-glutamine, 5 units/ml penicillin and streptomycin. Cells were incubated at 37°C in a humidified atmosphere with 5% CO_2.

Transfection

For overexpression experiments, HEK-293T cells were transfected with expression vectors listed in Supplemental Table 2 using Opti-MEM (Gibco, 31985062) and Lipofectamine 2000 transfection reagent (Invitrogen, 11668019) according to the manufacturer's instructions.

For siRNA-mediated knockdown experiments, HEK-293T or hCASMCs were transfected with 28.5 nM of siRNA directed against human GPRC5B (Sigma-Aldrich, SASI_Hs01_00171699, NM_016235, target sequence: 5'-CGUUUAGAAGCAACGUGUA-3') or human GPRC5C (Qiagen, SI00122535, target sequence: 5'-GTGGATCGTCATGTATACTTA-3', and SI00122549, target sequence: 5'-CTGGGCCTTCGTCCTCTTCTA-3') using Opti-MEM and Lipofectamine RNAiMAX (Invitrogen, 13778075) according to the manufacturer's instructions. siRNA Universal Negative Control #1 (Sigma-Aldrich, SIC001) was used as a control for GPRC5B knockdown. AllStars Negative Control siRNA (Qiagen, 1027281) was used as a control for GPRC5C knockdown.

For combined overexpression and knockdown in HEK cells, HEK-293T were transfected with 28.5 nM of siRNA directed against target gene using Opti-MEM and Lipofectamine

RNAiMAX according to the manufacturer's instructions. After 20-24h, cells were transfected with expressing vectors using Opti-MEM and Lipofectamine 2000 transfection reagent. After 48h, cells were used for experiments.

Transwell migration

Murine peritoneal cells were resuspended at a density of $3x10^5$ cells/ml in RPMI 1640 containing 10% FBS and LPS 1 μg/ml. 100 μl of the cell suspension was added to 8 μm pore size 96-well inserts (Corning, 3374). To assess the effect of decoy peptide or compound, cells were treated with either peptides (1 μM) or compound (1 μM) in the suspension. The lower wells contained either 250 μl of medium alone (RPMI 1640 with 5% FBS) or 250 μl of medium containing CCL5 (75 ng/ml), C5a (20 ng/ml), or fMLP (10 nM). Cells were allowed to transmigrate for 24 h at 37 °C and 5% CO_2, then inserts were discarded and transmigrated cells collected from the bottom well. After washing cells in cold PBS, the number of transmigrated cells was determined by flow cytometry. In order to count the absolute cell number by flow cytometry, AccuCount Fluorescent Particles were used. For the analysis, CD45-FITC and F4/80-PE were used.

Murine M1 BMDMs were suspended at a density of $1x10^6$ cells/ml in DMEM/F-12. 100 μl of the cell suspension was added to 8 μm pore size 96-well inserts. The lower wells contained either 250 μl of medium alone (DMEM/F-12 with 5% FBS) or medium containing CCL5 (75 ng/ml), CCL2 (10 ng/ml), SDF-1β (100 ng/ml), C5a (20 ng/ml), or fMLP (10 nM). After 3 h of incubation at 37°C and 5% CO_2, transmigrated cells were measured by flow cytometry. In order to count the absolute cell number by flow cytometry, AccuCount Fluorescent Particles (Spherotech, ACFP-100-3) were used. For the analysis, CD45-FITC and F4/80-PE were used.

Phagocytosis assay of *E. coli*

To assess phagocytotic activity, macrophages from different sources (RPMs, BMDMs, and human monocyte-derived macrophages) were used.

For RPMs, MACS-isolated cells were resuspended at a density of 1×10^6 cells/ml in RPMI 1640 supplemented with 10% FBS and 100 ng/ml LPS, and 100 μl of the suspension was seeded into 96-well plates. After 6 h, the medium was replaced with 90 μl of serum-free, phenol red–free medium (Gibco, 21041025) containing Hoechst 33342 (Thermo Fisher Scientific, H3570) to enable cell quantification. To test the effect of the decoy peptide or compound, cells were treated with 1 μM (final concentration) for 1 h. Subsequently, 100 μl of serum-free, phenol red–free medium containing pHrodo Deep Red *E. coli* bioparticles (1 mg/ml; Invitrogen, P35360) was added.

For BMDMs, M0-polarized cells were seeded at the same density in DMEM/F-12 with 10% FBS. After 24 h, the medium was exchanged for 90 μl of serum-free, phenol red–free medium

containing Hoechst 33342. Cells were then treated with compound (300 nM, 300 nM, or 1 µM final concentration) for 1 h, followed by the addition of *E. coli* bioparticles as described above. To assess the inflammatory gene expression after treatment with *E. coli* bioparticles, cells were washed three times with cold PBS 3 h after treatment and processed for qRT-PCR.

For human monocyte-derived macrophages, cells were seeded at 1 × 10^6 cells/ml in DMEM/F-12 with 10% FBS. After 6 h, the medium was replaced with Hoechst-containing serum-free medium, and cells were treated with compound (1 µM final concentration) for 1 h before addition of *E. coli* bioparticles.

Live cell imaging of pHrodo fluorescence was performed using the Zeiss Axio live cell imaging system (10x objective lens) in an environmental chamber at 37°C. Images were collected for 6 h every 10 min. For the quantification, integrated density was determined using Fiji and resulting values were normalized to the numbers of cells.

Immunoprecipitation

For immunoprecipitation of overexpressed proteins, HEK-293T cells were lysed with RIPA buffer supplemented with protease/phosphatase inhibitors for 15 min on ice. To assess the effect of the compound or decoy peptide, cells were treated with compound (10 µM) or vehicle solution for 1 h before lysis. The lysates were centrifuged at 13,000 rpm for 15 min at 4 °C and protein concentration was determined by BSA assay. 600-700 µg of lysate was incubated with 20 µl of HA-tagged magnetic beads (MBL International, M180-11) or FLAG-tagged magnetic beads (Thermo Fisher Scientific, M8823) at 4 °C with gentle rotation for 2h. After 3 washes with cold RIPA buffer, proteins bound to the beads were eluted by boiling the beads in 60 µl of 4x Laemmli sample buffer at 50 °C for 5 min and then separated by SDS-PAGE, transferred to a nitrocellulose membrane and immunoblotted with indicated antibodies. Target proteins were visualized by enhanced chemiluminescence reagent and a ChemiDoc MP Imaging System using Image Lab Software (Bio-Rad). Per sample, 600-700 µg of protein was used for immunoprecipitation and 20 µg of total lysates were used for input controls.

For immunoprecipitation of endogenous EP2, RPMs were lysed in RIPA buffer supplemented with protease/phosphatase inhibitor cocktail for 15 min on ice. To assess the effect of the compound or decoy peptide, cells were treated with compound (10 µM) or decoy peptide (10 µM) for 1 h before lysis. The lysates were centrifuged at 13,000 rpm for 15 min at 4 °C. Supernatant was incubated with 10 µl of the anti-EP2 (Abcam, ab167171) at 4 °C with gentle rotation overnight. Thereafter, 20 µl of A/G-Sepharose beads (Santa Cruz, sc-2003) were added and incubated for 1h at 4 °C with gentle rotation. After 3 times washed with cold RIPA buffer, proteins bound to the beads were eluted with 40 µl of 4x Laemmli sample buffer at 50 °C for 5 min. Samples were

subjected to SDS-PAGE, transferred to a nitrocellulose membrane and immunoblotted with Veri-Blot IP detection reagent (Abcam, ab131366). Per sample, 500 µg of protein was used for immunoprecipitation and 20 µg of total lysates were used for input controls. Normal rabbit IgG (Cell Signalling Technology, 2729) was used as negative control.

cAMP assays

For the examination of cAMP levels in HEK cells, HEK-293T cells were transfected with plasmids containing cDNAs encoding GPRC5B or GPRC5C (0.1 µg) and the indicated prostanoid receptors (0.1 µg). In knockdown experiments, cells were first transfected with siRNAs targeting human GPRC5B or/and GPRC5C and, on the following day, with plasmids encoding the indicated prostanoid receptors (0.1 µg). After 24h of plasmid transfection, 100 µl of cells ($4x10^5$ cell/ml) were transferred to a 96-well plate and after another 16-20 h medium was replaced with serum-free DMEM containing 50 µM IBMX for 30min. Thereafter, ligands were added for 30 min. The cAMP levels in cell lysates were measured by using direct cAMP kit (Enzo lifesciences, ADI-900-066) according to the manufacturer's protocol. Protein concentration of the same samples was determined by BSA protein quantification assay. Data were normalized to the protein amounts ((cAMP pmol/ml)/mg of protein).

In order to determine cAMP levels in macrophages, RPMs, M0 BMDMs, or human blood monocyte-derived macrophages were seeded at 10^5 cells per well onto 96-well plate. The next day, the medium was replaced with serum-free medium containing 50 µM IBMX with or without compound (100 nM, 300 nM, 600 nM, 1 µM or 10 µM) or decoy peptide (1 µM) for 30 min and then ligands were added for 30 min. cAMP levels in cell lysates were measured using the same kit as for HEK-293T cells.

In order to determine cAMP levels in human blood monocytes, monocytes were seeded at 10^5 cells per well onto 96-well plate. Medium containing 50 µM IBMX with or without compound (10 µM) or decoy peptide (1 µM) were added and incubated for 30 min. Then, ligands were added for 30 min. cAMP levels in cell lysates were measured using the same kit as for HEK-293T cells.

In order to determine cAMP levels in huCASMCs, cells transfected with siRNAs targeting human GPRC5B or GPRC5C. After 16-20 h, cells were seeded at 10^5 cells per well onto 96-well plate. The next day, the medium was replaced with serum-free medium containing 50 µM IBMX for 30 min and then ligands were added for 30 min. cAMP levels in cell lysates were measured using the same kit as for HEK cells.

For GloSensor-based determination of cAMP levels, HEK-293T cells were transfected with plasmids containing cDNAs encoding GPRC5B (0.1 µg), the indicated prostanoid receptors

(0.1 µg), and the GloSensor 22F (0.1 µg; Promega, E2301). After 24h, 100 µl of cells ($4x10^5$ cell/ml) were transferred to a 96-well plate. The next day, the medium was replaced with serum-free DMEM containing 50 µM IBMX and 3 mM luciferin D. For assays without compounds, ligands were added 2 h after the addition of luciferin D. For assays with compounds, cells were incubated with luciferin D for 1 h, then treated with compounds (10 µM) for 1 h before ligand stimulation. Luminescence was recorded for 30 min after ligand addition using a plate reader (FlexStation 3). The area under curve of the luminescence traces was calculated by using SoftMax Pro software.

Immunofluorescence staining

For immunofluorescence staining in HEK cells, sterilized circular coverslips were placed in a 48-well plate and coated with poly-D-lysine (2 µg/cm^2, Sigma-Aldrich, P9155) for 45 min at 37 °C, followed by laminin coating (1 µg/cm^2, Sigma-Aldrich, L2020) overnight at 37 °C. HEK cells (200 µl at 4×10^5 cells/ml) were seeded onto the coverslips. The following day, the medium was removed and cells were washed twice with HBSS. Cells were then incubated with WGA (5 µg/ml, Biotium, W11261) for 10 min at room temperature. After three washes with HBSS, cells were fixed with 4% paraformaldehyde (PFA) for 10 min at room temperature and washed three times with HBSS. Blocking was performed with PBS containing 2% BSA for 30 min at room temperature, followed by incubation with primary antibodies for 90 min at room temperature. Primary antibodies are listed in Supplemental Table 3. After primary antibody incubation, cells were washed three times with PBS and incubated with secondary antibodies (1:500 dilution) for 1 h at room temperature. Nuclei were counterstained with 4′,6-diamidino-2-phenylindole (DAPI, D3571, 1:1000 dilution). After three washes with PBS, coverslips were mounted using Fluoromount W (Serva, 21634.01). For imaging, single optical sections through the axial center of the cells were acquired using an LD LCI Plan-Apochromat 63×/1.2 Imm Korr DIC M27 objective on a Zeiss LSM 880 confocal microscope equipped with an Airyscan 2 detector in super-resolution (SR) mode. Pearson's correlation coefficient was calculated using FIJI.

For immunofluorescence staining in resident peritoneal macrophages (RPMs), 200 µl of cells (1×10^6 cells/ml) were seeded onto sterilized circular coverslips coated with poly-D-lysine (2 µg/cm^2) for 30 min at 37 °C. After 3 h, cells were washed twice with HBSS and fixed with 0.4% paraformaldehyde (PFA) for 10 min at 4 °C, followed by three washes with HBSS. Cells were then blocked with 2% BSA in PBS for 30 min at room temperature and incubated with primary antibodies for 90 min at room temperature. After primary antibody incubation, cells were washed three times with PBS and incubated with secondary antibodies (1:500 dilution) together with CD11b antibody (cell surface marker) for 1 h at room temperature. Cells were then washed three times with PBS and mounted using PermaFluo Aqueous Mounting Medium (Epredia, TA-030-FM).

Images were acquired using a Zeiss LSM 880 confocal microscope equipped with an Airyscan 2 detector. Pearson's correlation coefficient was calculated using FIJI. Antibody information is provided in Supplemental Table 3.

Cell viability test

A total of 100 µl of the cell suspension (4 × 10^5 cells/ml) was transferred to 96-well plates. After 16–20 h, the medium was replaced with fresh medium containing vehicle or compound (1 µM). Following the indicated incubation period, cell viability was assessed using the CellTiter-Fluor Cell Viability Assay (Promega, G6080) according to the manufacturer's instructions. Luminescence was measured with plate reader (Flexstation 3).

Quantitative real-time reverse transcription polymerase chain reaction (qRT-PCR)

RNA was extracted using Quick RNA micro kit (Zymo, R1050) and complementary DNA was synthesized by reverse transcription of extracted RNA using and ProtoScriptII (NEB, M0368) according to the manufacturer's instruction. Quantitative real-time PCR was performed using SYBR green PCR mix (Applied Biosystems, 4368708) using a Light Cycler 480 II (Roche) and QuantStudio 1 (Applied Biosystems). The relative expression levels of each gene were calculated using ΔΔ Ct method and normalized to the endogenous control (GAPDH). For the primer sequences, see Supplemental Table 4.

Western blotting

Samples were lysed in RIPA buffer (100 mM Tris-HCl pH 7.5, 5 mM EDTA, 50 mM NaCl, 50mM β-glycerophosphate, 50 mM NaF, 0.1 mM Na_3VO_4, 0.5% NP-40, 1% TritonX-100, 0.5% sodium deoxycholate) supplemented with protease/phosphatase inhibitors (Thermo Fisher Scientific, 78445). Proteins were separated by SDS-PAGE and transferred onto nitrocellulose membranes (Cytiva, 10600003). After blocking in 5% (w/v) or 3% (w/v) BSA for 1h at room temperature, membranes were incubated overnight at 4°C with primary antibodies (Supplemental Table 5). The membranes were then washed three times with TBST for 15 min and incubated with horseradish peroxidase-conjugated antibodies directed against rabbit IgG (1:1000, Cell Signalling Technology, 7074) for 1 h at room temperature. Target proteins were visualized by enhanced chemiluminescence reagent (Millipore, WBKLS0500) and a ChemiDoc MP Imaging System using Image Lab Software (Bio-Rad). Quantification of band intensities was done using Fiji software.

FACS

Cells were pelleted by centrifugation (500 xg for 10 min at 4°C) and resuspended in PBS. For each 200 µl of cells, 1 µl of antibody per 10^7 cells was added. The cells were incubated for 10 min at room temperature in the dark, then 400 µl of PBS was added. Cells were pelleted by centrifugation (500 xg for 10min at 4°C) to remove non-binding antibodies, and then resuspended in 200-500 µl of PBS and analysed by FACS. 2.5 µl of AccuCount Fluorescent Particles was added into each sample to count the absolute cell number by flow cytometry. Antibodies are listed in Supplemental Table 6.

For the analysis of peritoneal leukocytes during peritonitis, we used CD45-FITC, CD11b-BV510, Ly6G-BV421, CD19-eFluor450, TCRβ-BU421, Ly6C-PE-Cy7, F4/80-PE, MHCII-APC-Cy7, TIM4-PerCP-cy5.5, and CCR2-APC (populations defined according to (6, 7)).

For the analysis of lung and BAL leukocytes during influenza virus A-infection, we used CD45-FITC, CD19-PE, Ly6G-APC, CD11b-BV510, TCRβ-Percp-cy5.5 (leukocytes and neutrophils); CD45-eFluor450, CD11b-BV510, Ly6C-PE-Cy7, F4/80-APC-eFluor780, SiglecF-APC, CCR2-FITC, and CD11c-PE (macrophages) (populations modified from (8)).

For the analysis of peritoneal leukocytes at basal state, we used CD45-FITC, CD19-PE, TCRβ-BV421, CD11b-BV510, Ly6G-APC.

All flow cytometric analyses were performed using a FACS Canto II flow cytometer (BD Biosciences). Gating strategies are shown in chapter 3 of the supplemental materials. FlowJo software (verson 10.8.1) was used for data analysis.

Compound screening

Homology model generation: Given the unavailability of an experimental structure for the GPCR5b receptor, homology modelling was applied to obtain structural information. Three modelling approaches were used:

1. Based on a BLAST alignment, mGluR1 and mGluR5 were chosen as template sequences, being the only ones having an available resolved experimental structure as of March 2020 (identity scores: 25.38 % and 23.74 %, respectively). The template-target sequence alignment was performed using PROMALS3D (9) and refined by correcting gaps and misalignments with Molecular Operating Environment (MOE; 2019.0101 Chemical Computing Group ULC, 910-1010 Sherbrooke St. W., Montreal, QC H3A 2R7, 2019) until the best alignment was obtained. A multi-template approach was chosen for the modelling to have more structural information to rely on and compensate for the low sequence identity. The mGluR1 allosteric ligand FITM was included in the sequence alignment and structure template, as present in the experimental structure (PDB ID: 4OR2) (10). A first set of

10 models was generated, using the MODELLER(11) automodel class and Discrete Optimized Protein Energy (DOPE) scores were calculated to evaluate the accuracy of the generated models.

Using these templates and alignment, 200 models were obtained and clustered with the Ensemble cluster of UCSF Chimera. From the 13 clusters generated, the representatives of the top 3 most populated clusters were selected. The Ramachandran plots showed that most of the residues were within the allowed values (Suppl. Fig. 15). Following the initial round of docking screening, only the model that produced the highest number of promising poses in visual inspection was chosen for further calculations.

2. The model of GPRC5B in the AlphaFold(12) protein structure database was also extracted and prepared for docking.
3. For generating the model of the GPRC5B-EP2 complex, SWISS-MODEL (13) was used for generating a model of GPRC5B, while for the EP2 receptor, the experimental structure was used (PDB ID: 7CX2). However, due to substantial gaps in the EP2 structure, the missing loops were modelled with SWISS-MODEL. Identifying conserved residues within GPRC5B was followed by protein-protein *ab initio* docking with the HADDOCK2.4 (14) server to identify possible dimerization sites and generate the model of the dimer. Following the confirmation of GPRC5B's interaction sites with EP2 through mutagenesis, we refined the complex model to align with the experimental data and membrane orientation. The details have been explained previously (15).

For a comparison of the different model structures, please refer to Suppl. Data File 2 "Comparison of the binding sites between the different models" and spreadsheet "Suppl_data_3_pocket_rmsd_analysis.xlsx", which contains all calculated data.

Molecular docking calculations: All the selected models were prepared by using the Protonate3D method, as present in MOE software, which was used for protonation of the system. Histidines' protonation was then manually adjusted according to the local environment, as well as for cysteines involved in disulfide bridges. N- and C-Termini were capped, and chain breaks were protonated as in the amidic bond. Unresolved side chains were added and minimized using the MOE built-in force field CHARMM.

DOCK3.7 (16) was chosen as the docking software. Matching spheres were generated using dummy atoms to define the binding site. The subsets of ZINC20 (17) lead-like and drug-like were used as virtual libraries for the docking screening. Poses were clustered using Daylight fingerprints and Tanimoto similarity as implemented in the Butina Clustering method (18), and the top-cluster representatives were extracted for each docking, for a total of 5390. The extracted molecules were then inspected visually, and those with unfavorable interactions were discarded.

The compounds' conformation library was generated in-house in the db2 format suitable for DOCK for the docking calculations of analogs of original ZINC compounds.

The top-scored poses of each calculation were then clustered based on Tanimoto similarity, and cluster representatives were visually inspected.

Control calculations to the intrahelical binding site: Molecular structures were converted from SMILES representations into protonated (pH 7.4), energy-minimized three-dimensional conformations in PDBQT format using the TLDR server (19). The docking grid was defined based on structural alignment to the mGluR1 crystal structure (PDB ID: 4OR2) and centered on the binding site of the co-crystallized ligand (10). Docking simulations were carried out using the models described in approaches 1 and 3 above. For each compound, AutoDock Vina 1.2 was used to generate up to 20 binding poses (20). By default, Vina excludes poses whose predicted binding affinity differs by more than 3 units from the top-scoring pose for the same ligand. The docking poses of MP20, MP11, and MP8 were subsequently inspected visually for qualitative assessment. The best-scoring pose obtained for MP20 in the model obtained with approach 1 is shown in Suppl. Fig. 13.

Bioluminescence resonance energy transfer (BRET)

The NanoBRET assay was performed following the technical manual for the NanoBRET Protein:Protein Interaction System (Promega). In brief, cells were transfected with different amounts of plasmids C-terminally fused with NLuc or venus via a linker encoded by DNA sequence 5'-GGTGGCACCGGTGGATCC-3'; the control vector was expressing only NLuc. After 24 h, 100 µl of cells ($4x10^5$ cells/ml) were transferred to a poly-l-lysine-coated clear bottom white wall 96-well plate. After another 16-20 h, medium was replaced with 75 µl of serum-free Opti-MEM with or without MP20 (10 µM) and cells were incubated for 1 h. Thereafter, 25 µl of Nano-Glo Substrate (25 µl of 5X solution in 2.5 ml of Opti-MEM) (Promega, N1571) was added and mixed for 30 s. Readings were performed within 10 min using Flexstation3 at 535 nm (acceptor, Venus) and 460 nm (donor, NLuc) with an integration time of 1 s. The raw BRET ratio was calculated by dividing the acceptor emission value (535 nm) by the donor emission value (460 nm), and further converted into milliBRET units (mBU) by multiplying by 1000.

Calcium mobilization assays

HEK-293T cells were transfected with plasmids encoding indicated cDNAs together with chimeric Gα protein $G\alpha_K$ and the calcium sensitive GFP–aequorin fusion protein G5A (21) using Opti-MEM and Lipofectamine 2000, according to the manufacturer's instructions. After 20-24 h, 100 µl of cells ($4x10^5$ cell/ml) was seeded into to a poly-l-lysine-coated clear bottom white wall 96-well

plate. On the following day, the medium was replaced with HBSS containing 10 mM HEPES and 5 μM coelenterazine h (Promega, S2011) for 2 h. Calcium transients were recorded for 2 min after stimulation with AngII (1 μM) using a plate reader (Flexstation 3). The area under curve of the luminescence traces was calculated with SoftMax Pro software.

IP1 assay

huCASMCs were transfected with siRNA targeting the indicated genes using Opti-MEM and Lipofectamine RNAiRMX according to the manufacturer's instructions. After 48, IP1 levels were measured with the IP-One Gq HTRF kit (Cisbio, 62IPAPEB) according to the manufacturer's instructions. Briefly, cells were trypsinized and resuspended in 1x Stimulation Buffer (4,000 cells in 7 μl). A total of 7 μl of the cell suspension was mixed with 7 μl of Stimulation Buffer containing vehicle, or AngII (1 μM), or iloprost (1 μM) in the presence or absence of compound (1 μM), and seed into white opaque 384-well plate (PerkinElmer, 6007680) for 1 h at 37 °C. HTRF signals were obtained on a plate reader (Flexstation 3) at 665 nm (acceptor) and 620 nm (donor), and the 665/620 ratio was calculated with SoftMax Pro software.

Wire Myography

Mice were euthanized with CO_2, and the abdominal aorta or first-order mesenteric arteries were dissected. Arterial segments (2 mm in length) were mounted in wire myograph chambers (Danish Myo Technology, 610M) and maintained in freshly prepared Krebs–Henseleit buffer aerated with carbogen at 37 °C. After a 30-min recovery and normalization period, vessels were incubated with vehicle or compound (1 μM) for 1 h. Without washing, contractile responses were subsequently recorded by cumulative administration of the indicated agonists. Relaxation responses to iloprost, butaprost, isoprenaline, and sodium nitroprusside were assessed after precontraction with phenylephrine and expressed as percentages of maximal contraction. Contractile responses to AngII, phenylephrine, and U46619 were normalized to reference contractions induced by 60 mmol/l K^+ Krebs solution.

Mass spectrometry

Aliquots of 20 μl plasma samples were precipitated overnight with 95% acetonitrile at -20 °C. The supernatant was purified using a C18 HyperSep SPE column (Thermo Scientific). The evaporated eluent was reconstituted in 20 μl of 0.1% formic acid. Quantitative analyses were performed on an Orbitrap Q-exactive HF mass spectrometer (Thermo Scientific) equipped with an EASY-nLC capillary nanochromatography system (Thermo Scientific).1 μl of each sample was injected onto an in-house-packed capillary column (150 mm x 1.7 μm x 75 μm) with ReproSil-Pur 120 C18-AQ

resin (Dr. Maisch). A 30-min acetonitrile gradient at room temperature was used for sample separation. The mass spectrometer was operated in positive electrospray ionization (ESI) mode, and MS/MS data were collected in PRM (parallel reaction monitoring) analysis mode with a resolution of 60,000 for precursor mass spectra and 15,000 for tandem mass spectra. The collected data were integrated manually using Qual Browser (Thermo Scientific).

Histological analysis in IAV-infected lungs

Lung damage was quantified in H&E-stained lung sections using a blinded semi-automated FIJI/ImageJ macro. The macro first extracted the nuclear signal from the H&E image and generated a nuclear density map by tile-based local maxima detection and neighborhood density analysis. Total lung area was segmented separately to define the entire tissue section. Regions with high nuclear density were then identified as damaged areas, followed by optional manual removal of artifacts under blinded conditions. Finally, the damaged area was calculated as a percentage of the total lung section area.

Viral plaque assay

MDCKII cells (2.5×10^5 cells/well) were seeded in 12 well plate one day prior to the experiment. The monolayers were washed twice with PBS, and 200 μl of serially diluted bronchoalveolar lavage fluid was added to each well. Cells were infected for 1h at 37 °C, with gently rocking every 20 min to ensure even coverage and prevent drying. After infection, the inoculum was removed, and 1 ml of agar overlay medium containing 2 % agar (Serva, 11393.04) in H_2O and 2 ug/ml of TPCK-treated typsin (Thermo Fisher Scientific, 20233) was added. Plates were incubated at 37 °C with 5 % CO_2 for 3 days to allow plaques formation. Plaques were then counted.

4. FACS Gating Strategies

1. Gating strategy peritoneal leukocyte basal state (lymphoid cells)

Suppl. Fig. 8H

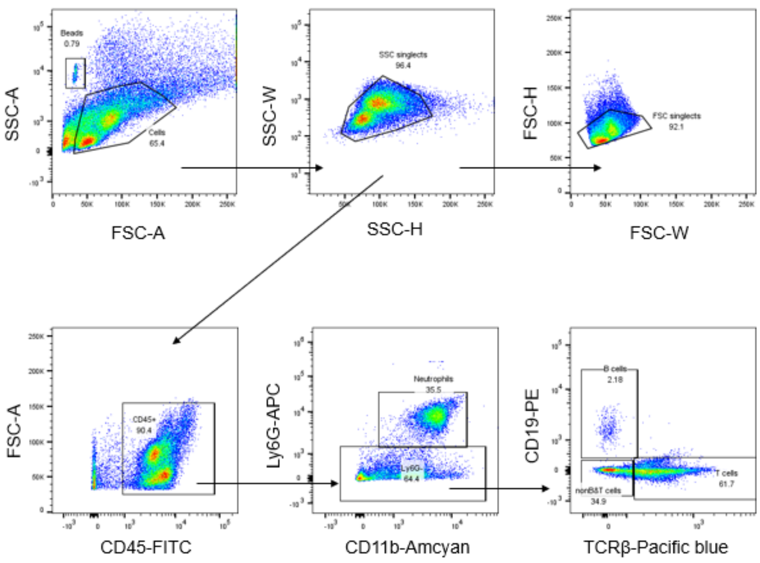

2. Gating strategy peritoneal leukocytes basal state and bacterial peritonitis (myeloid cells)

Figure 1I, J
Figure 2I-K
Figure 3N
Figure 7D
Suppl. Fig. 3E
Suppl. Fig. 6E, F
Suppl. Fig. 8H

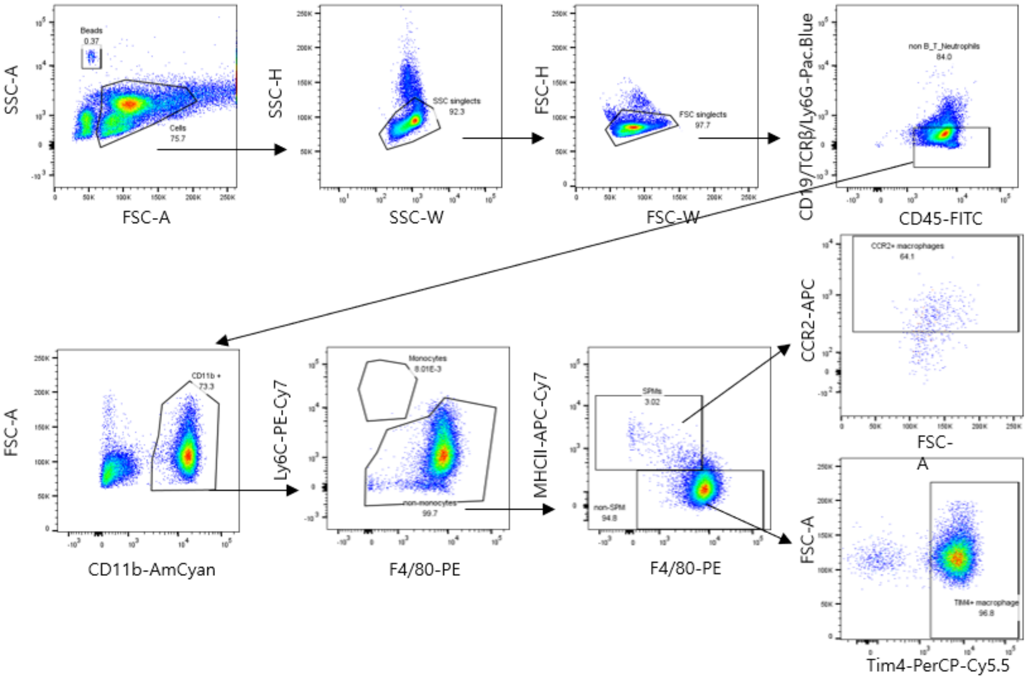

3. Gating strategy influenza virus infection (myeloid cells)

Figure 7G,H
Suppl. Fig. 9C, D
Suppl. Fig. 10C, D

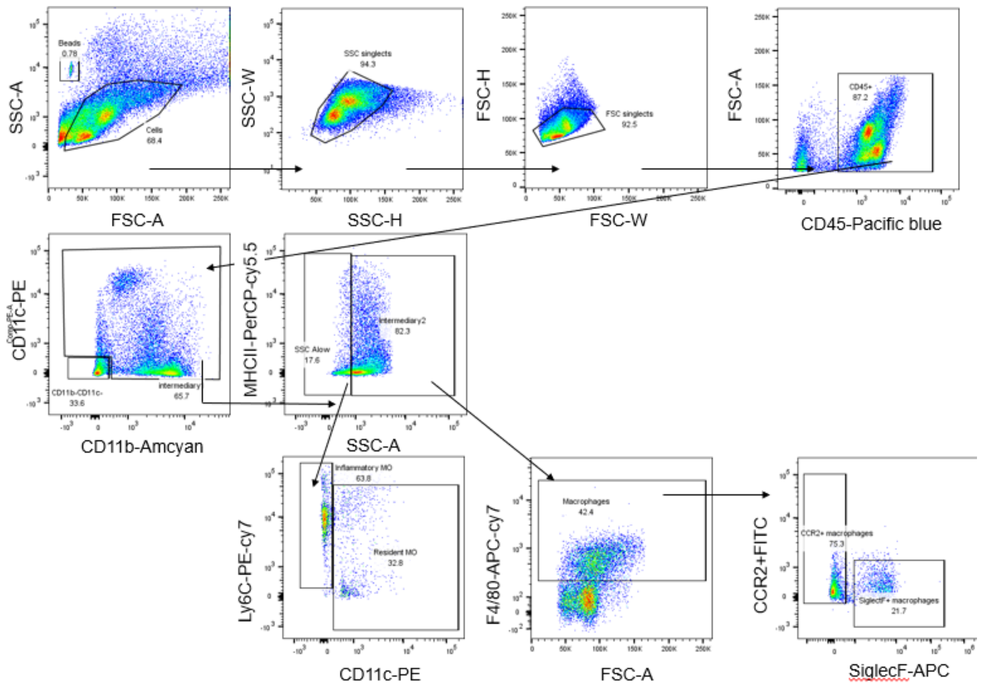

4. Gating strategy influenza virus infection (non-myeloid cells)

Suppl. Fig. 8J, K

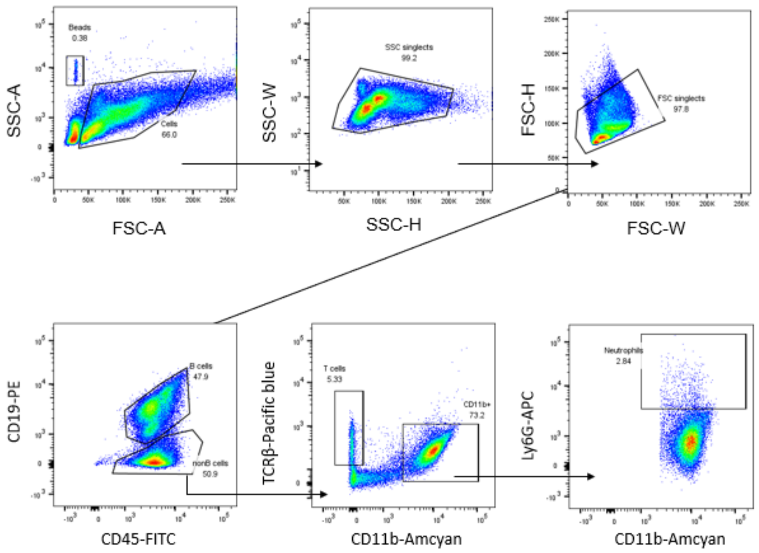

References

1. Zajd CM, Ziemba AM, Miralles GM, Nguyen T, Feustel PJ, Dunn SM, et al. Bone Marrow-Derived and Elicited Peritoneal Macrophages Are Not Created Equal: The Questions Asked Dictate the Cell Type Used. *Frontiers in immunology*. 2020;11:269.
2. Wang C, Yu X, Cao Q, Wang Y, Zheng G, Tan TK, et al. Characterization of murine macrophages from bone marrow, spleen and peritoneum. *BMC Immunol*. 2013;14:6.
3. Fu W, Franchini L, and Orlandi C. Comprehensive Spatial Profile of the Orphan G Protein Coupled Receptor GPRC5B Expression in Mouse Brain. *Front Neurosci*. 2022;16:891544.
4. Cui C, Schoenfelt KQ, Becker KM, and Becker L. Isolation of polymorphonuclear neutrophils and monocytes from a single sample of human peripheral blood. *STAR Protoc*. 2021;2(4):100845.
5. Lindner B, Burkard T, and Schuler M. Phagocytosis assays with different pH-sensitive fluorescent particles and various readouts. *Biotechniques*. 2020;68(5):245-50.
6. Bain CC, Hawley CA, Garner H, Scott CL, Schridde A, Steers NJ, et al. Long-lived self-renewing bone marrow-derived macrophages displace embryo-derived cells to inhabit adult serous cavities. *Nat Commun*. 2016;7:ncomms11852.
7. Vega-Perez A, Villarrubia LH, Godio C, Gutierrez-Gonzalez A, Feo-Lucas L, Ferriz M, et al. Resident macrophage-dependent immune cell scaffolds drive anti-bacterial defense in the peritoneal cavity. *Immunity*. 2021;54(11):2578-94 e5.
8. Baumann Z, Wiethe C, Vecchi CM, Richina V, Lopes T, and Bentires-Alj M. Optimized full-spectrum flow cytometry panel for deep immunophenotyping of murine lungs. *Cell Rep Methods*. 2024;4(11):100885.
9. Pei J, Kim BH, and Grishin NV. PROMALS3D: a tool for multiple protein sequence and structure alignments. *Nucleic Acids Res*. 2008;36(7):2295-300.
10. Wu H, Wang C, Gregory KJ, Han GW, Cho HP, Xia Y, et al. Structure of a class C GPCR metabotropic glutamate receptor 1 bound to an allosteric modulator. *Science*. 2014;344(6179):58-64.
11. Webb B, and Sali A. Comparative Protein Structure Modeling Using MODELLER. *Curr Protoc Protein Sci*. 2016;86:2 9 1-2 9 37.
12. Jumper J, Evans R, Pritzel A, Green T, Figurnov M, Ronneberger O, et al. Highly accurate protein structure prediction with AlphaFold. *Nature*. 2021;596(7873):583-9.
13. Waterhouse A, Bertoni M, Bienert S, Studer G, Tauriello G, Gumienny R, et al. SWISS-MODEL: homology modelling of protein structures and complexes. *Nucleic Acids Res*. 2018;46(W1):W296-W303.
14. de Vries SJ, van Dijk M, and Bonvin AM. The HADDOCK web server for data-driven biomolecular docking. *Nat Protoc*. 2010;5(5):883-97.
15. Kwon J, Kawase H, Mattonet K, Guenther S, Hahnefeld L, Shamsara J, et al. Orphan G protein-coupled receptor GPRC5B controls macrophage function by facilitating prostaglandin E receptor 2 signaling. *Nat Commun*. 2025;16(1):1448.
16. Coleman RG, Carchia M, Sterling T, Irwin JJ, and Shoichet BK. Ligand pose and orientational sampling in molecular docking. *PLoS One*. 2013;8(10):e75992.

17. Irwin JJ, Tang KG, Young J, Dandarchuluun C, Wong BR, Khurelbaatar M, et al. ZINC20-A Free Ultralarge-Scale Chemical Database for Ligand Discovery. *J Chem Inf Model*. 2020;60(12):6065-73.
18. Butina D. Unsupervised data base clustering based on Daylight's fingerprint and Tanimoto similarity: A fast and automated way to cluster small and large data sets. *J Chem Inf Comput Sci*. 1999;39(4):747-50.
19. Irwin JJ, Shoichet BK, Mysinger MM, Huang N, Colizzi F, Wassam P, et al. Automated docking screens: a feasibility study. *J Med Chem*. 2009;52(18):5712-20.
20. Eberhardt J, Santos-Martins D, Tillack AF, and Forli S. AutoDock Vina 1.2.0: New Docking Methods, Expanded Force Field, and Python Bindings. *J Chem Inf Model*. 2021;61(8):3891-8.
21. Baubet V, Le Mouellic H, Campbell AK, Lucas-Meunier E, Fossier P, and Brulet P. Chimeric green fluorescent protein-aequorin as bioluminescent Ca2+ reporters at the single-cell level. *Proc Natl Acad Sci U S A*. 2000;97(13):7260-5.
