# Supplementary material for: Small molecule inhibitor of orphan GPCR dimerization improves host defense and blood pressure control in mice
Source: J Clin Invest. 2026 Aug 3;136(15):e203162. doi: 10.1172/JCI203162 (PMC13430022; doi:10.1172/JCI203162)
Supplement: Supplemental data set 2 [file jci-136-203162-s099.docx]

**Supplemental Data File 2:**

**Comparison of the binding sites between the different models**

The binding site residues of three models and two published experimental GPRC5D structures (published in 2024), along with three templates, were compared. Structural alignment showed that GPRC5D is the most similar protein to GPRC5B in terms of binding pocket residue conservation. The alignment is shown in Figure 1.


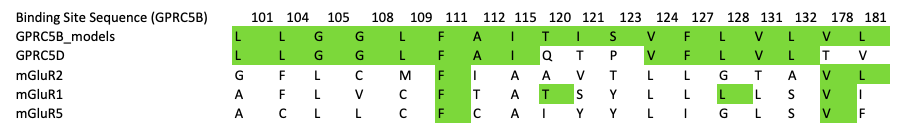


**Figure 1.** Structural alignment of GPRC5B binding site.

Based on this similarity, the conformations of the models were compared with the GPRC5D structure. Since two GPRC5D structures are available (PDB ID: 9IMA and 8YZK), the residues corresponding to the models’ binding sites were first compared with each other and are shown in Figure 2. As shown, two residues corresponding to F111 and L115 in GPRC5B exhibit flexibility, F78, I82, respectively.


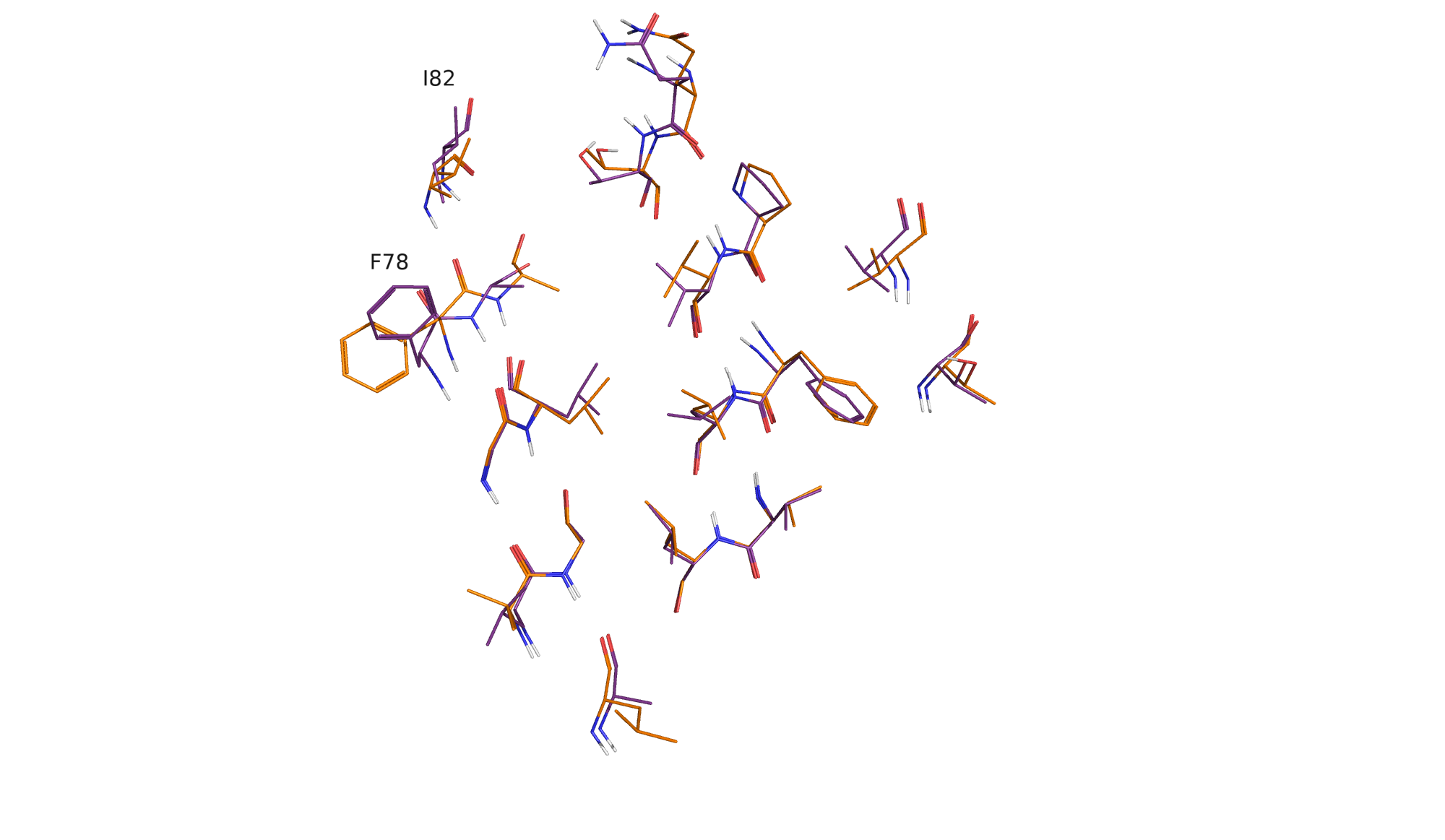


**Figure 2.** Superimposition of the binding sites of the two GPRC5D structures: 9IMA (orange) and 8YZK (purple).

The conformations of binding site residues in each model were then compared with GPRC5D. For non-conserved residues, RMSD values were calculated only for atom types present in both residues. As it is shown, non-conserved residues include 120, 121, 123, 178, and 181.

In all cases, binding pocket RMSD and individual residue RMSD were calculated after structural alignment using all heavy atoms (backbone and side chain) of predefined corresponding residues of the binding site, excluding hydrogen atoms, with Biopython ^1^.

Overall, the average RMSD difference among the three models is approximately 2 Å, indicating that the models are not significantly different. Although the AF model shows the best agreement with the GPRC5D structures, the main differences between SWISS-MODEL and AF, and to some extent MODELLER and AF, are mainly associated with either flexible residues or non-conserved residues. Therefore, it is difficult to determine which conformation is closest to the native structure for these particular residues.


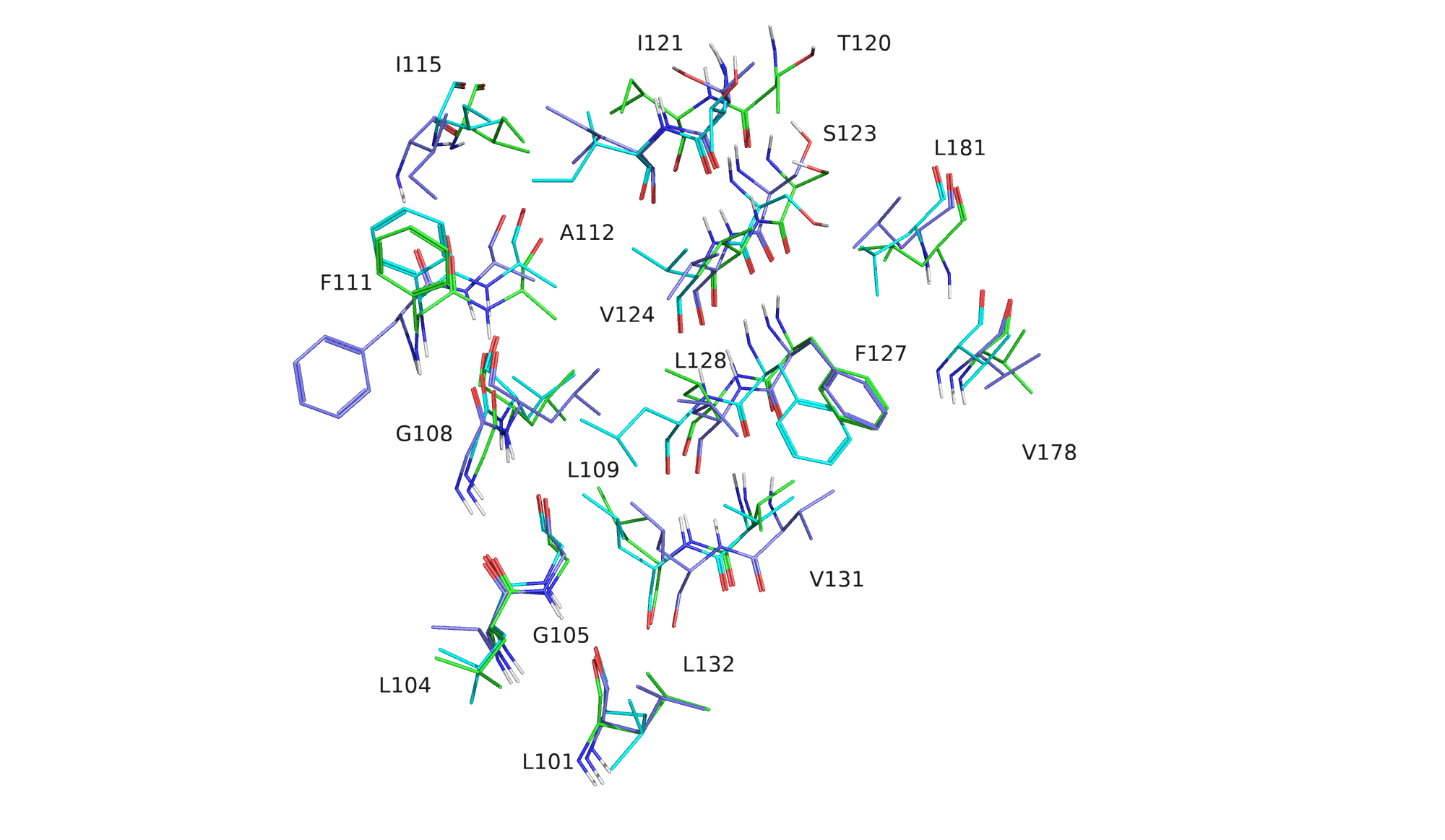


**Figure 3.** Superimposition of the binding sites of the three models: SWISS-MODEL (green), MODELLER (cyan), and AF (blue).

A structural alignment was performed with PyMOL (The PyMOL Molecular Graphics System, Version 3.1.0 Schrödinger, LLC) to compare the SWISS-MODEL structure with its respective template, mGluR2. SWISS-MODEL showed good consistency with the template residues at the binding site, with the exception of the flexible residue F111 and non-conserved residues, including, L101, L104, G105, L109, and F127 as shown in Figure 4.


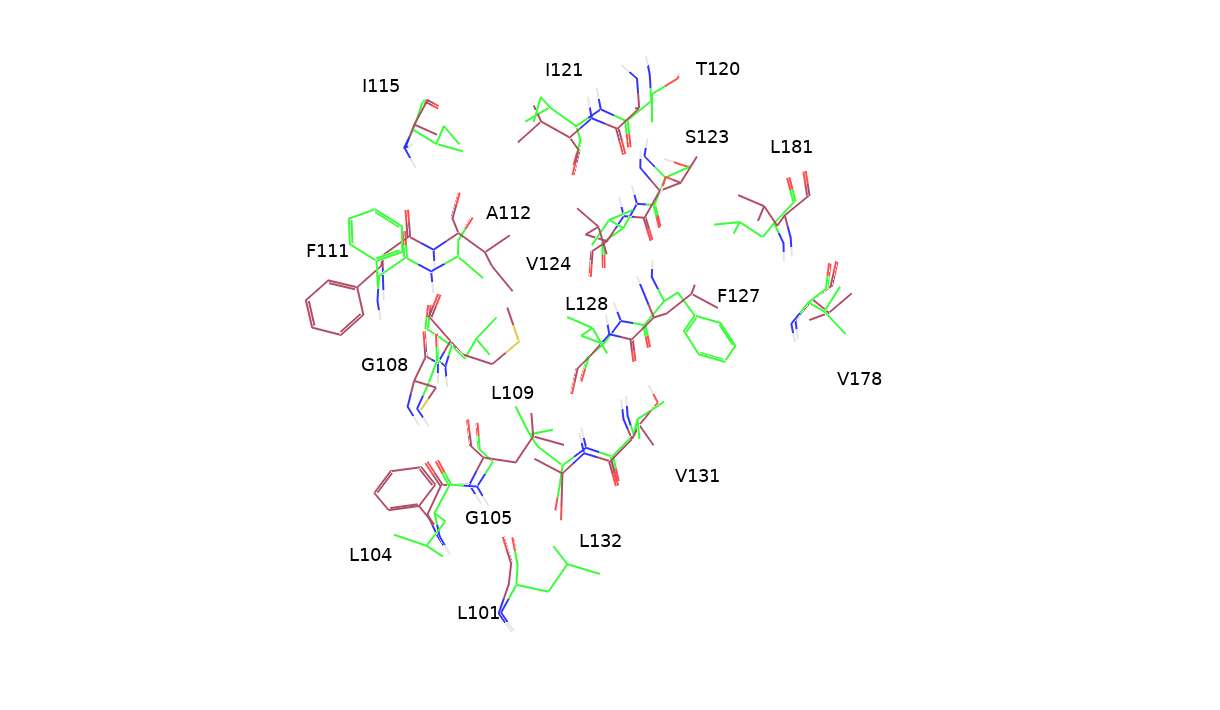


**Figure 4**. Superimposition of the binding site of the SWISS-MODEL structure (green) with its template mGluR2 (red) (PDB ID: 7MTS).

In the case of the MODELLER-generated structure, comparison with mGluR1 and mGluR5 templates revealed a similar trend to that observed for SWISS-MODEL. The model demonstrated good consistency with the template binding-site residues, except for the flexible F111 and non-conserved residues such as L104, G105, L109, S123, and F127 as shown in Figures 5 and 6.


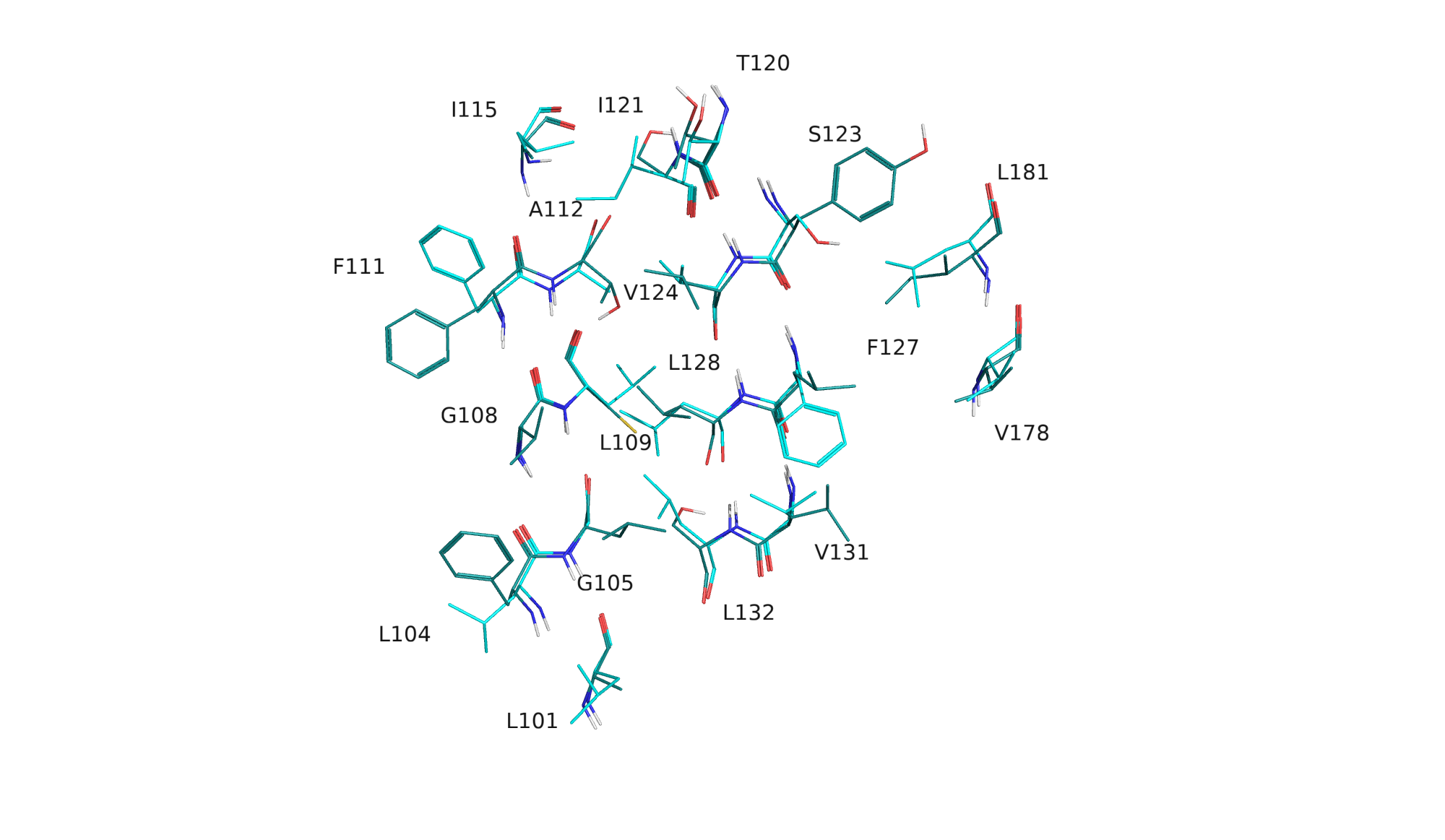


**Figure 5.** Superimposition of the binding site of the MODELLER structure (cyan) with its template mGluR1 (dark green) (PDB ID: 4OR2).


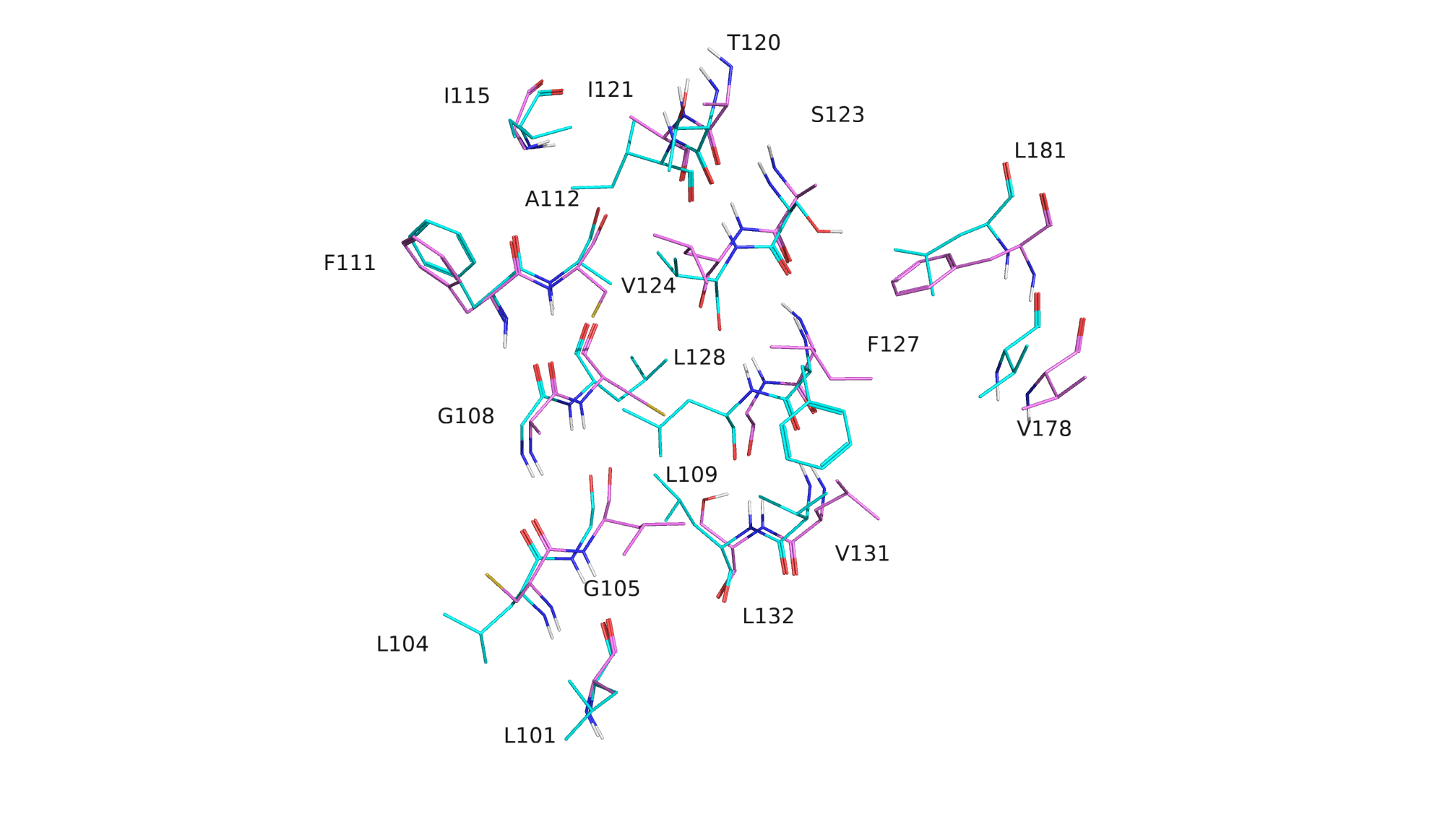


**Figure 6.** Superimposition of the binding site of the MODELLER structure (cyan) with its template mGluR5 (purple) (PDB ID: 6N52).

**In conclusion,** the average difference among the three models is relatively small (< 2 Å, Figure 7). GPRC5D represents the most similar available experimental structure for evaluating the model residue conformations, which were not available at the time of this study. The AF model shows the most similar conformation to GPRC5D. Most differences between the SWISS-MODEL and MODELLER models relative to AF are related to non-conserved and flexible residues. Exceptions include residue 132 in the SWISS-MODEL and residues 109, 127, 128, and 131 in the MODELLER model. Residue 101 in the AF model is not well modeled compared with GPRC5D. In addition, comparison of SWISS-MODEL and MODELLER with their corresponding templates showed that both models are in good agreement with the template structures regarding the side-chain conformations of the binding site residues.


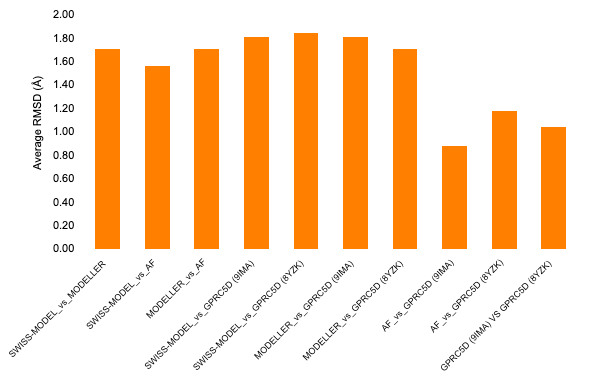


**Figure 7.** Average RMSD values calculated among the three models and between the models and the reference structures of GPRC5D for the binding site residues.

Residues showing significant conformational differences (RMSD > 2 Å) are summarized below:

SWISS-MODEL vs. MODELLER: Residues 120, 121, 123, 124, 127, and 128

SWISS-MODEL vs. AF: Residues 111, 115, 120, 121, and 132

MODELLER vs. AF:Residues 109, 111, 115, 127, 128, and 131

AF vs. GPRC5D (9IMA): Residues 101 and 120

SWISS-MODEL vs. GPRC5D (9IMA): Residues 101, 111, 115, 120, and 132

MODELLER vs. GPRC5D (9IMA): Residues 109, 111, 115, 120, and 128

AF vs. GPRC5D (8YZK): Residues 111, 115, and 120

SWISS-MODEL vs. GPRC5D (8YZK): Residues 111, 115, 120, 132, 178, and 181

MODELLER vs. GPRC5D (8YZK): Residues 111, 115, 120, and 128

1. Cock PJ, Antao T, Chang JT, Chapman BA, Cox CJ, Dalke A, Friedberg I, Hamelryck T, Kauff F, Wilczynski B and de Hoon MJ. Biopython: freely available Python tools for computational molecular biology and bioinformatics. *Bioinformatics*. 2009;25:1422-3.
