# Supplementary material for: Small molecule inhibitor of orphan GPCR dimerization improves host defense and blood pressure control in mice
Source: J Clin Invest. 2026 Aug 3;136(15):e203162. doi: 10.1172/JCI203162 (PMC13430022; doi:10.1172/JCI203162)
Supplement: Supplemental data set 4 [file jci-136-203162-s101.docx]

**Supplemental data 4:**

**Performance metrics of docking calculations**

**Table 1. Screening statistics Drug-Like ZINC20 subset**

|  | **JS model** | **MP model** | **AF model** |
| --- | --- | --- | --- |
| Total Drug-like screened | 27584708 | 27584708 | 27584708 |
| Clustered | 704 | 714 | 923 |
| Extracted | 8855023 | 8750455 | 8804688 |
| Molecules with 0 poses | 1699448 | 3748997 | 3637908 |
| Inspected poses | 704 | 714 | 923 |
| Selected for “Hit Picking Party” | 5 | 6 | 15 |
| Position MP20 in extracted poses | 1751 | 2829825 | 770020 |
| Position MP20 in clustered poses | 30 | N.A | N.A |

**Table 2. Screening statistics Lead-Like ZINC20 subset**

|  | **JS model** | **MP model** | **AF model** |
| --- | --- | --- | --- |
| Total Lead-like screened | 7526836 | 7526836 | 7526836 |
| Clustered | 887 | 1021 | 1062 |
| Extracted | 2688662 | 2668696 | 2676840 |
| Molecules with 0 poses | 286894 | 553355 | 247827 |
| Inspected poses | 887 | 1062 | 1021 |
| Selected for “Hit Picking Party” | 2 | 5 | 14 |
| Position MP8 in extracted poses | 141302 | 73748 | 3316 |
| Position MP8 in clustered poses | N.A | N.A | 206 |
| Position MP11 in extracted poses | 687570 | 791354 | 1979 |
| Position MP11 in clustered poses | N.A | N.A | 820 |

**Table 3. Dataset for Enrichment calculation**

|  | **JS model** | **MP model** | **AF model** |
| --- | --- | --- | --- |
| Docked Decoys | 76 | 76 | 76 |
| Decoys with 0 poses | 47 | 45 | 50 |
| Total actives | 3 | 3 | 3 |
| Failed actives poses | 0 | 0 | 0 |
| Decoys extracted poses | 18 | 18 | 16 |
| Actives extracted Poses | 1 | 1 | 1 |

**Table 4. Hit rates**

| Tested compounds | 13 |
| --- | --- |
| Active compounds | 3 |
| Hit compound | 1 |
| Hit rate % (Hit/total inspected poses) | 0,06 |
| Hit rate % (Actives/total inspected poses) | 0,02 |
| Hit rate % (Actives/tested) | 23,08 |
| Hit rate % (Hit/tested) | 7,69 |
