# Supplementary material for: Small molecule inhibitor of orphan GPCR dimerization improves host defense and blood pressure control in mice
Source: J Clin Invest. 2026 Aug 3;136(15):e203162. doi: 10.1172/JCI203162 (PMC13430022; doi:10.1172/JCI203162)

Figure 1B

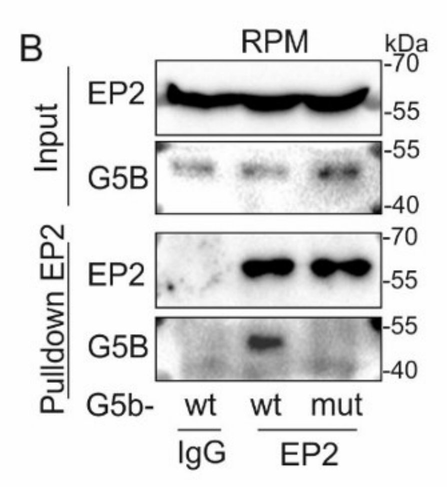

Input PTGER2

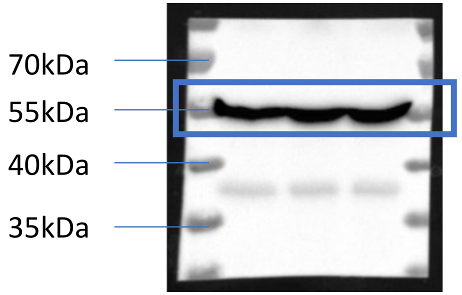

Pulldown PTGER2

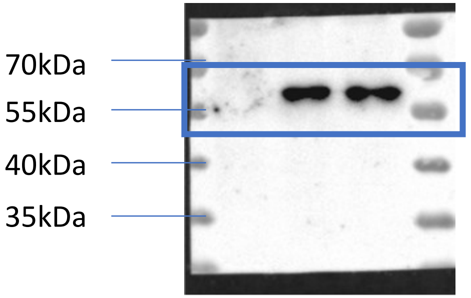

Input GPRC5B

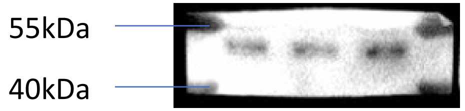

Pulldown GPRC5B

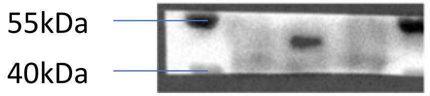

Figure 2B

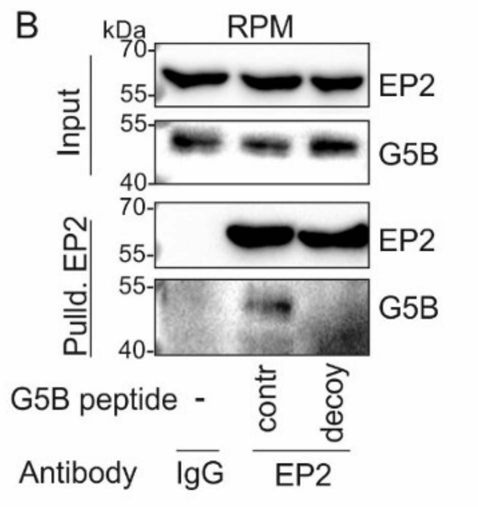
B
kDa
RPM
70
55
EP2
55
40
G5B
Input
70
55
EP2
55
40
G5B
Pulld. EP2
G5B peptide
-
contr
decoy
Antibody
IgG
EP2

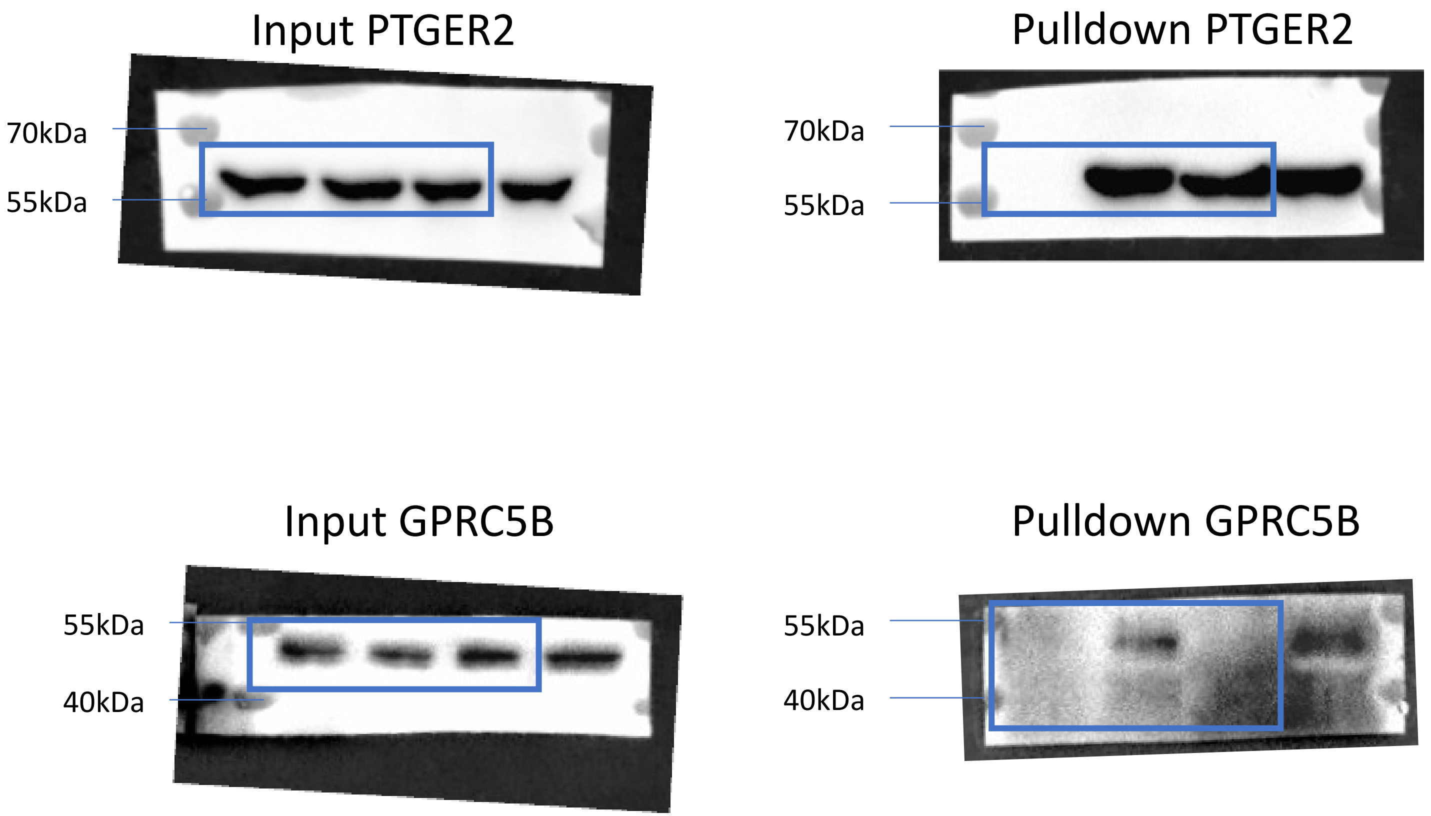
Input PTGER2
70kDa
55kDa
Pulldown PTGER2
70kDa
55kDa
Input GPRC5B
55kDa
40kDa
Pulldown GPRC5B
55kDa
40kDa

Figure 3A

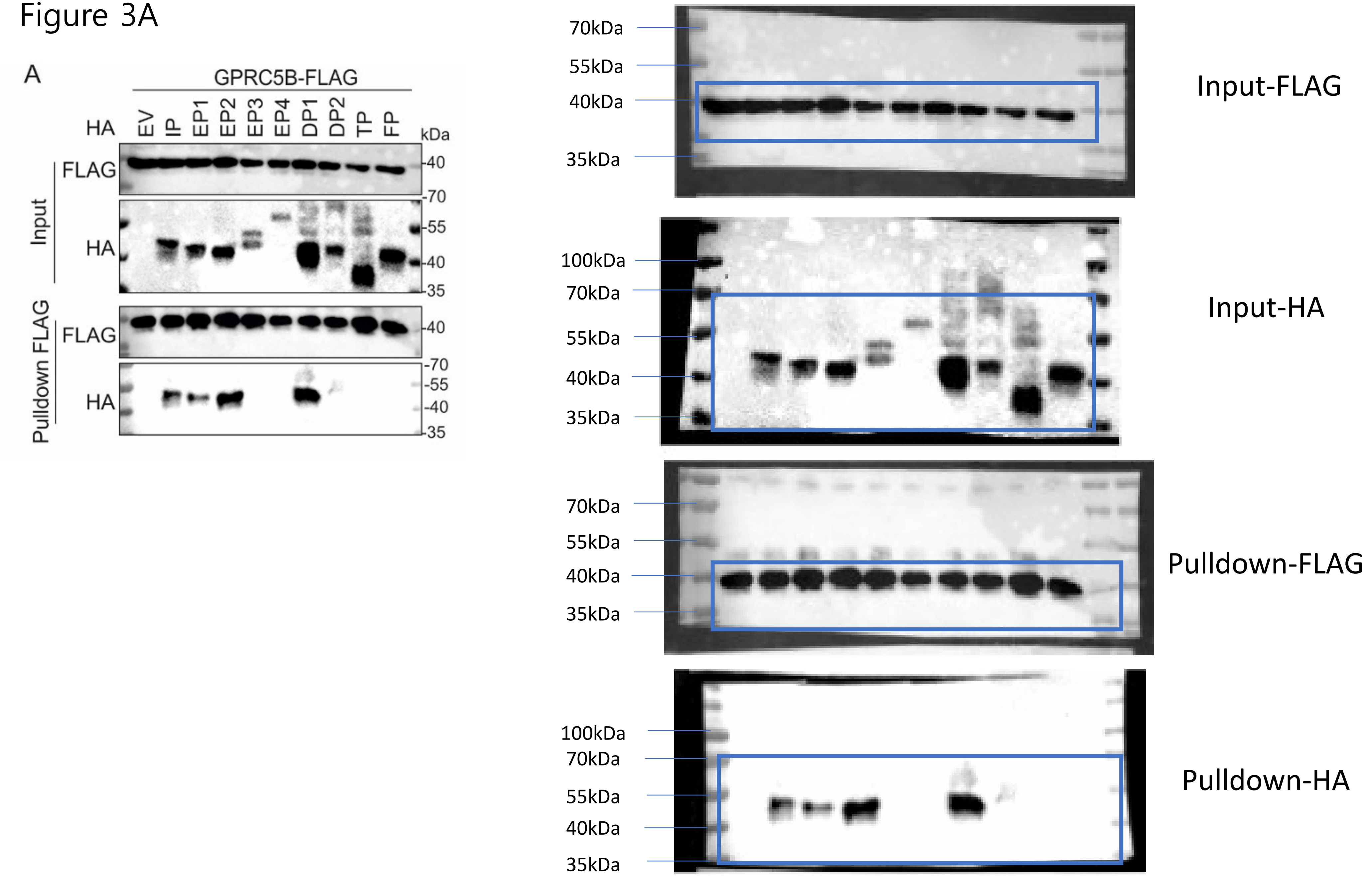

A
GPRC5B-FLAG
HA
EV
IP
EP1
EP2
EP3
EP4
DP1
DP2
TP
FP
kDa
Input
FLAG
HA
Pulldown FLAG
FLAG
HA
40
70
55
40
35
40
70
55
40
35
70kDa
55kDa
40kDa
35kDa
Input-FLAG
100kDa
70kDa
55kDa
40kDa
35kDa
Input-HA
70kDa
55kDa
40kDa
35kDa
Pulldown-FLAG
100kDa
70kDa
55kDa
40kDa
35kDa
Pulldown-HA

Figure 3B

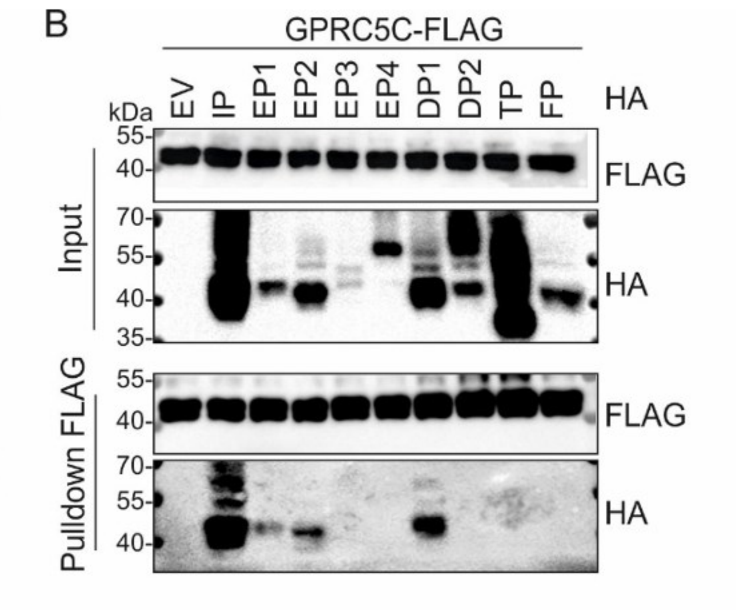
B
GPRC5C-FLAG
kDa
EV
IP
EP1
EP2
EP3
EP4
DP1
DP2
TP
FP
HA
55
40
FLAG
Input
70
55
40
35
HA
Pulldown FLAG
55
40
FLAG
70
55
40
HA

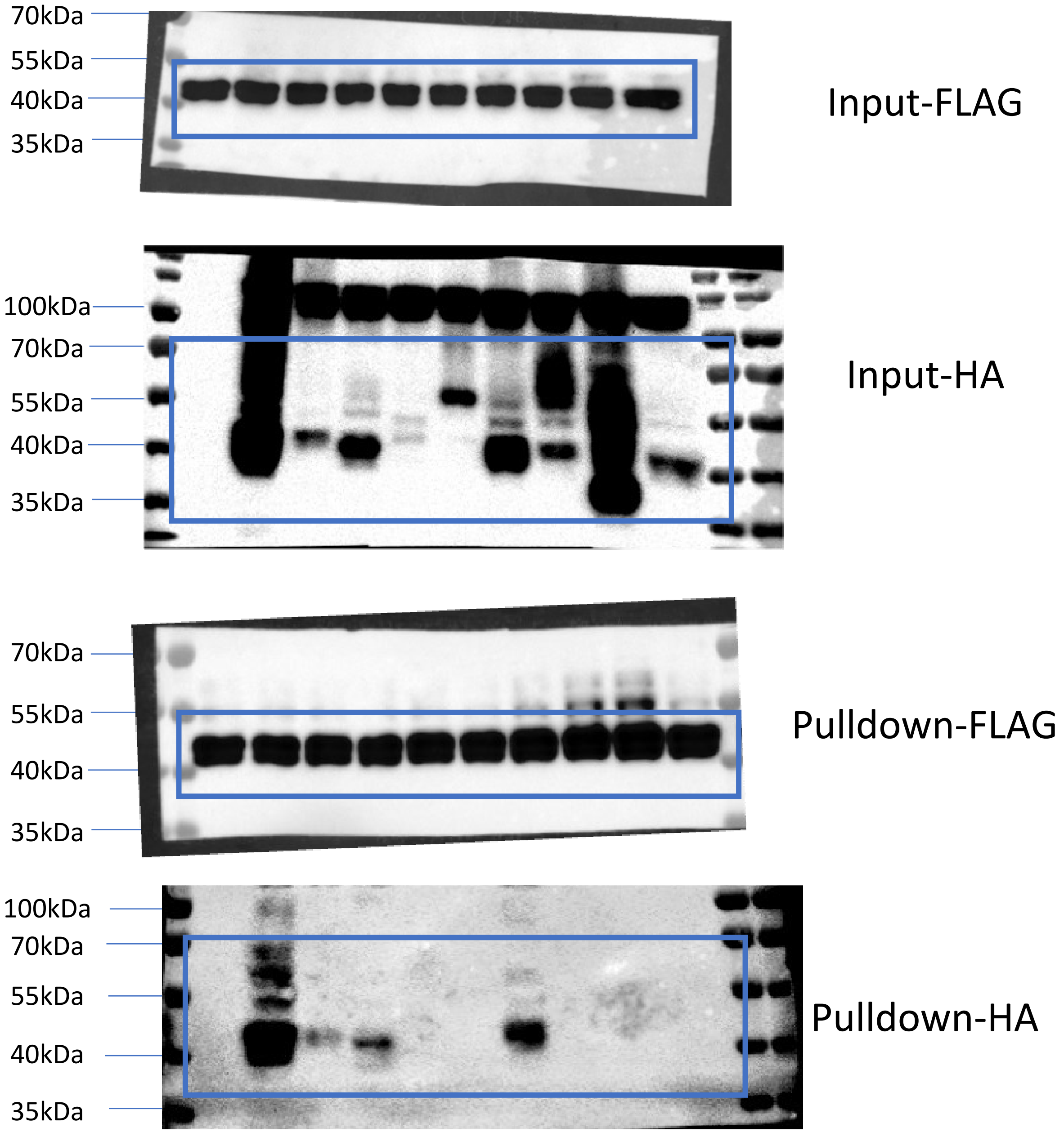
70kDa
55kDa
40kDa
35kDa
Input-FLAG
100kDa
70kDa
55kDa
40kDa
35kDa
Input-HA
70kDa
55kDa
40kDa
35kDa
Pulldown-FLAG
100kDa
70kDa
55kDa
40kDa
35kDa
Pulldown-HA

Figure 4C

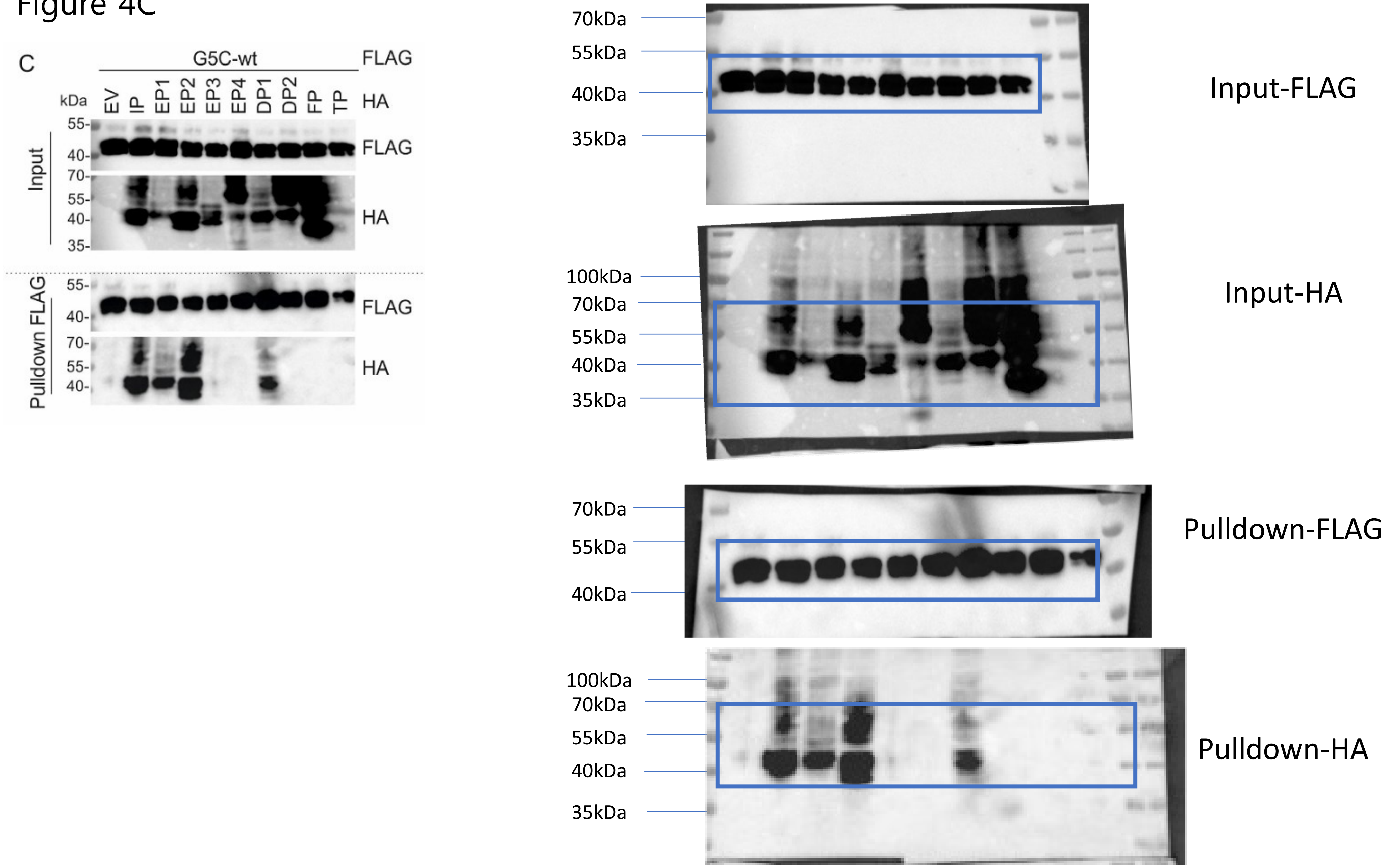

C
G5C-wt
FLAG
kDa
EV
IP
EP1
EP2
EP3
EP4
DP1
DP2
FP
TP
HA
Input
Pulldown FLAG
FLAG
HA
70kDa
55kDa
40kDa
35kDa
Input-FLAG
100kDa
Input-HA
Pulldown-FLAG
Pulldown-HA

Figure 4D

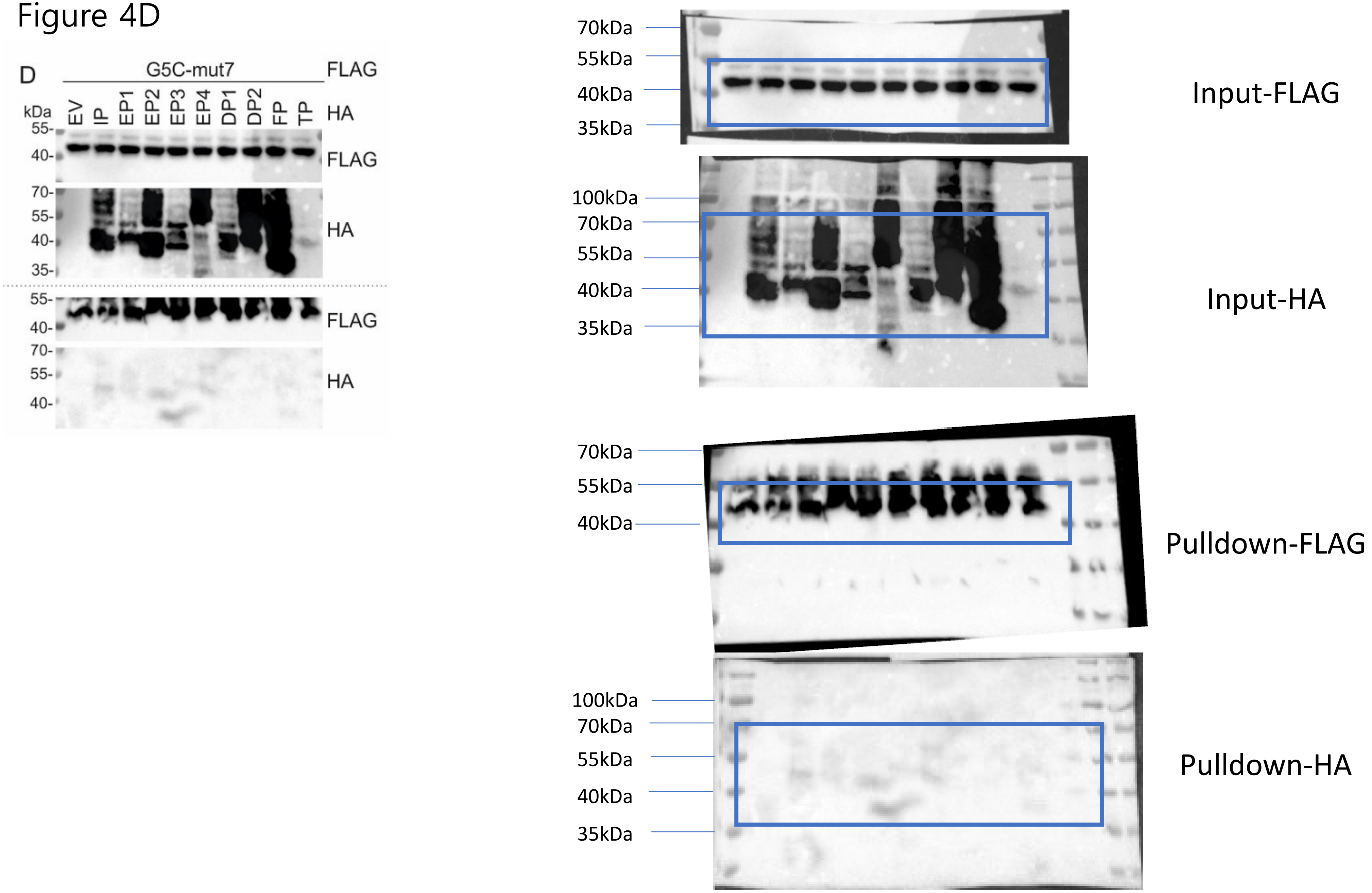
D
G5C-mut7
FLAG
HA
kDa
EV
IP
EP1
EP2
EP3
EP4
DP1
DP2
FP
TP
55-
40-
FLAG
70-
55-
40-
35-
HA
55-
40-
FLAG
70-
55-
40-
HA
70kDa
55kDa
40kDa
35kDa
Input-FLAG
100kDa
70kDa
55kDa
40kDa
35kDa
Input-HA
70kDa
55kDa
40kDa
Pulldown-FLAG
100kDa
70kDa
55kDa
40kDa
35kDa
Pulldown-HA

Figure 6E

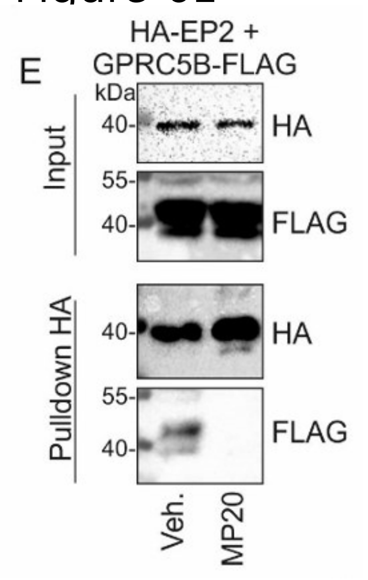

Input HA

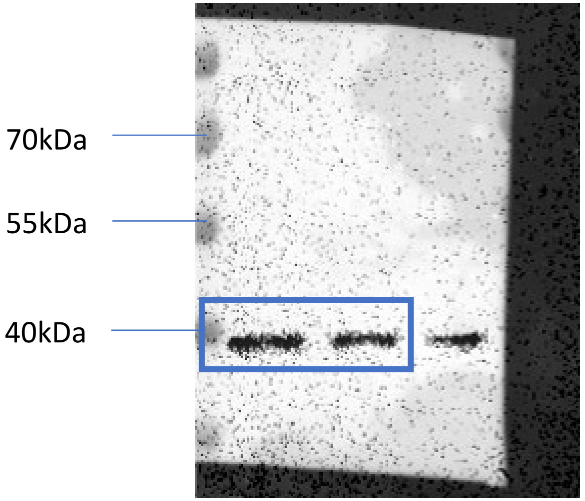

Pulldown HA

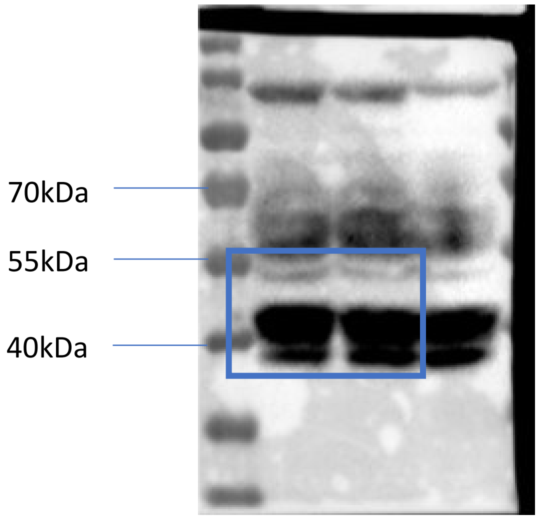

Input FLAG

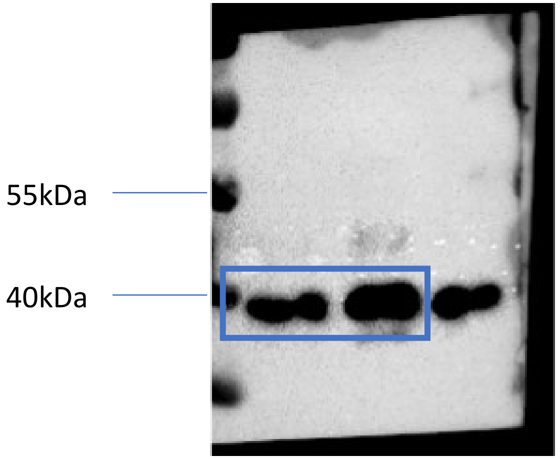

Pulldown FLAG

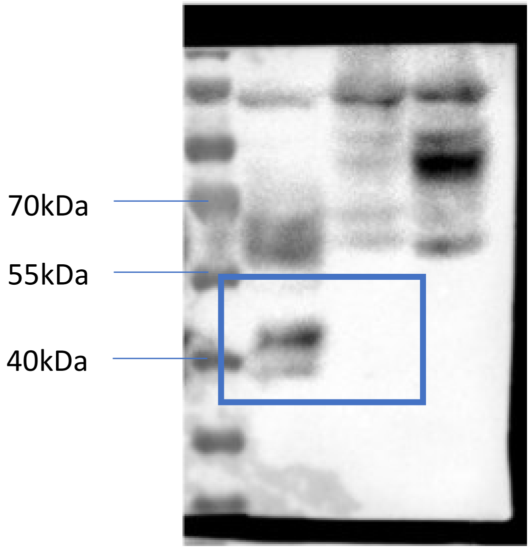

Figure 6L

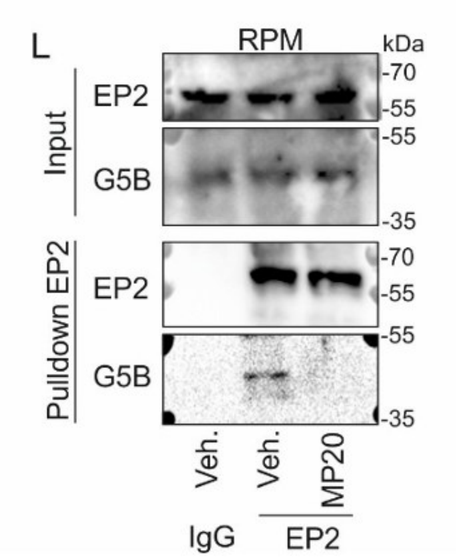

Input PTGER2

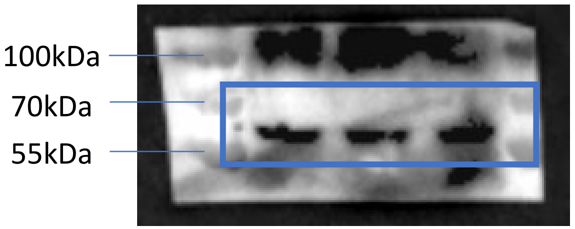

Pulldown PTGER2

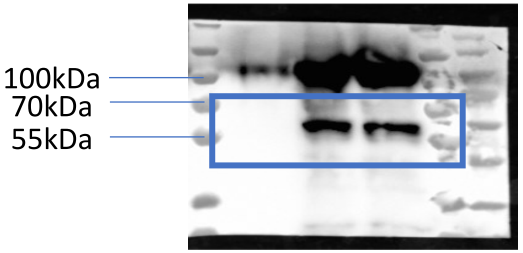

Input GPRC5B

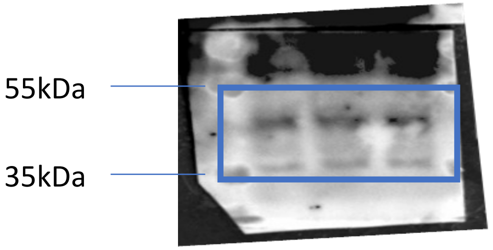

Pulldown GPRC5B

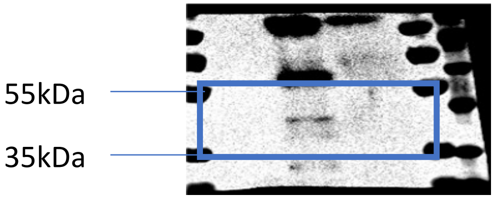

Figure 8B

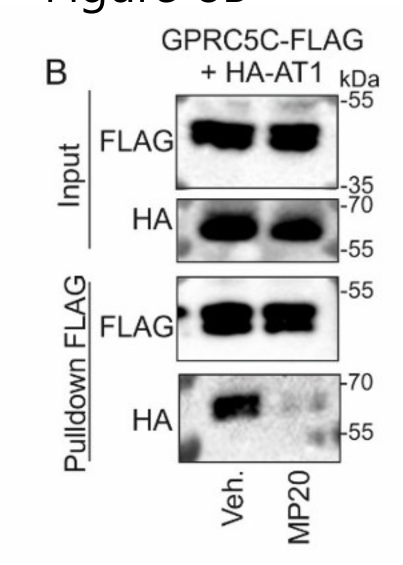

Input HA

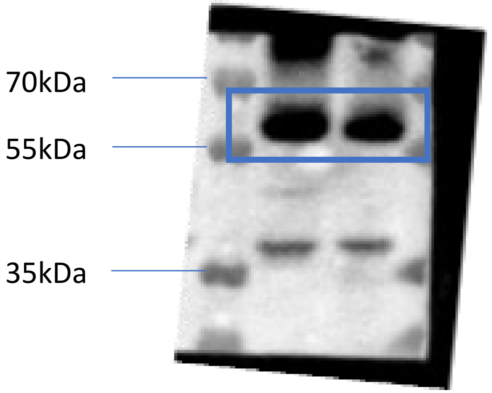

Pulldown HA

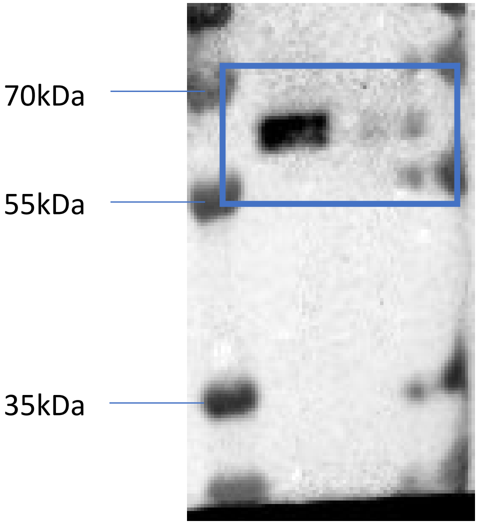

Input FLAG

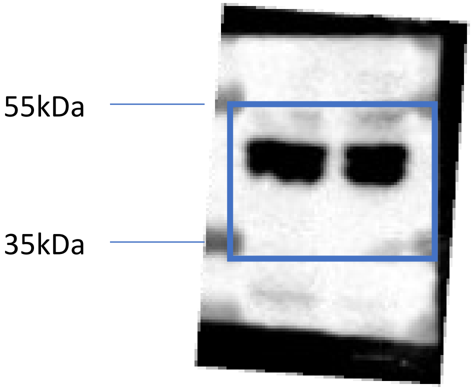

Pulldown FLAG

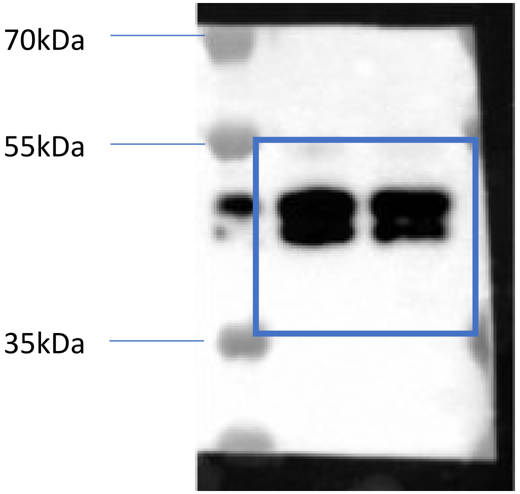

Figure 8I

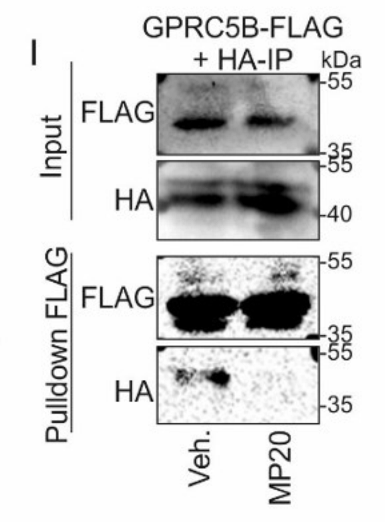

Input HA

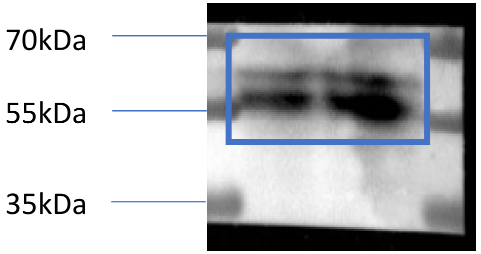

Pulldown HA

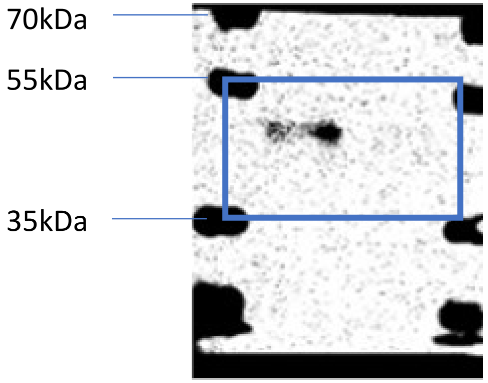

Input FLAG

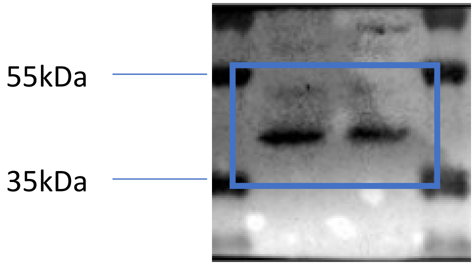

Pulldown FLAG

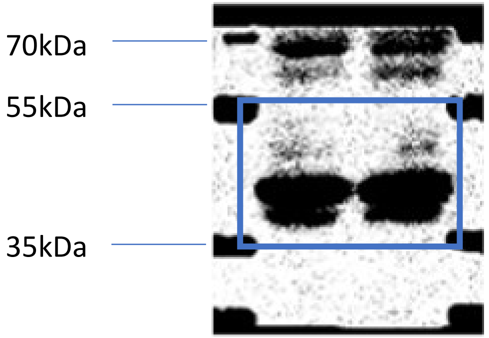

Supplemental Figure 2C

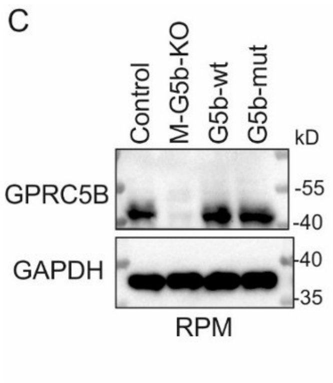

GPRC5B

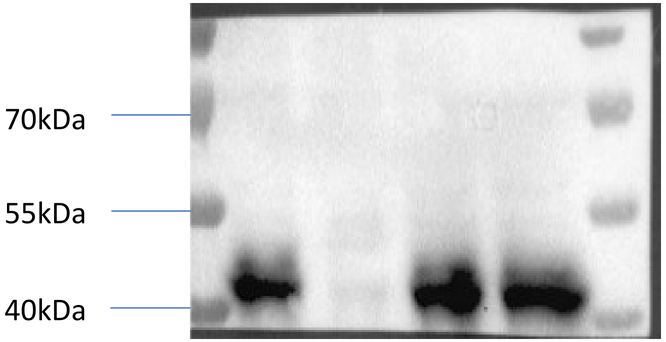

GAPDH

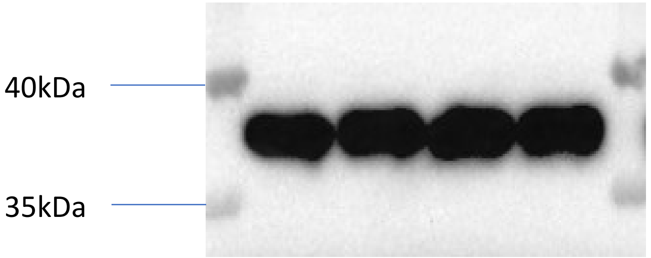

Supplemental Figure 5A

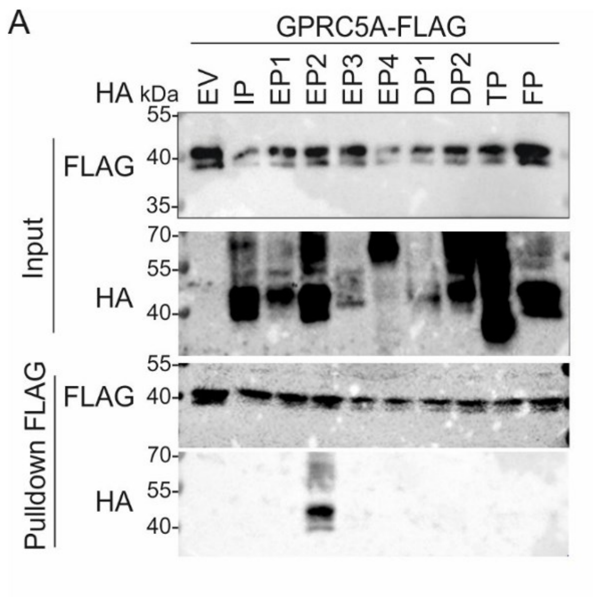

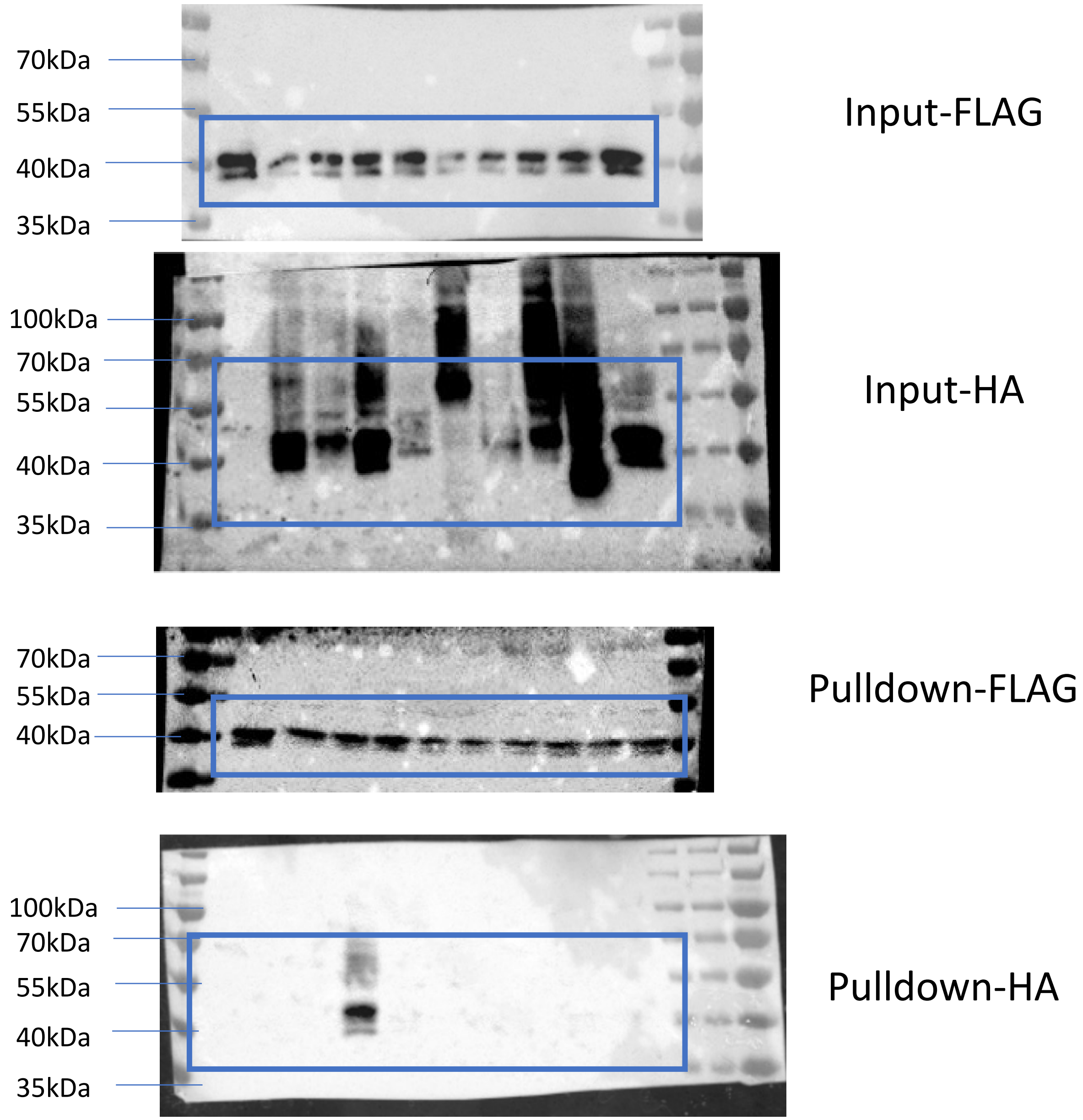

Supplemental Figure 5B

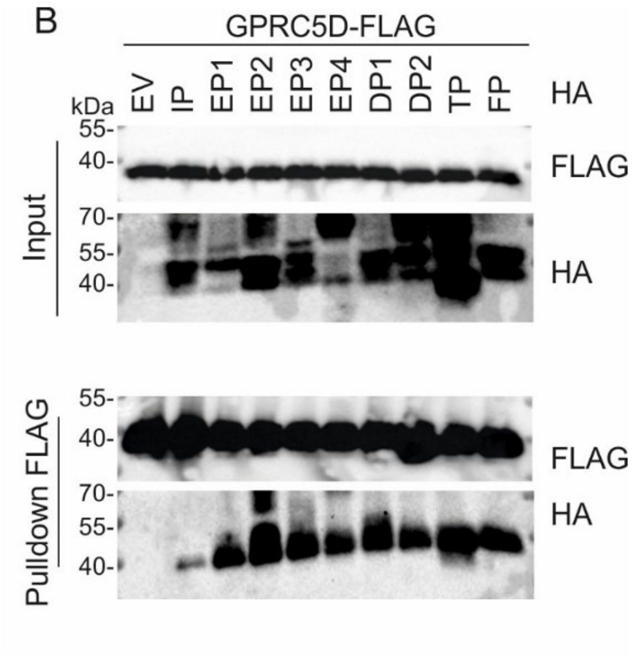

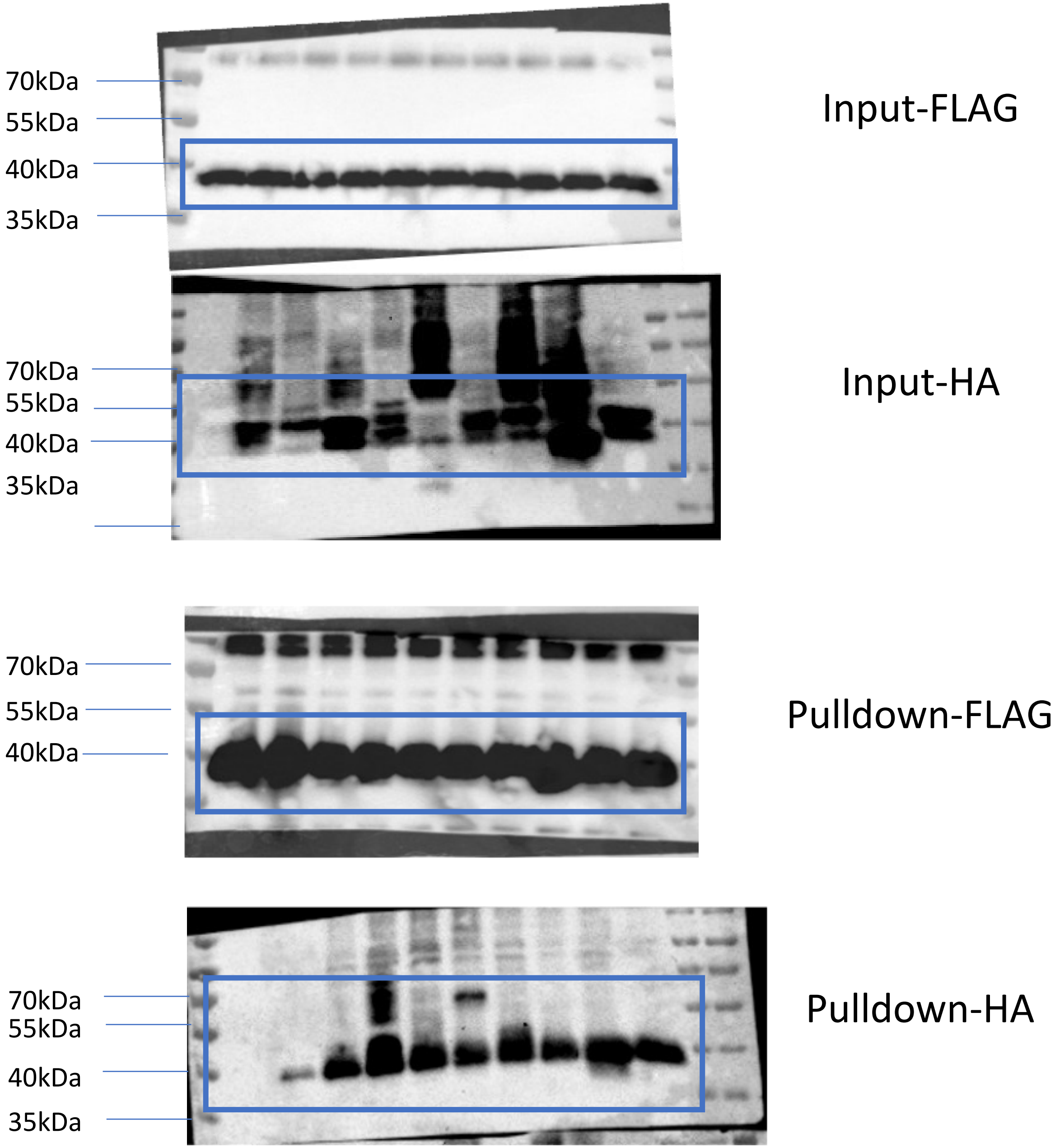

Supplemental Figure 7B

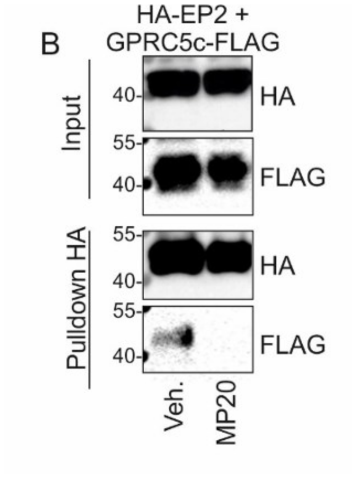

Input HA

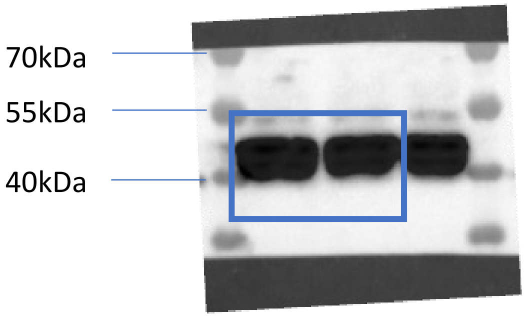

Pulldown HA

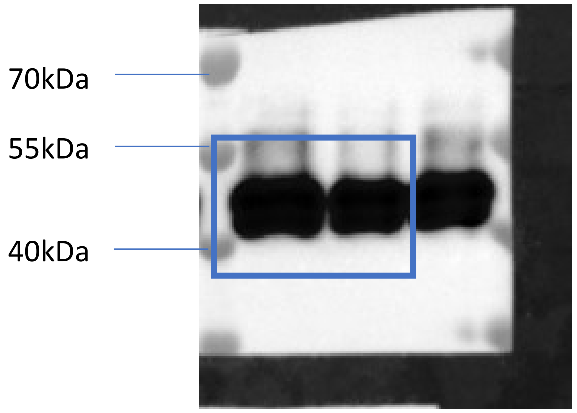

Input FLAG

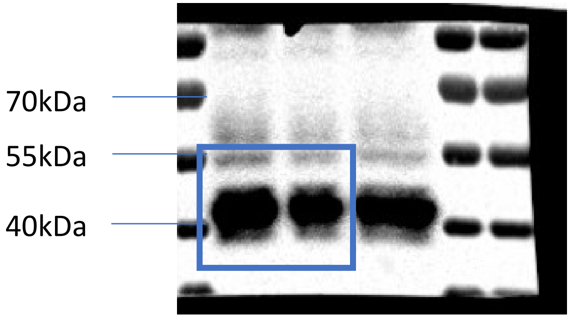

Pulldown FLAG

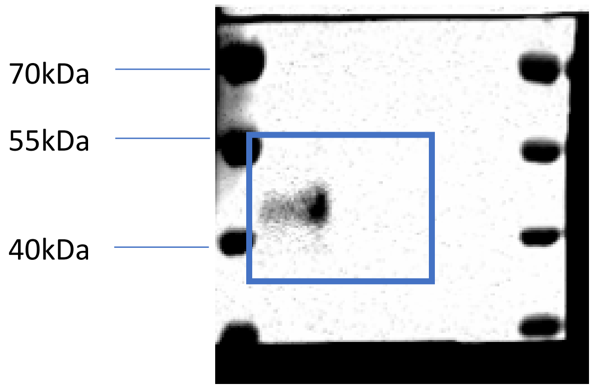

Supplement: Unedited blot and gel images [file jci-136-203162-s102.pdf]
